# Discovery of VU6053371/BI03738809: A First-in-Class Selective and CNS-Penetrant mGlu_3_ Positive Allosteric Modulator (PAM) with Efficacy in a Preclinical Cognition Model

**DOI:** 10.1021/acschemneuro.6c00325

**Published:** 2026-06-06

**Authors:** Caleb A. Jones, Renn A. Duncan, Kristen M. Gilliland, Carson W. Reed, Daniel H. Haymer, Paul K. Spearing, Rory A. Capstick, Bartholomew P. Roland, Paige Vinson, Marc Quitalig, Caroline Baggeroer, Natasha B. Billard, Jonathan W. Dickerson, Zixiu Xiang, Valerie M. Kramlinger, Olivier Boutaud, Julia Schlichtiger, Carrie K. Jones, Colleen M. Niswender, Hyekyung P. Cho, Jerri L. Rook, Carsten T. Wotjak, Matthias Freiwald, Riccardo Giovannini, Heiko Sommer, Scott Hobson, P. Jeffrey Conn, Bruce J. Melancon, Craig W. Lindsley

**Affiliations:** † Warren Center for Neuroscience Drug Discovery, Vanderbilt Institute for Therapeutic Advances, 5718 Vanderbilt University, Nashville, Tennessee 37232, United States; ‡ Department of Pharmacology, 12327 Vanderbilt University School of Medicine, Nashville, Tennessee 37232, United States; § Department of Chemistry, Vanderbilt University, Nashville, Tennessee 37232, United States; ∥ Vanderbilt Kennedy Center, 12328 Vanderbilt University Medical Center, Nashville, Tennessee 37232, United States; ⊥ Vanderbilt Brain Institute, Vanderbilt University, Nashville, Tennessee 37232, United States; # 33437 Boehringer Ingelheim Pharma GmbH & Co. KG, Birkendorfer Str. 65, 88397 Biberach, Germany

**Keywords:** metabotropic glutamate receptor subtype 3 (mGlu_3_), positive allosteric modulator (PAM), cognition, metabolism, novel object recognition, pharmacokinetics

## Abstract

Herein, we report the discovery and development of the first-in-class (FIC), selective, and centrally active metabotropic glutamate receptor subtype 3 (mGlu_3_) positive allosteric modulator (PAM), VU6053371/BI03738809. A high-throughput screening campaign identified a potent and selective mGlu_3_ PAM VU6048261/DI013166572 based on a tetra-substituted thiophene core but with poor DMPK properties. Chemical lead optimization efforts managed to dramatically improve protein binding and *in vivo* rat PK to afford VU6053371/BI03738809. With an FIC *in vivo* tool compound, VU6053371/BI03738809 demonstrated robust efficacy in rat novel object recognition (NOR) (minimum effective dose (MED) = 3 mg/kg PO) and a clear pharmacokinetic/pharmacodynamic (PK/PD) relationship (PD efficacy observed when free brain concentrations were at, or above, the rat mGlu_3_ EC_50_). Thus, selective activation of mGlu_3_ represents a novel mechanism to address the cognitive impairment associated with schizophrenia (CIAS) and other neurodegenerative diseases. Moreover, the discovery of VU6053371/BI03738809 completes the group II mGlu receptor toolkit of *in vivo* PAM and NAM probes for both mGlu_2_ and mGlu_3_.

## Graphical abstract

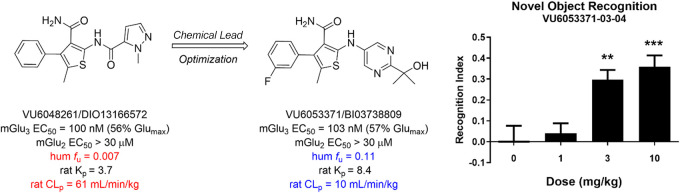

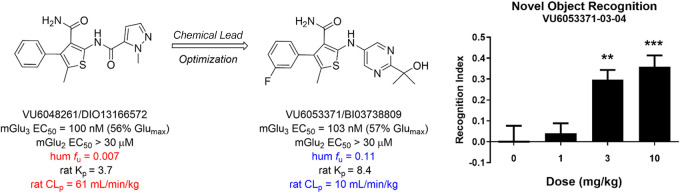

## Introduction

There is a great unmet medical need for novel therapeutic strategies to address the negative and cognitive symptom cluster of schizophrenia, symptoms that are poorly addressed by clinically available typical and atypical antipsychotics.^1–3^ The metabotropic glutamate receptor subtype 3 (mGlu_3_) has emerged as an exciting novel target,^4,5^ as human genetic studies have identified links between polymorphisms in *GMR3*, the gene that encodes mGlu_3_, with both schizophrenia and cognitive dysfunction.^6–10^ In addition, new studies have established that activation of mGlu_3_ exerts its actions on synaptic plasticity (long-term depression (LTD) and long-term potentiation (LTP)) via downstream mGlu_5_.^9^ In the absence of selective mGlu_3_ positive allosteric modulator (PAMs), the field has employed a dual mGlu_2/3_ agonist (LY379268), or dual mGlu_2/3_ PAMs, in combination with an mGlu_2_ NAM (e.g., VU6001966) (structures shown in Figure 1) to isolate the activity of mGlu_3_ to reveal effects on synaptic plasticity in the hippocampus and reversal of phencyclidine (PCP)-induced pharmacological challenges.^10,11^ Recently, this same pharmacological strategy rescued PCP-induced changes in excitatory transmission in the nucleus accumbens (NAc) as well as sociability deficits, highlighting that selective mGlu_3_ activation could address both cognitive and negative symptoms in schizophrenia.^10,11^ In the literature, mGlu_3_ has emerged as a “new hope” for the treatment of schizophrenia,^1^ but fulfillment of this hope has been hampered by a complete lack of selective mGlu_3_ activators. To address this notable shortcoming, we now detail the discovery and development of the first selective and centrally active mGlu_3_ positive allosteric modulator (PAM), VU6053371/BI03738809, that displays robust efficacy in native tissue electrophysiology synaptic plasticity assays as well as in a rat novel object recognition (NOR) assay with a clear pharmacokinetic/pharmacodynamic (PK/PD) relationship. Thus, selective activation of mGlu_3_ represents a novel mechanism to address cognitive impairment associated with schizophrenia (CIAS)^12^ and other neurodegenerative diseases. Moreover, the discovery of VU6053371/BI03738809 completes the Group II mGlu receptor pharmacological toolkit of *in vivo* PAM and NAM probes for both mGlu_2_ and mGlu_3_.

**Figure 1:**
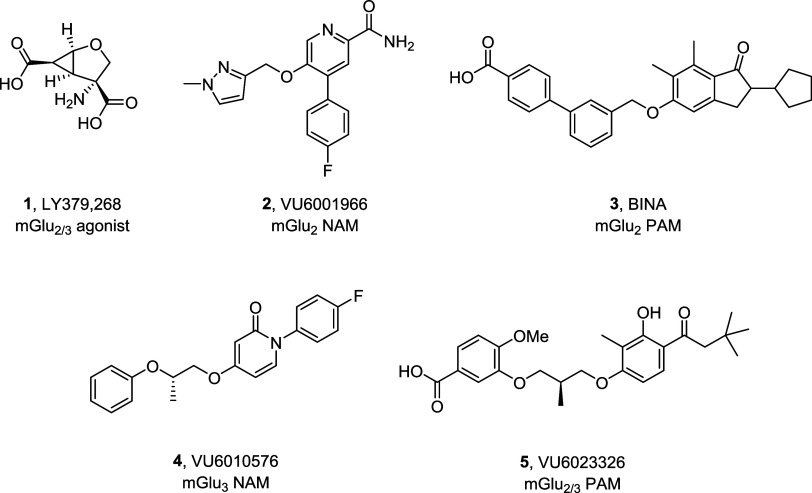
Structures of representative selective group II mGlu receptor dual orthosteric mGlu_2/3_ agonist LY379268 (**1**), selective mGlu_2_ NAM VU6001966 (**2**), selective mGlu_2_ PAM BINA (**3**), selective mGlu_3_ NAM VU601576 (**4**), and dual mGlu_2/3_ PAM VU6023326 (**5**). At present, there are no reported mGlu_3_ PAMs.

## Results and Discussion

### High-Throughput Screening

A high-throughput screen was performed on the Boehringer Ingelheim sample collection employing a cAMP (cyclic adenosine 3′, 5′-monophosphate) assay in Chinese Hamster Ovary (CHO) cells expressing human mGlu_3_ in PAM mode to detect receptor activation.^13^ From this screen, an interesting tetra-substituted thiophene carboxamide, DI01316572/VU6048261 (**6**) was identified (Figure 2). In the cAMP assay, **6** proved to be a robust human mGlu_3_ PAM (EC_50_ = 130 nM, 73% Glu Max) and inactive on human mGlu_2_ (cAMP EC_50_ > 30 μM). Transitioning to the workhorse kinetic GIRK assay (assessing thallium flux as a surrogate for mGlu_3_ activation),^13,14^ the selective mGlu_3_ PAM activity was confirmed (GIRK human mGlu_3_ EC_50_ = 100 nM, 56% Glu Max; GIRK human mGlu_2_ EC_50_ > 30 μM). Thus, we were able to identify the first selective mGlu_3_ PAM; however, additional studies were required to assess its suitability as a hit for further optimization. PAM **6** was a low-molecular-weight compound (340) with a reasonable TPSA (90) and a favorable CNS MPO score (5.4). In terms of *in vitro* DMPK, PAM **6** was highly protein-bound in plasma (h,r *f*_u_ = 0.007, 0.018, respectively) and rat brain (*f*_u_ = 0.005). However, PAM **6** was predicted to be CNS penetrant in human (MDCK P-gp ER = 0.5; *P*_app_ = 30 × 10^–6^ cm/s), displayed modest microsomal stability (∼50% Q_H_ in human and rat liver microsomes), and a clean CYP_450_ profile (>50 μM vs 3A4, 2D6, 2C19, 2C8 and 2B6; 15.9 μM vs 1A2).^13^ *In vivo*, PAM **6** displayed high clearance (CL_p_ = 61 mL/min/kg), yet high CNS penetration (rat *K*_p_ = 3.7). Additionally, **6** showed low kinetic solubility (2.0 μM at pH 2.2 and <1.5 μM at pH 6.8). Thus, the HTS campaign identified the first selective (vs mGlu_2_), potent, and CNS-penetrant mGlu_3_ PAM (**6**); however, the team needed to improve the DMPK profile (lower CL_p_ and increase unbound fraction in plasma/brain) to enable robust target validation studies in preclinical cognition models.

**Figure 2:**
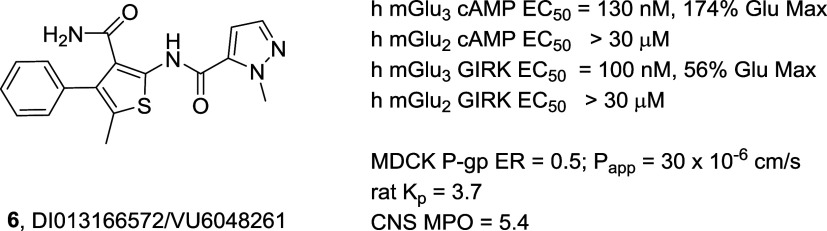
Structure and select pharmacology and DMPK data for the first selective and CNS-penetrant mGlu_3_ PAM DI01316572/VU6048261 (**6**).

### Chemical Lead Optimization

We envisioned a multipronged approach for the chemical optimization of PAM **6** (Figure 3). Molecular modeling and energy calculations suggested (Figure 3A) a preferred planar conformation (more than 10 kcal/mol versus the next lowest energy conformation) for the thiophene amide moiety, and efforts were applied to various cyclization strategies (Figure 3B) to improve disposition. In parallel, a traditional multidimensional optimization effort was also pursued to evaluate the four regions highlighted in Figure 3C. Here, substituents on the phenyl ring, as well as heterocycles, were explored as alternatives for the primary carboxamide and alternate heteroaryl amides. Importantly, the team hoped to find bioisosteric replacements for the potentially metabolically liable thiophene core^15^ en route to a clinical candidate; however, retention of the thiophene in an *in vivo* tool compound was acceptable.

**Figure 3:**
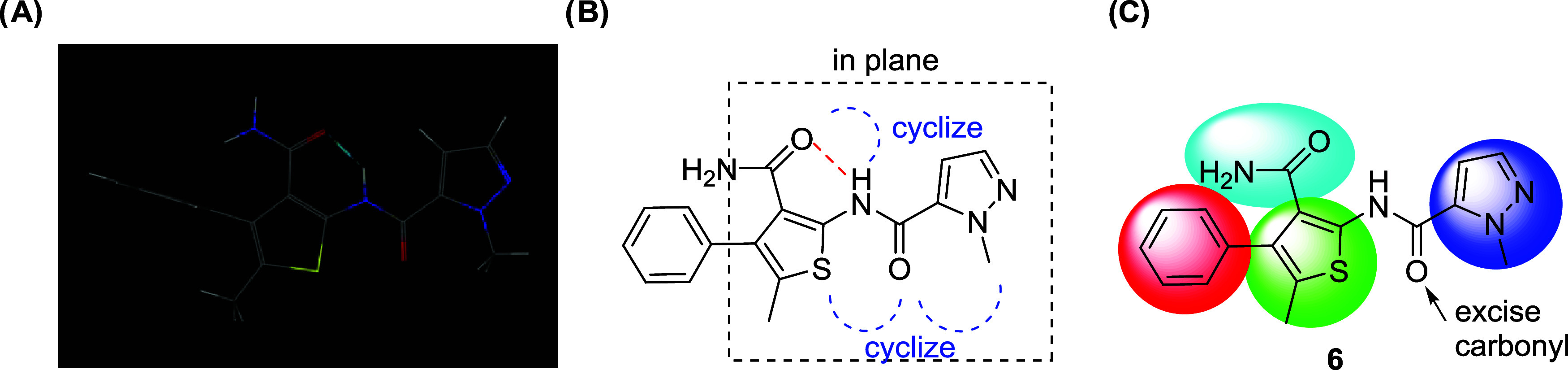
Chemical lead optimization plan. (A) Energy minimization elucidates a preferred planar conformation >10 kcal/mol versus next favorable conformation. (B) Multiple cyclization strategies to take advantage of the planar conformation. (C) Standard multidimensional optimization of four regions of PAM **6**.

Despite significant effort and clever tactics, all attempts to cyclize PAM **6** led to inactive compounds, as did efforts to replace the core tetra-substituted thiophene. Moreover, the primary carboxamide proved to be a key pharmacophore for mGlu_3_ PAM activity. However, tractable SAR was achieved by exploring alternative amide moieties, excising the amide carbonyl, and modifications of the phenyl ring. To explore these regions of PAM **6**, we developed a four-step synthetic sequence to analogs rapidly (Scheme 1).^13^ Starting from various substituted commercial propiophenones **7**, a Gewald reaction readily provides the desired tetra-substituted thiophene cores **8** in yields ranging from 44 to 66%. Partial hydrolysis of the nitrile in neat sulfuric acid affords the primary carboxamides **9** in good yield (66–94%). Coupling of the deactivated amine moiety proved problematic, but ultimately, PyClU-mediated amide couplings under microwave irradiation delivered the desired analogs **10** in acceptable yields (22–70%).

**Scheme 1:**
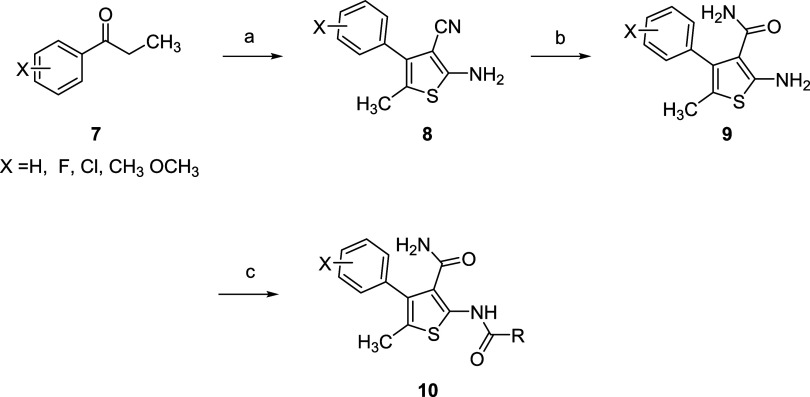
Synthesis of Tetra-Substituted Thiophene Amide Derivatives **10** ^a^

a Reagents and conditions. (a) Sulfur (1.5 equiv), l-proline (15 mol %), malononitrile (1.2 equiv) DMF, 60 °C, 16 h, 44–66%; (b) H_2_SO_4_ neat, 70 °C, 3 h, 66–94%; (c) PyCLU, DMF, microwave, 15 min, 22–70%.

As shown in Table 1, resynthesis of PAM **6** proved to be equivalent to the HTS sample (EC_50_ = 102 nM, 66%), and a wide range of 5- and 6-membered heterocycles, as well as saturated ring systems, were tolerated. An ∼3-fold difference in mGlu_3_ PAM potency was noted between regioisomeric oxadiazoles **10b** (EC_50_ = 304 nM, 59% Glu Max) and **10c** (EC_50_ = 108 nM, 82% Glu Max). A slight improvement in potency, and a larger impact on efficacy, was observed upon changing the *N*-Me pyrazole of **6** from the 5-position to the 4-position **10d** (EC_50_ = 80 nM, 95% Glu Max). Saturated 6-membered ring analogs (**10e**-**g**) lost potency relative to **6**, while pyridine (**10h**) and pyridazine (**10i**) were equipotent. *In vitro* DMPK profiling of **10d** indicated that the optimization was moving in the right direction. PAM **10d** displayed significantly improved fraction unbound (h,r *f*_u_ = 0.022, 0.047) relative to **6**, and comparable predicted hepatic clearance (CL_hep_ (h,r)) = 18 and 40 mL/min/kg, respectively.^13^ Based on the improved efficacy and unbound fraction of **10d**, we elected to hold that amide moiety constant and explore alternate substituents on the phenyl ring, providing analogs **11** (Table 2). As unbound fraction and clearance were areas for improvement, we began measuring these parameters for all analogs **11**.

**Table 1:**
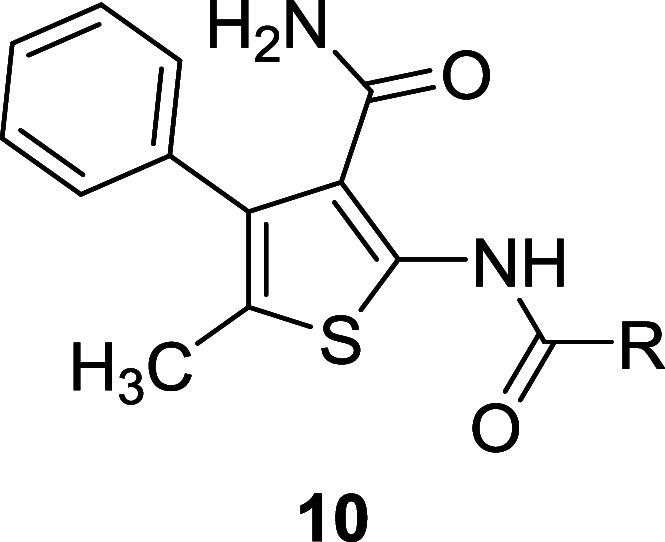

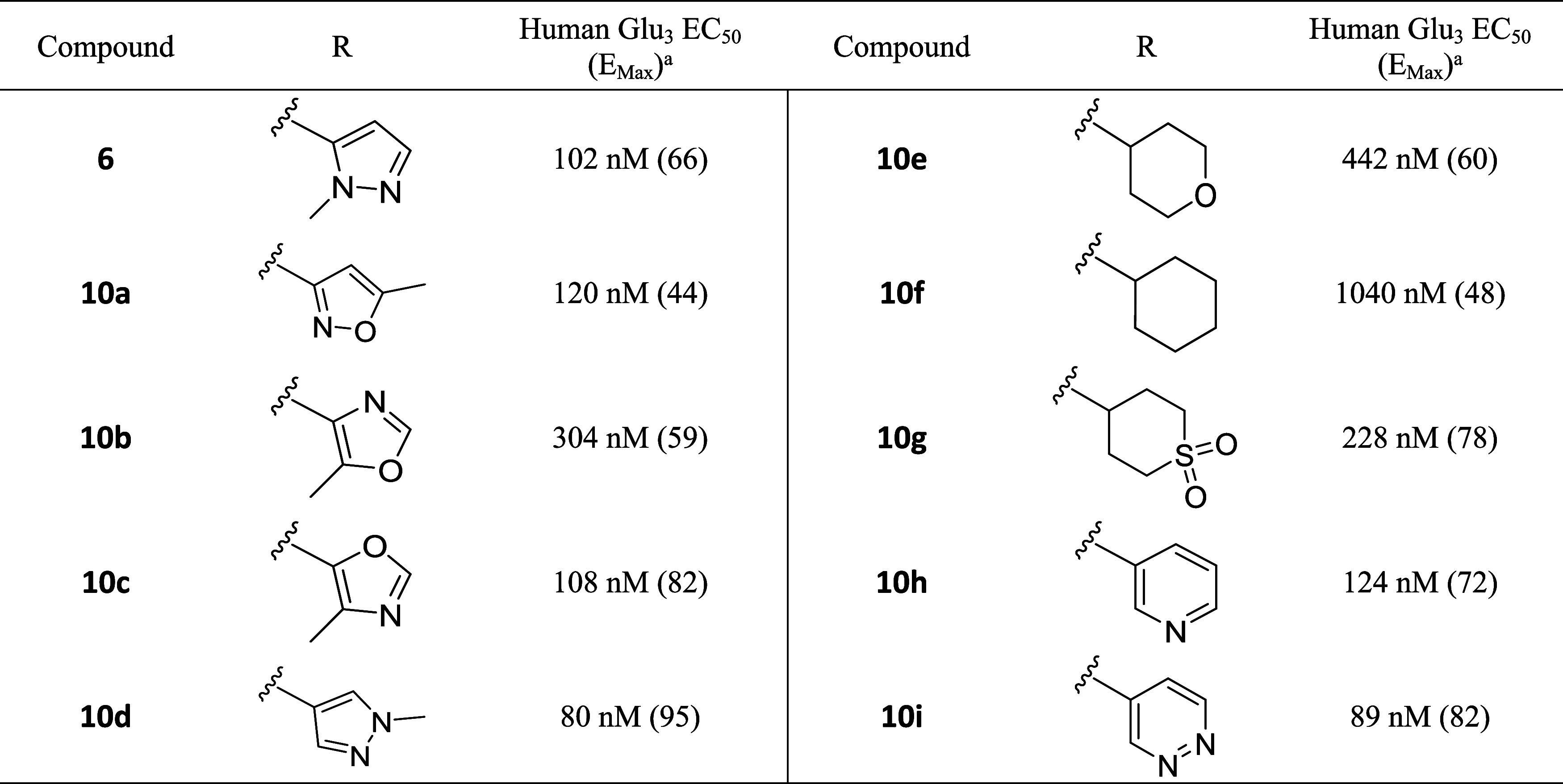
Structures and mGlu_3_ PAM Activity of Analogs **10**

a Human mGlu_3_-GIRK assay with an ∼EC_20_ of glutamate. Data represent at least a single experiment performed in triplicate.

**Table 2:**
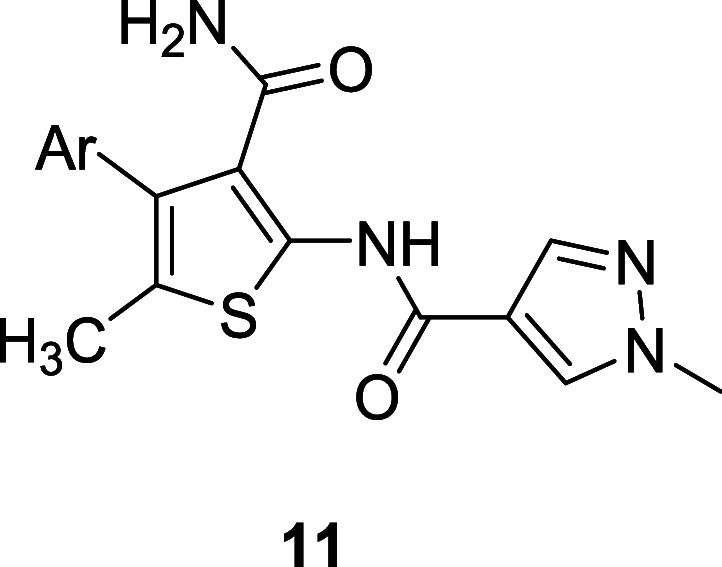

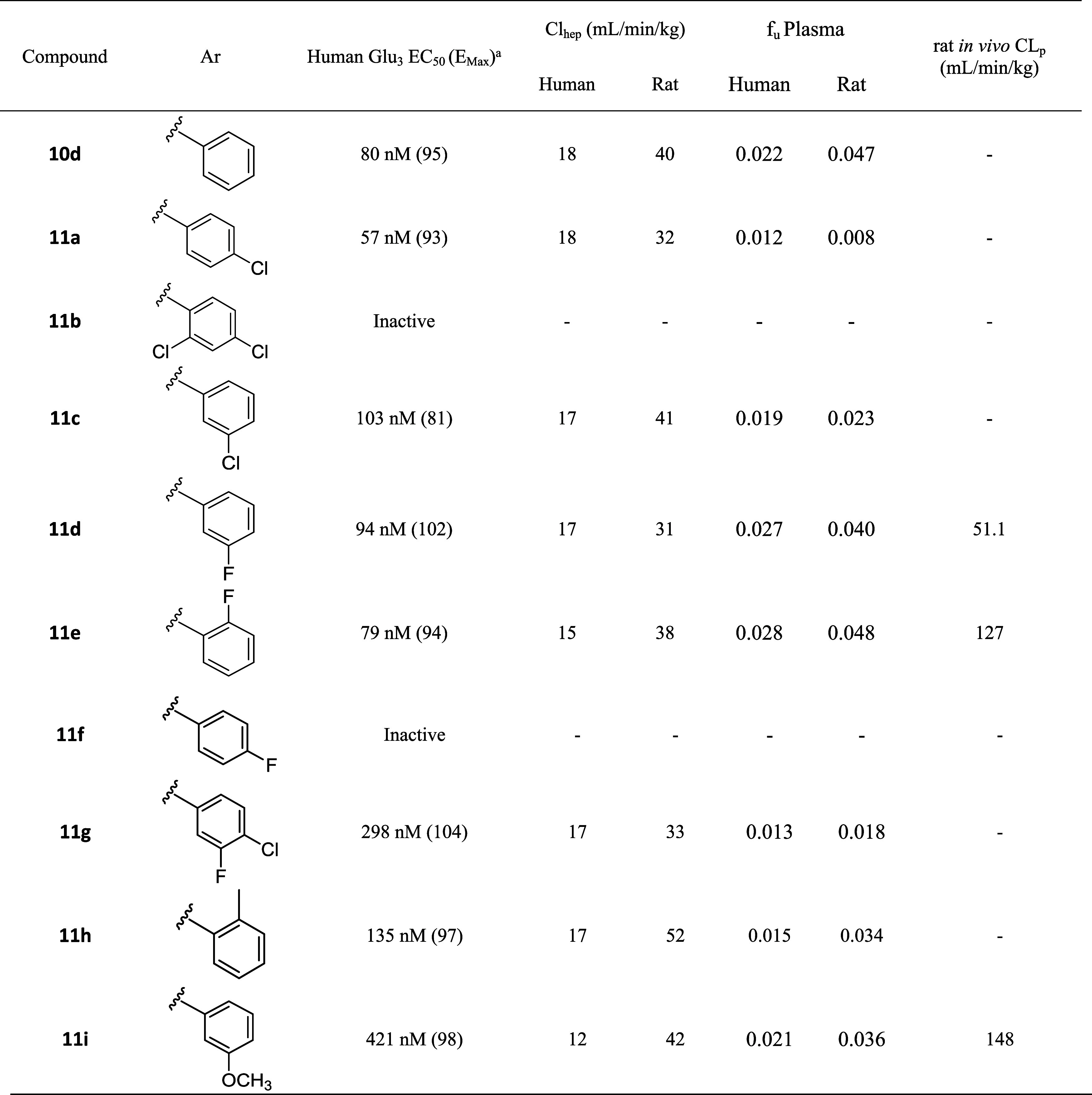
Structures, mGlu_3_ PAM Activity, and *In Vitro* DMPK Data of Analogs **11**

a Human mGlu_3_-GIRK assay with an ∼EC_20_ of glutamate. Data represent at least a single experiment performed in triplicate.

A 4-chlorophenyl analogue **11a** showed slightly enhanced functional potency (EC_50_ = 57 nM, 93%) but at the cost of unbound fraction. A 2,4-dichlorophenyl derivative **11b** was inactive, whereas a 3-chlorophenyl variant **11c** was equivalent to **10d**. Interestingly, both 2-fluorophenyl **11d** and 3-fluorophenyl **11e** congeners were essentially equipotent and with comparable unbound fractions to **10d**; however, neither had a significant impact on human or rat predicted hepatic clearance. Replacement of the 4-chloro substituent in analogue **11a** with a fluorine atom was not tractable, as the 4-fluorophenyl analogue **11f** was inactive. Electron-donating moieties such as **11h** and **11i** resulted in lost mGlu_3_ PAM potency and similarly had no positive effect on predicted hepatic clearance or unbound fractions. However, an *in vitro*/*in vivo* disconnect with regard to clearance was observed in rat IV PK PBL cassettes. Select analogues **11** showed high to superhepatic CL_p_ values, suggesting we excise the carbonyl and replace the amide linked with a heterobiaryl amine linkage.

The synthesis of the requisite heterobiaryl amines **12** is shown in Scheme 2. Starting with amine intermediate **9** (where X is either H or F), a Buchwald coupling facilitated with BrettPhos as a palladium ligand in dioxane at 100 °C, afforded analogs **12** in yields ranging from 30 to 89%. Here, we focused (Table 3) on 6-membered heterocyclic rings as well as 5,6- and 6,6-fused heterobicyclic cores. In general, the heterobicyclic cores **12a,b** and **12i,j** were weak (EC_50_s ∼ 1 μM) to inactive. Incorporation of monocyclic aza-ring systems, such as regioisomeric pyrimidines **12c,d,e,f,k,l** provided analogs with reasonable potency, but lower Glu Max values (40–57%). Of these, we focused on **12c** (EC_50_ = 288 nM, 50% Glu Max), and collected tier 1 *in vitro* DMPK data as well as rat IV cassette and PBL PK. Gratifyingly, **12c** displayed a significant improvement in terms of fraction unbound (h,r *f*_u_ = 0.087, 0.14) relative to **6**, and improved predicted hepatic clearance (CL_hep_ (h,r) = 14 and 49 mL/min/kg, respectively). Brain homogenate binding also followed the trend (rat BHB *f*_u_ = 0.087). *In vivo*, **12c** was moderately cleared in rat (CL_p_ = 48.4 mL/min/kg), highlighting a robust *in vitro*/*in vivo* correlation (IVIVC), but short half-life (*t*_1/2_ = 0.3 h). In addition, **12c** was CNS penetrant in rat (*K*_p_ = 1.2; *K*_p,uu_ = 0.74); however, this PAM possessed an unacceptable CYP_450_ profile (IC_50_s: 3A4 (2.9 μM), 2D6 (18.7 μM), 2C9 (>30 μM), 1A2 (0.1 μM)).^13^ Based on these data, the des-fluoro congener **12d** (EC_50_ = 423 nM, 55% Glu Max) was also evaluated. Unbound fraction was again improved in both plasma (h,r *f*_u_ = 0.10, 0.12) and brain (rat BHB *f*_u_ = 0.06), as well as *in vivo* rat PK parameters (CL_p_ = 36.5 mL/min/kg, *t*_1/2_ = 0.3 h, *V*_ss_ = 0.87 L/kg). For **12d**, CNS penetration in rat remained high (*K*_p_ = 0.75, *K*_p,uu_ = 0.37) and the CYP_450_ profile remained poor (IC_50_s: 3A4 (3.1 μM), 2D6 (>30 μM), 2C9 (>30 μM), 1A2 (0.1 μM)), yet this compound exhibited improved kinetic solubility (82 μM at pH 2.2 and 89 μM at pH 6.8). Thus, SAR was trending toward improved mGlu_3_ PAMs, but parameters for improvement were *in vivo* rat PK properties and CYP_450_ profiles.

**Scheme 2:**
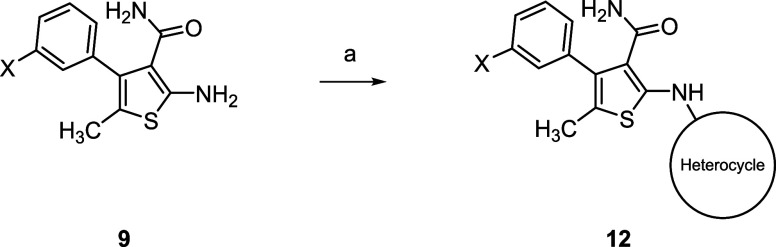
Synthesis of Heterobiaryl Amine Derivatives **12** ^a^

a Reagents and conditions. (a) K_2_CO_3_ (3 equiv). BrettPhos (40 mol %), Pd_2_(dba)_3_ (10 mol %), heteroaryl bromide (1.5 equiv), 1,4-dioxane, 100 °C, 3 h, 30–89%.

**Table 3:**
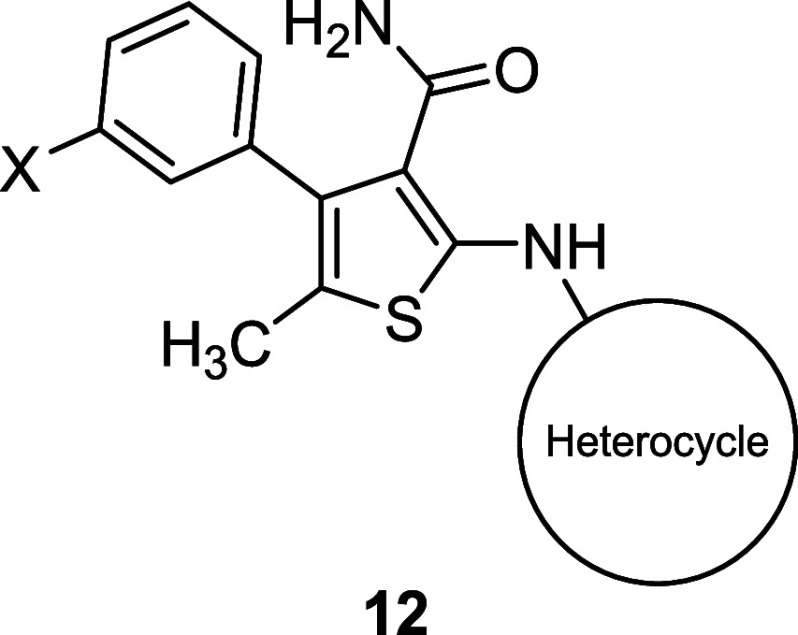

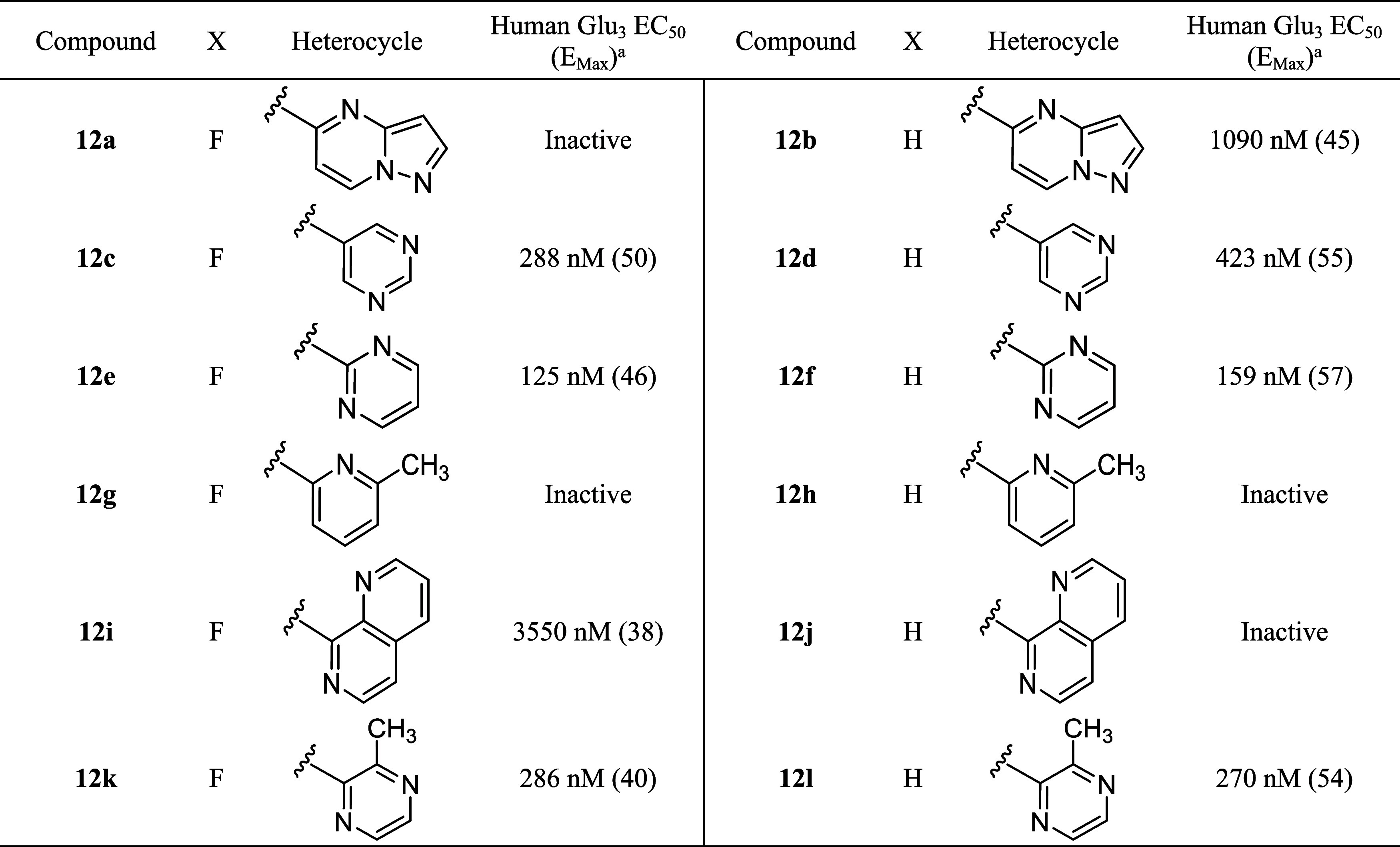
Structures and mGlu_3_ PAM Activity of Analogs **12**

a Human mGlu_3_-GIRK assay with an ∼EC_20_ of glutamate. Data represent at least a single experiment performed in triplicate.

We focused on the incorporation of various substituents to the 2-position of the pyrimidine ring of **12c,d** as in derivatives **13** (Table 4) and tracked mGlu_3_ PAM potency SAR along with predicted hepatic clearance for both human and rat. This exercise led to a large number of active mGlu_3_ PAMs, and some displayed improvements in predicted hepatic clearance (%Q_H_ from ∼75% to below 50%). Of these, the tertiary alcohol **13l** (VU6053371/BI03738809) stood out as an exceptional mGlu_3_ PAM (EC_50_ = 113 nM, 61% Glu Max) with low predicted hepatic clearance (CL_hep_ = 4 and 27 mL/min/kg for human and rat, respectively). With the need to quickly provide proof-of-concept and target validation for mGlu_3_ PAMs in preclinical rodent cognition models, efforts focused on deeper profiling of **13l** (VU6053371/BI03738809).

**Table 4:**
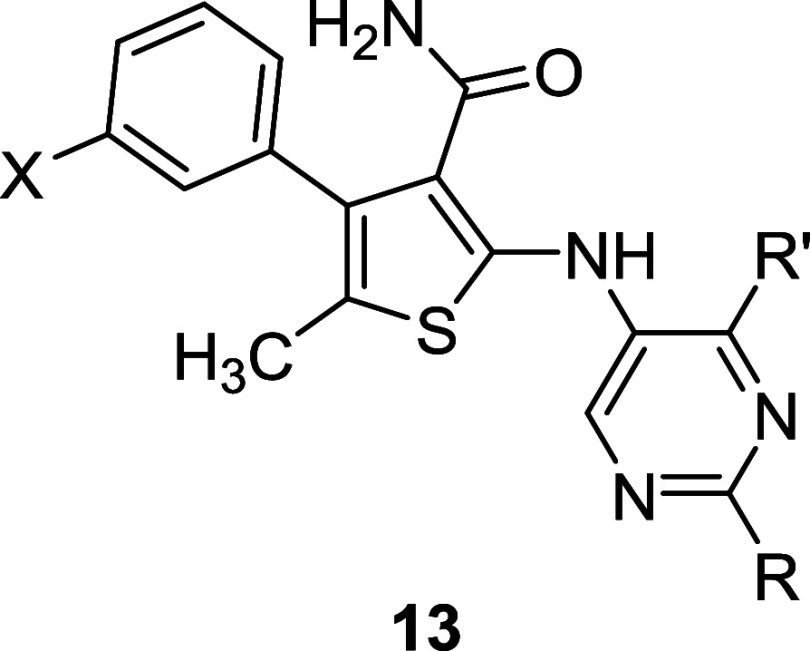

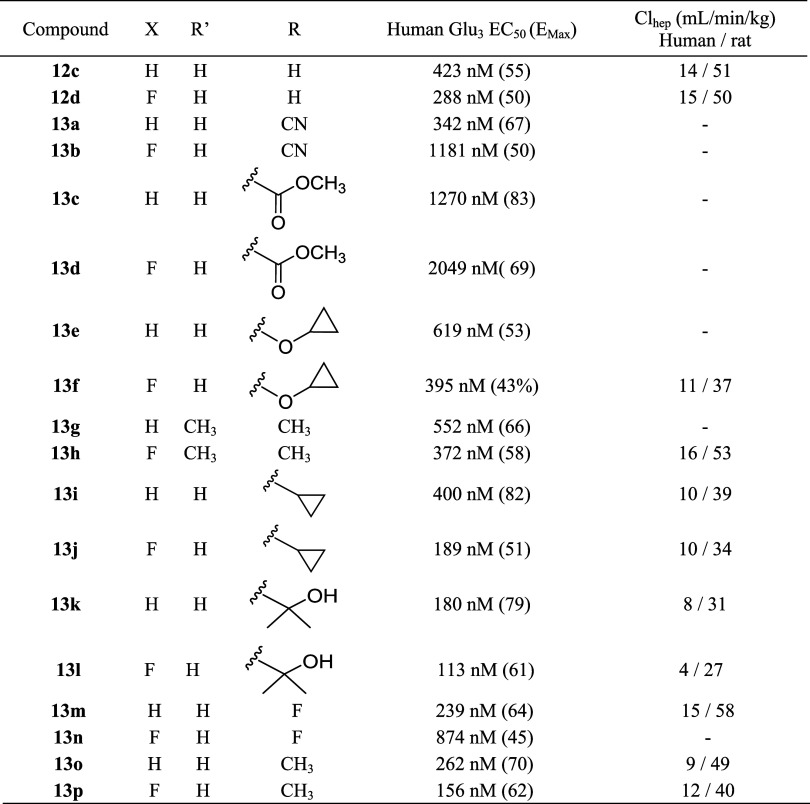
Structures and mGlu_3_ PAM Activity of 2-Substituted Pyrimidine Congeners **13** ^a^

a Human mGlu_3_-GIRK assay with an ∼EC_20_ of glutamate. Data represent at least a single experiment performed in triplicate.

### Pharmacological and DMPK Profiling

**13l** (Table 5) proved to be an equipotent mGlu_3_ PAM for both human (EC_50_ = 113 nM, 61% Glu Max) and rat (EC_50_ = 112 nM, 58% Glu Max) and, importantly, inactive (EC_50_ > 30 μM) for both human and rat mGlu_2_. In the human mGlu_3_ CHO cAMP assay, good agreement was shown (EC_50_ = 90 nM, 41% Glu Max). PAM **13l** was also selective versus the other mGlu receptors (EC_50_ > 10 μM), and thus **13l** is a first-in-class (FIC) selective mGlu_3_ PAM. Unbound fraction was exceptional in both plasma (h,r *f*_u_ = 0.11, 0.18) and brain (rat BHB *f*_u_ = 0.047), as well as predicted hepatic clearance (CL_hep_ (h,r) = 4 and 27 mL/min/kg, respectively). Unlike other analogs in this series, **13l** displayed a good CYP450 profile (IC_50_s: 3A4 (>30 μM), 2D6 (>30 μM), 2C9 (16 μM), 1A2 (26 μM)), even against 1A2. There was no time-dependent CYP_450_ inhibition at 3A4, mini-AMES (with and without S9) was negative, and a functional EP hERG assay yielded no CV/QT concern (IC_50_ > 30 μM). Solubility is the bane of allosteric modulator drug discovery, as the allosteric sites are often deep in the lipophilic transmembrane domain. Encouragingly, **13l** possessed exceptional aqueous solubility (69 μM at pH 6.8; 83 μM at pH 2.2), and high solubility in both FaSSIF (75 μg/mL) and FaSSGF (183 μg/mL). Finally, predicted human CNS penetration in an MDCK-MDR1 assay for **13l** was high (ER = 0.5; *P*_app_ = 82 × 10^–6^ cm/s). In a Eurofin SafetyScreen 44, the most significant displacement of radioligands was at the human dopamine transporter (40% @ 10 μM), COX2 (65% @ 10 μM), and Lck kinase (54% @ 10 μM).^13^ Follow-up radioligand binding assays for DAT produced a binding IC_50_ of 7.5 μM and a *K*_i_ of 6.0 μM (affording at least an ∼50-fold selectivity window),^13^ and thus the team felt **13l** could still serve as a putative *in vivo* proof-of-concept compound for target validation.

**Table 5:**
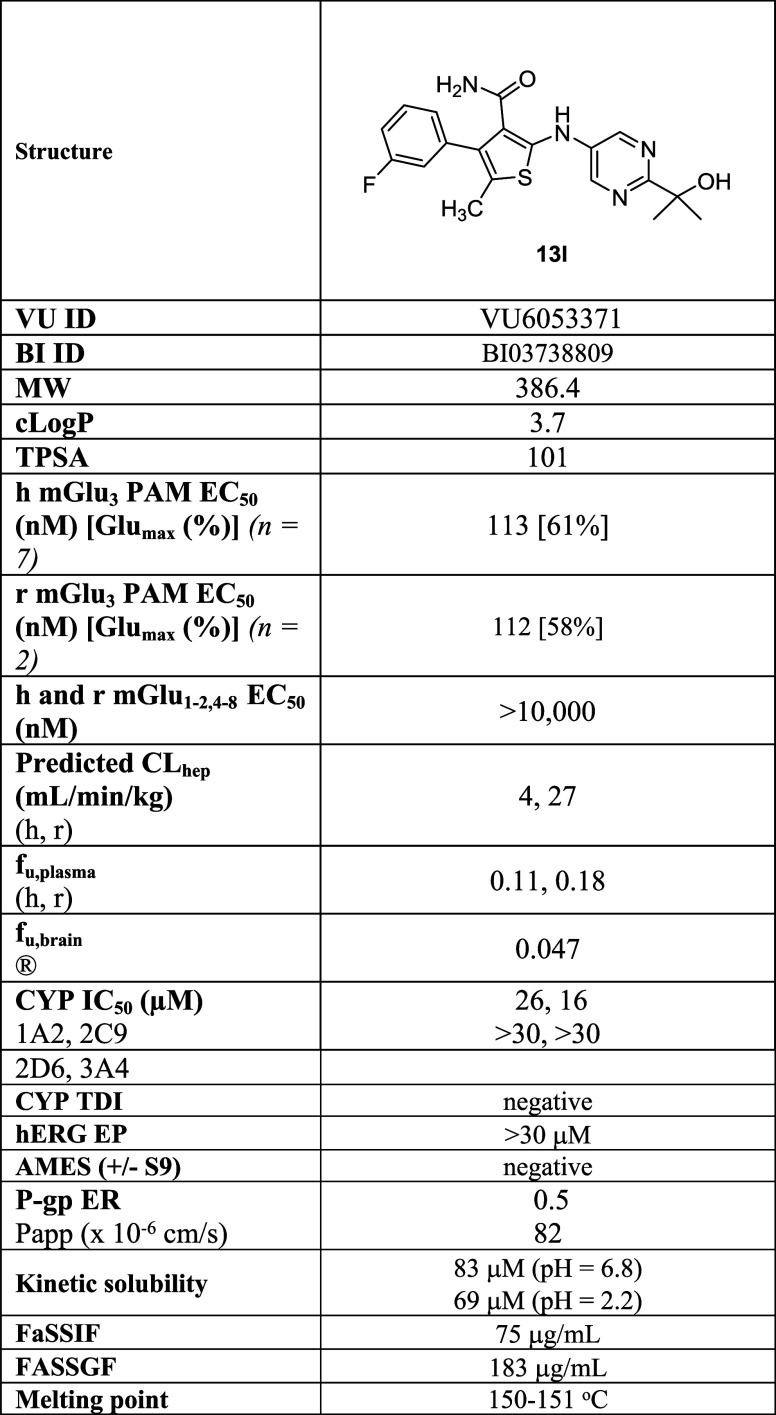
Pharmacology and *In Vitro* DMPK Profile of **13l**

*In vivo*, PAM **13l** displayed a very attractive profile. In a rat IV/PO PK crossover study, PAM **13l** exhibited low clearance (CL_p_ = 10 mL/min/kg), as well as good half-life (*t*_1/2_ = 2.6 h) and volume (*V*_ss_ = 1.6 L/kg). In the PO arm, *T*_max_ was achieved at 1 h with high *C*_max_ (2.75 μM) and 82% oral bioavailability. In a rat IV PBL cassette (0.2 mg/kg, 15 min time point), **13l** demonstrated a *K*_p_ of 8.4 and a *K*_p,uu_ of 2.9, and in a discrete IV PBL study (3 mg/kg, 15 min time point), **13l** *K*_p_ of 1.91 and a *K*_p,uu_ of 0.49. In a follow-up oral PBL study (3 mg/kg, 1 h time point), **13l** showed a *K*_p_ of 2.24 and a *K*_p,uu_ of 0.57. Combined, the pharmacological, *in vitro*, and *in vivo* DMPK profiles supported **13l** as a potent, selective, and CNS penetrant mGlu_3_ PAM tool compound.^13^

### *In Vivo* Behavior and Proof-of-Concept Studies

Based on the presumed mechanism of pro-cognitive efficacy of an mGlu_3_ PAM, the team evaluated **13l** in rat novel object recognition (NOR)^16^ (Figure 4). Pilot oral PK at 3 mg/kg PO was evaluated to select doses for the NOR study. At 3 mg/kg PO in rat, total plasma exposure reached 2.3 μM (430 nM free) with total brain exposure of 5.2 μM (246 nM free) for a *K*_p_ of 2.24 and a *K*_p,uu_ of 0.57 for an average of 2.2-fold above the rat mGlu_3_ PAM EC_50_ (112 nM). Thus, the dose range for this study was conducted at 1, 3, and 10 mg/kg PO. As illustrated in Figure 4, mGlu_3_ PAM **13l** dose-dependently enhanced recognition memory in rats with a minimum effective dose (MED) of 3 mg/kg PO and a near maximum response at 10 mg/kg PO. Rat satellite PK studies at 1, 3, and 10 mg/kg PO (male, *n* = 3) established a clear pharmacokinetic/pharmacodynamic (PK/PD) relationship for selective mGlu_3_ potentiation in the NOR paradigm. At 1 mg/kg PO, free brain levels were 36.2 nM (∼0.3-fold the rat mGlu_3_ PAM EC_50_), with no significant effect observed versus vehicle. At 3 mg/kg PO (the MED), unbound brain levels were 151 nM (∼1.35-fold the rat mGlu_3_ PAM EC_50_), and maximal efficacy was noted at 10 mg/kg PO (free brain = 385 nM, ∼3.43-fold the rat mGlu_3_ PAM EC_50_).^13^ Thus, unbound brain exposures at, or above, the rat mGlu_3_ PAM EC_50_ afford a robust PK/PD relationship for efficacy in the NOR paradigm. These data represent the first example of pro-cognitive efficacy by selective potentiation of mGlu_3_, and further support the therapeutic utility of mGlu_3_ PAMs in schizophrenia,^4–10^ Alzheimer’s disease,^17^ and other forms of dementia and cognitive impairment.

**Figure 4:**
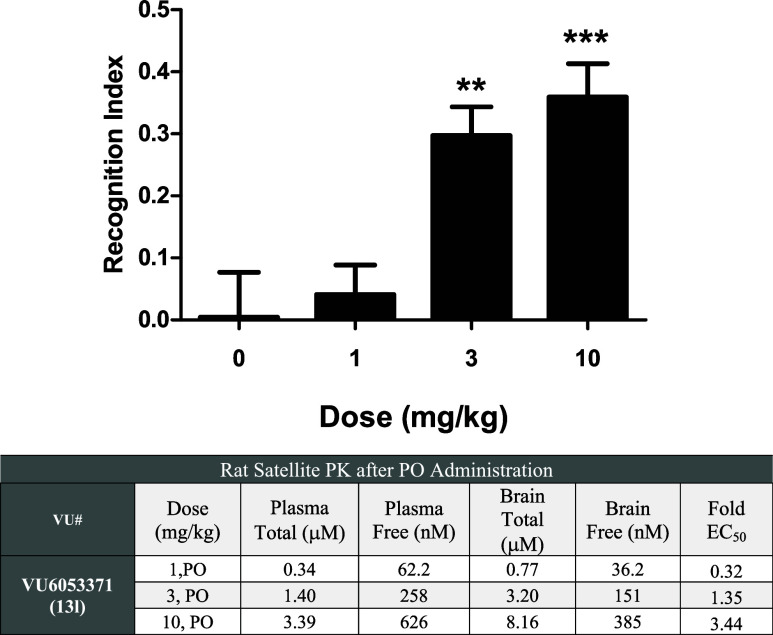
Novel object recognition (NOR) test in rats with VU6053371/BI03738809 (**13l**). PAM **13l** dose-dependently enhanced recognition memory in rats. Pretreatment with 1, 3, and 10 mg/kg **13l** (PO 0.5% natrosol/0.015% Tween 80 in water, 30 min) prior to exposure to identical objects significantly enhanced recognition memory assessed 24 h later. *N* = 13–16/group of male Sprague–Dawley rats. **p < 0.01; ***p < 0.001 one-way ANOVA, Dunnett post hoc test.

### Electrophysiology

Genetic studies have indicated that allelic variations in *GRM3*, the human gene encoding mGlu_3_, can impact prefrontal cortex (PFC)-mediated cognitive functions.^6,18^ We recently demonstrated that NMDA receptor-independent long-term depression (LTD) with a postsynaptic component is induced by pharmacological activation of group II mGlu receptors in mPFC. To delineate the roles of the mGlu_2_ and mGlu_3_ receptor subtypes, these early studies employed mGlu_2_ and mGlu_3_ knockout mice and the use of mGlu_2_ NAMs and mGlu_3_ NAMs in combination with a group II agonist; these studies indicated that activation of mGlu_3_ is responsible for enhancing PFC Ltd.^10,11^ With the selective mGlu_3_ PAM **13l**, we are now poised to recapitulate these early findings directly. Here, PAM **13l** (Figure 5) potentiates group II agonist LY379268-induced LTD in mouse PFC slices, further confirming earlier work with KO mice and selective NAMs.^13^ Taken together, the behavioral and electrophysiology data validate the mGlu_3_ PAM mechanism as a novel therapeutic strategy for enhancing cognition and prefrontal function in patients. In addition, the utility of **13l** (VU6053371/BI03738809) as a first-in-class mGlu_3_ PAM tool compound is clear; however, the potential of **13l** as a putative clinical candidate is far less clear.

**Figure 5:**
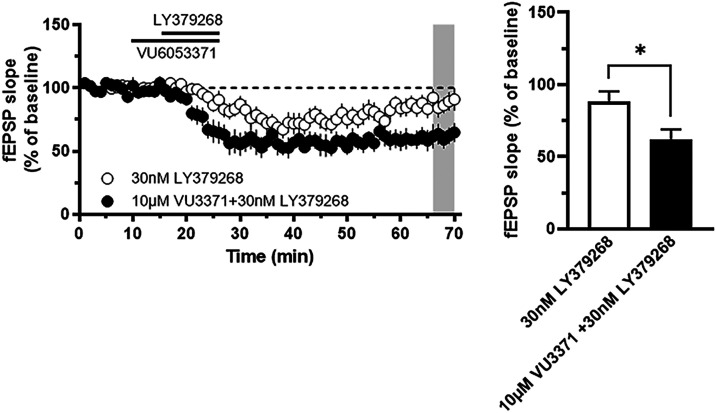
mGlu_3_ activation is required for the induction of LTD by a group II agonist, LY379268. A submaximal concentration of LY379268 induces LTD in mPFC slices, and this effect is potentiated by the selective mGlu_3_ PAM **13l** (VU6053371/BI03738809). *N* = 8–10 slices from 5 to 8 mice per group. Averaged data from the last 5 min, as indicated by the gray bar in A, were compared. *p = 0.016, two-tailed *t* test.

### Derisking for Candidacy

The project team was concerned about the potential, and well-established, metabolic liability of the thiophene moiety in **13l**,^15^ and while a valuable rodent tool compound to probe selective mGlu_3_ activation, it may not have the stamina to advance into IND-enabling studies. To derisk these concerns, we performed multispecies hepatocyte metabolism identification (Met ID) and glutathione (GSH) conjugate screening in human liver microsomes. Across mouse, rat, dog, minipig, monkey, and human (Figure 6), the parent **13l** was the predominant species after incubation in hepatocytes (mouse, 77.4%; rat, 87.8%; dog, 87.1%; minipig, 75.6%; monkey, 60.9%; human, 91.3%).^13^ The most abundant metabolite in all species, except humans, was M402a, the result of oxidation of one of the methyl groups of the tertiary hydroxyl moiety, affording a diol (Figure 7). In humans, the most abundant metabolite was a glucuronide M562, which was also present in all higher species except rodent. Otherwise, both human metabolites were covered in higher species, although they all afforded several unique metabolites not produced in humans. Rodents afforded coverage only for the oxidative metabolite M402.

**Figure 6:**
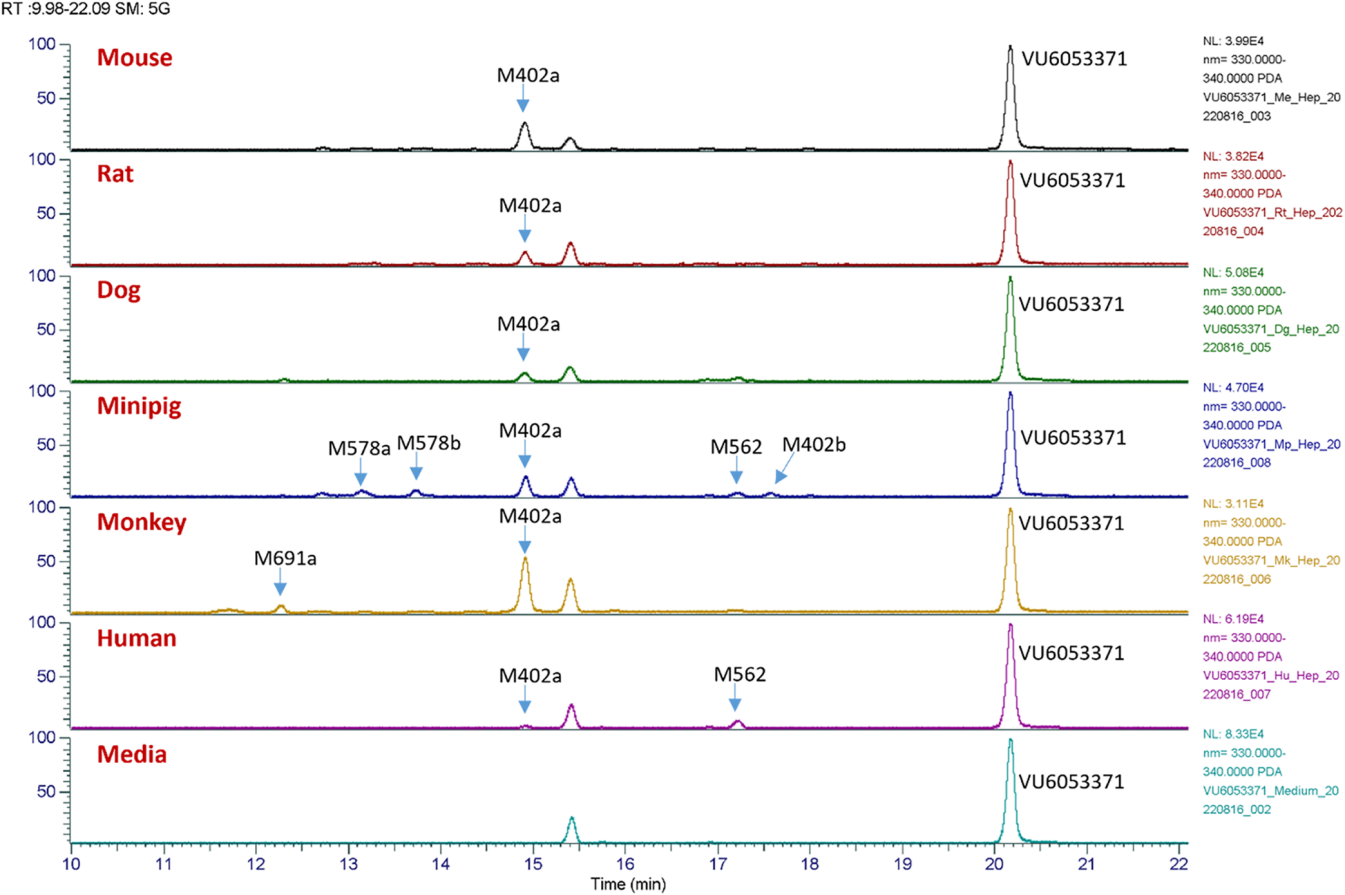
Metabolism of VU6053371 (**13l**) in mouse, rat, minipig, dog, monkey, and human hepatocytes, with the most abundant metabolite, M402A, an oxidation product, and a glutathione conjugate, M562, that was not represented across species.

**Figure 7:**
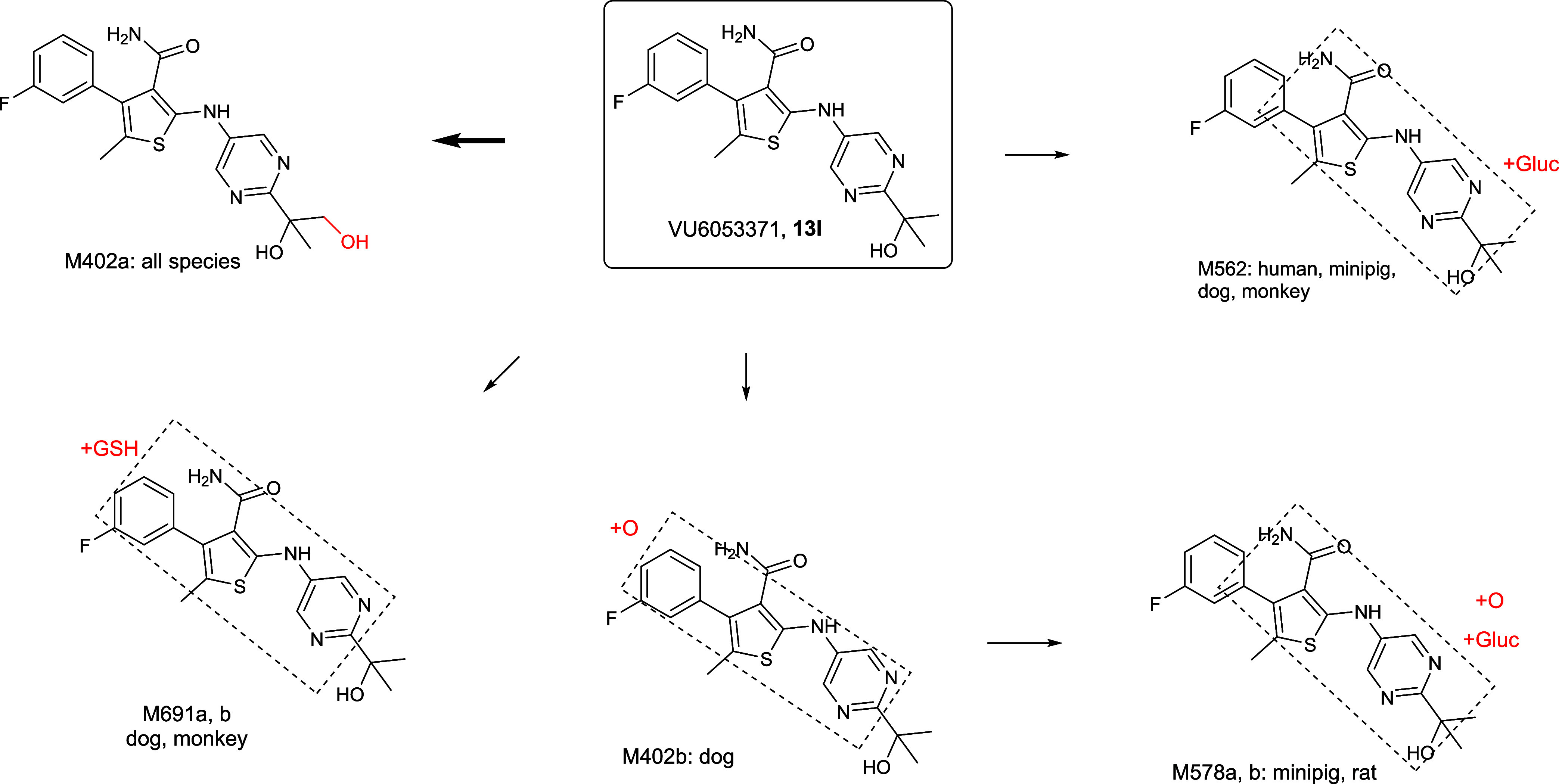
Structures of putative metabolites of VU6053371 (**13l**), including the major oxidative metabolite, diol M402a and glucuronide conjugate M562.

An additional derisking step was to assess reactive intermediate formation by assessing GSH trapping in human liver microsome incubations, as the thiophene core, a putative electrophilic toxicophore, was a concern.^15^ One GSH conjugate was identified (M691) after **13l** was incubated in fortified microsomes with human liver cytosol and GSH (Figure 8). Moreover, the formation of M691 was NADPH-dependent. The GSH adduct had a mass of 692.1967 (parent mw is 387.1287), leading to a proposal of the GSH adduct composition as parent + GSH-2H, and **13l** formed 5.9% of this adduct.^13^

**Figure 8:**
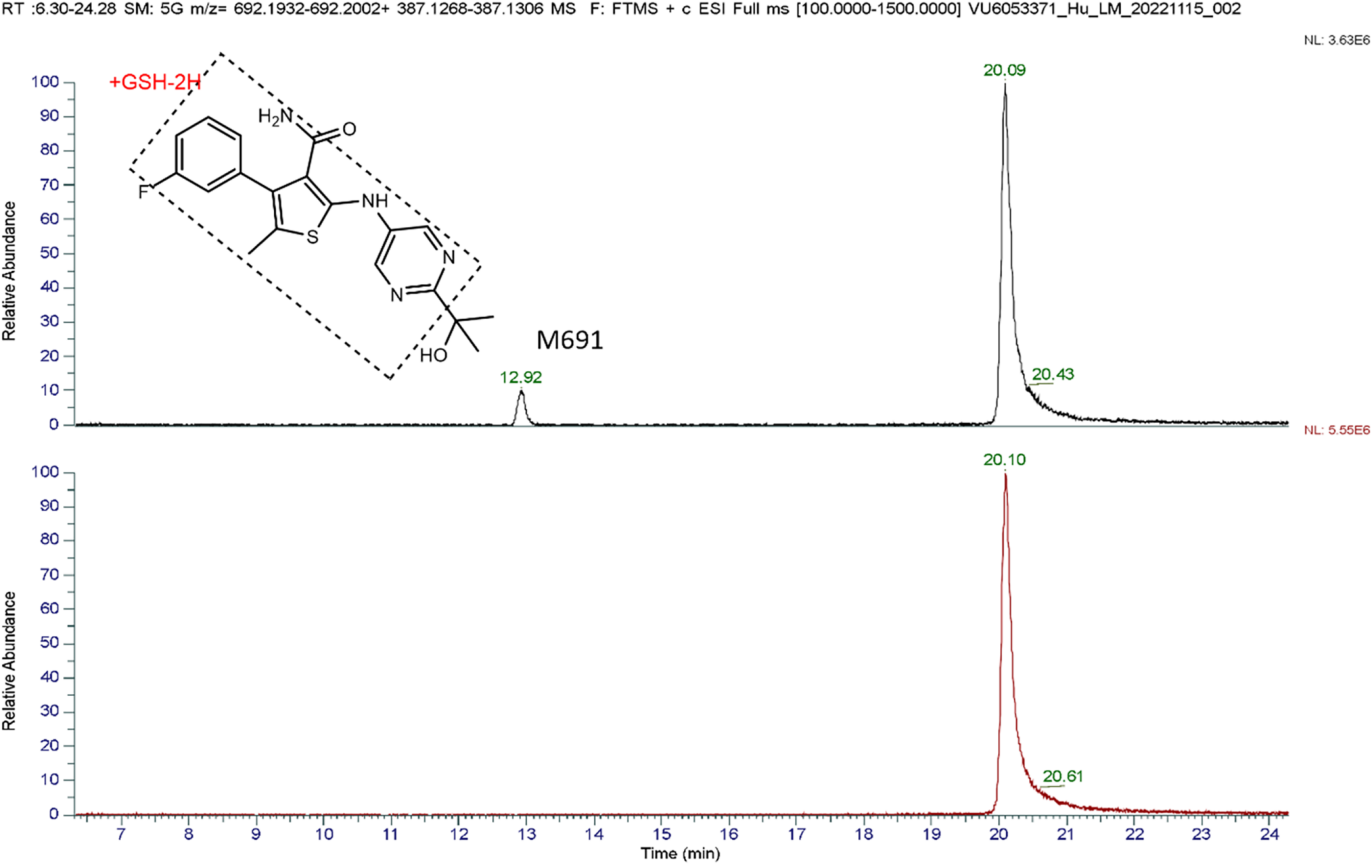
Detection of GSH adducts in liver microsome incubation enriched with GSH in the presence of **13l**. Human liver microsome incubations were carried out at 10 μM concentration of the test article supplemented with NADPH (2 mM) and stable isotope-labeled GSH (10 mM, GSH:^3^GSH = 1:1). A protein concentration of 1.0 mg/mL was utilized with a total incubation volume of 500 μL. Incubations were initiated by the addition of NADPH (2 mM). After 1 h, the sample was quenched with an equal volume of acetonitrile, vortexed, centrifuged for 5 min at 13,000 rpm, and the supernatant (170 μL) was transferred to a 96-well plate and partially dried under nitrogen to 120 μL. The LC/MS full scan data was processed manually, assisted by Compound Discoverer, to identify the potential GSH conjugates.

### Explorative Toxicological Profiling in Rats Results: BI03738809 Exploratory 1-Week Oral (Gavage) Toxicity Study (Non-GLP) in Male Rats

To assess the toxicological profile of **131**, a nine-day oral toxicity study of BI03738809 (**13l**) was conducted in male rats, which received 0, 30, 100, or 300 mg/kg twice daily (8–16 h apart). Doses were chosen based on rat pharmacokinetic data and expected mGlu_3_ target coverage. Toxicokinetic analysis demonstrated systemic drug exposure in all animals treated with **13l** (Table 6). On Day 1, the *C*_max_ values following the initial morning dose correspond to estimated mGlu_3_ fold EC_50_s of 21, 47, and 61 times, based on calculated free brain exposures.

**Table 6:** VU6053371/BI03738809 (PAM **13l**) Plasma Exposure Achieved in Exploratory (Non-GLP) 1-Week Toxicity Study after Twice Daily Dosing to Male Rats

	*C*_max_ [μM]			AUC_0–24_ [μM·h]	
dose group [mg/kg twice daily]	after first dose only	Day 1	Day 7	Day 1	Day 7
30	19.7	23.8	15.0	180	94.4
100	44.0	58.7	36.8	826	411
300	56.8	^a^	^a^	^a^	^a^

a Animals in the high-dose group were prematurely sacrificed at 7–8 h post-morning dose on Day 1.

Due to the rapid onset of striking clinical signs (e.g., decreased motor activity, closed bilateral palpebral fissure, reduced response to stimuli, swaying gait, rough fur, apathy, cold to touch, diminished motor tone and lying position) in combination with reduced body temperature, all animals at 300 mg/kg (5/5 main study animals and 3/3 satellite animals) were prematurely sacrificed on Day 1 approximately 7–8 h after the first dose. Animals receiving the 100 mg/kg/dose showed several similar clinical signs between Days 1 and 3, approximately 0.5–3 h postdose. These included decreased motor activity, closed bilateral palpebral fissure, reduced response to stimuli, swaying gait, and rough fur, which in combination were considered adverse. However, the duration of clinical signs resolved under treatment from Day 4, likely due to reduced systemic drug exposure in plasma from Day 1 to Day 7 (about a factor 2 lower AUC_0–24_ on Day 7 as compared to Day 1; see Table 6). No abnormal clinical signs were noted at the low dose of 30 mg/kg/dose.

At the end of dosing, clinical pathology showed mildly to moderately reduced reticulocyte counts at 30 and 100 mg/kg/dose on day 8 versus controls. Animals given a single dose of 300 mg/kg and were prematurely sacrificed had moderately lower white blood cell, lymphocyte, and monocyte counts compared to controls.

Pathology found diffuse decreased bone marrow cellularity in all males at 100 mg/kg/dose, absent in controls, and attributed to **13l** after 8 days of treatment. All high-dose males exhibited mild multifocal inflammatory foci in the myocardium, not limited to a specific heart region. A similar lesion was seen in one control animal. Myocardial inflammatory foci can occur spontaneously in rats; however, given the consistent presence in all high-dose animals, a relationship to the **13l** is likely. CAB staining showed myocardial fiber injury only where inflammation infiltrated; Masson’s Trichrome confirmed no fibrosis, and cardiac Troponin and cleaved Caspase-3 immunohistochemistry showed no abnormal signal.

As this is the first mGlu_3_-selective PAM tested in such a toxicology study, it remains to be determined if these findings result from on-target mGlu_3_ activation or if these findings are mediated by an, as of yet unknown, off-target effect. Combined, these data halted the progression of **13l** as an mGlu_3_ PAM clinical candidate, based both on the toxicological findings and on the thiophene core posing substantial developmental risk. Efforts then shifted, and the team focused on other mGlu_3_ PAM HTS chemotypes for progression into development, which will be reported in due course.

## Conclusions

In summary, we disclose the FIC, selective, CNS-penetrant mGlu_3_ PAM (**13l**, VU6053371/BI03738809). A potent and selective mGlu_3_ PAM VU6048261/DI013166572 based on a tetra-substituted thiophene core, but with poor DMPK properties, was identified in a functional high-throughput screening campaign. Chemical lead optimization efforts managed to dramatically improve physicochemical properties, solubility, protein binding, and *in vivo* rat PK to afford VU6053371/BI03738809 with excellent selectivity versus mGlu_2_, as well as the other mGlu receptors. With a FIC *in vivo* tool compound, VU6053371/BI03738809 demonstrated robust efficacy in rat novel object recognition (MED = 3 mg/kg PO) and a clear PK/PD relationship (PD efficacy observed when free brain concentrations were at, or above, the mGlu_3_ EC_50_). Thus, selective activation of mGlu_3_ represents a novel mechanism for addressing the cognitive deficits in schizophrenia and other neurodegenerative diseases. Moreover, the discovery of VU6053371/BI03738809 completes the group II mGlu receptor toolkit of *in vivo* PAM and NAM probes for both mGlu_2_ and mGlu_3_. On the basis of the metabolic liability of the thiophene core as well as the unfavorable toxicological profile in rats, **13l** was not advanced into development; however, it stands as the only rodent *in vivo* tool compound to study selective mGlu_3_ activation.

## Supplementary Material



## Notes

Additional experimental details, methods for the synthesis and characterization of all compounds, *in vitro* and *in vivo* DMPK protocols, and supporting figures (PDF)

## Abbreviations

PAM: positive allosteric modulator
PBL: plasma/brain level
DMPK: drug metabolism and pharmacokinetics
mGlu_3_: metabotropic glutamate receptor subtype 3
MED: minimum effective dose
NOR: novel object recognition
