# Supplementary material for: Discovery of VU6053371/BI03738809: A First-in-Class Selective and CNS-Penetrant mGlu3 Positive Allosteric Modulator (PAM) with Efficacy in a Preclinical Cognition Model
Source: ACS Chem Neurosci. 2026 Jun 6;17(12):2406–18. doi: 10.1021/acschemneuro.6c00325 (PMC13281392; doi:10.1021/acschemneuro.6c00325)
Supplement: Supplementary file 1 [file cn6c00325_si_001.pdf]

Supporting Information

Discovery of VU6053371/BI03738809: a First-in-Class Selective and CNS Penetrant mGlu$_3$ Positive Allosteric Modulator (PAM) with Efficacy in a Preclinical Cognition Model

Caleb A. Jones,[1,2] Renn A. Duncan,[1,2] Kristen M. Gilliland,[1,2] Carson W. Reed,[1,2] Daniel H. Haymer,[1,2] Paul K. Spearing,[1,2] Rory A. Capstick,[1,2] Bartholomew P. Roland,[1,2] Paige Vinson,[1,2] Marq Quitalig,[1,2] Caroline Baggeroer,[1,2] Natasha B Billard,[1,2] Jonathan W. Dickerson, [1,2] Zixiu Xiang,[1,2] Valerie M. Kramlinger,[1,2] Olivier Boutaud,[1,2] Julia Schlichtiger,[6] Carrie K. Jones,[1,2] Colleen M. Niswender,[1,2,4,5] Hyekyung P. Cho,[1,2] Jerri L. Rook,[1,2] Carsten T. Wotjak,[6] Matthias Freiwald,[6] Riccardo Giovanni,[6] Heiko Sommer,[6] Scott Hobson,[6] P. Jeffrey Conn,[1,2] Bruce J. Melancon[1,2] and Craig W. Lindsley*[1,2,3]

Affiliation:
[1]Warren Center for Neuroscience Drug Discovery, Vanderbilt University, Nashville, TN 37232, USA
[2]Department of Pharmacology, Vanderbilt University School of Medicine, Nashville, TN 37232, USA
[3]Department of Chemistry, Vanderbilt University, Nashville TN 37232, USA
[4]Vanderbilt Kennedy Center, Vanderbilt University Medical Center, Nashville, TN 37232, USA
[5]Vanderbilt Brain Institute, Vanderbilt University, Nashville, TN 37232, USA
[6]Boehringer Ingelheim Pharma GmbH & Co. KG, Birkendorfer Str. 65, 88397 Biberach, Germany

*To whom correspondence should be addressed at craig.lindsley@vanderbilt.edu

Table of Contents

General information……………………………………………………………………...S2

Synthetic procedures……………………………………………………………………..S3

In vitro molecular pharmacology methods (cAMP)…..……………………………………..S39

In vitro molecular pharmacology methods (GIRK)…..……………………………………..S43

In vitro and *in vivo* DMPK methods……………………………………………………S45

Behavioral pharmacology methods………………………………………………………S49

Toxicity study methods……………………………………………………………………S50

General Information

Syntheses and manipulations were conducted in air unless otherwise specified. Reaction solvents were purchased from Sigma-Aldrich with a sure seal. All reagents and building blocks for which procedures are not given below were procured from commercial vendors and used without further purification. ^{1}H, $^{13}C\{^{1}H\}$ and 2D NMR spectra were recorded on a 400 MHz Bruker AV-400 spectrometer at ambient temperature unless otherwise noted. ^{1}H and $^{13}C\{^{1}H\}$ chemical shifts are referenced to residual solvent signals (DMSO-d_6 (^{1}H NMR: 2.50 ppm, $^{13}C\{^{1}H\}$ NMR: 39.52 ppm)). Chemical shifts are reported in ppm and multiplicities are abbreviated as follows: br = broad, s = singlet, d = doublet, t = triplet, q = quartet, quint = quintet, dd = doublet of doublets, dt = doublet of triplets, td = triplet of doublets, ddd = doublet of doublet of doublets, tdd = triplet of doublet of doublets, m = multiplet. Automated flash column chromatography (normal phase) was conducted using a Teledyne ISCO CombiFlash system with certified ACS grade solvents. Reverse phase HPLC was performed on a Gilson preparative reverse-phase HPLC system comprised of a 333 aqueous pump with solvent-selection valve, 334 organic pump, GX-271 or GX-281 liquid hander, two column switching valves, and a 155 UV detector. UV wavelength for fraction collection was user-defined, with absorbance at 254 nm always monitored. Column: Phenomenex Axia-packed Gemini C18, 30 x 50 mm, 5 µm. Mobile phase: CH_3CN in H_2O (0.05% v/v NH_4OH). Gradient conditions: 0.75 min equilibration, followed by user-defined gradient (starting organic percentage, ending organic percentage, duration), hold at 95% CH_3CN in H_2O (0.05% v/v NH_4OH) for 1 min, 50 mL/min, 23° C.

High resolution mass spectra were obtained on an Agilent 6540 UHD Q-TOF with Dual AJS source. MS parameters were as follows: fragmentor: 150, capillary voltage: 4000 V, nebulizer pressure: 60 psi, drying gas flow: 13 L/min, drying gas temperature: 275 °C. Samples were introduced via an Agilent 1290 UHPLC comprised of a G4220A binary pump, G4226A ALS, G1316C TCC, and G4212A DAD with ULD flow cell. UV absorption was observed at 215 nm and 254 nm with a 4 nm bandwidth. Column: Waters Acquity BEH C18, 1.0 x 50 mm, 1.7 um. Gradient conditions: 5% to 95% CH_3CN in H_2O (0.1% Formic Acid) over 1.25 min, hold at 95% CH_3CN for 0.25 min, 0.3 mL/min, 40° C.

Synthetic Procedures

General Procedure A:

To a solution of propiophenone (1 equiv.), malononitrile (1.1 equiv.) DMF (1 M) was added L-proline (10 mol %) followed by stirring at room temperature for ten minutes. Sulfur (1.5 equiv.) was then added and the resulting reaction was heated to 60 °C for 16 hr. Upon cooling to room temperature, the reaction mixture was diluted with water and extracted with ethyl acetate. The organic layer was washed with brine, concentrated, and purified via flash chromatography

General Procedure B:

Concentrated sulfuric acid was added to a thiophene-3-carbonitrile, and the resulting reaction was heated to 75 °C for 3 hr (LCMS indicated reaction completion). Upon cooling to room temperature, this reaction mixture was slowly added dropwise to an aqueous solution of 2M NaOH cooled to 0 °C. Once the reaction was quenched and the resulting aqueous layer was made basic (pH ~ 14), ethyl acetate was added, and the resulting layers were separated. The organic layer was concentrated to afford the desired product.

General Procedure C:

A mixture of propiophenone (1 equiv.) 2-cyanoacetamide (2 equiv.) in 1,4-dioxane (1.0 M) was slowly added to a solution of titanium (IV) tetrachloride (3 equiv.) in 1,4-dioxane (0.5 M) at 0 °C. After removing the ice bath, N,N-diisopropylethylamine (3 equiv.) was added and the solution was stirred at room temperature overnight. Solvent was then removed under reduced pressure and the reaction mixture was dissolved in ethyl acetate, washed with water and then brine. Organic layer was collected, dried over anhydrous sodium sulfate, and concentrated. The resulting residue was dissolved in 1,4-dioxane (0.2 M) followed by the addition of sulfur (3 equiv.) and N,N-diisopropylethylamine (3 equiv.). The resulting mixture was stirred at 60 °C for 2-4 hours (determined by LCMS monitoring). Solvent was then removed under reduced pressure and the resulting residue was dissolved in ethyl acetate, washed with water and then brine. Layers separated organic portion dried over sodium sulfate, concentrated under reduced pressure, and purified by flash column chromatography.

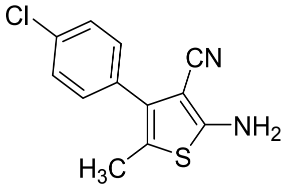

2-amino-4-(4-chlorophenyl)-5-methylthiophene-3-carbonitrile (1):

Compound was prepared via **Procedure A** using 500 mg of 4'-chloropropiophenone and purified via flash chromatography (Teledyne ISCO flash purification system; silica gel column; hexanes:EtOAc; 0-70% EtOAc gradient; solid loading) to afford **(1)** (301 mg, 41 % yield) as a brown solid.

^{1}H NMR (400 MHz, DMSO-d_6) δ 7.55 – 7.46 (m, 2H), 7.38 – 7.29 (m, 2H), 7.11 (s, 2H), 2.12 (s, 3H)

^{13}C{^{1}H} NMR (101 MHz, DMSO-d_6) δ 162.8, 132.7, 132.7, 132.3, 130.8, 128.5, 116.5, 84.1, 12.9

HRMS (ESI) calculated for formula $C_{12}H_{10}ClN_2S$ ($[M+H]^+$) 249.0248, found 249.0245.

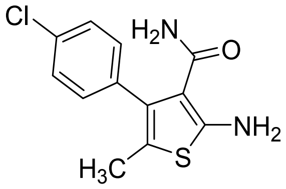

2-amino-4-(4-chlorophenyl)-5-methylthiophene-3-carboxamide (2):

Compound was prepared via **Procedure B** using 301 mg of **(1)** and purified via flash chromatography (Teledyne ISCO flash purification system; silica gel column; hexanes:EtOAc; 0-70% EtOAc gradient; liquid loading) to afford **(2)** (210.1 mg, 65% yield) as an off-white solid.

^{1}H NMR (400 MHz, DMSO-d_6) δ 7.57 – 7.42 (m, 2H), 7.34 – 7.21 (m, 2H), 7.12 (s, 2H), 1.94 (s, 3H)

^{13}C{^{1}H} NMR (101 MHz, DMSO-d_6) δ 167.01, 159.16, 135.21, 132.89, 132.35, 131.69, 128.63, 115.29, 107.17, 12.63

HRMS (ESI) calculated for formula $C_{12}H_{12}ClN_2OS$ ($[M+H]^+$) 267.0353, found 267.0358.

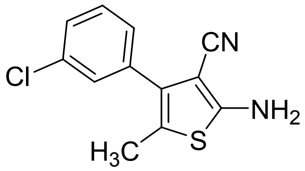

2-amino-4-(3-chlorophenyl)-5-methylthiophene-3-carbonitrile (3):

Compound was prepared via **Procedure A** using 500 mg of 4'-chloropropiophenone and purified via flash chromatography (Teledyne ISCO flash purification system; silica gel column; hexanes:EtOAc; 0-70% EtOAc gradient; solid loading) to afford **(3)** (332.8 mg, 45 % yield) as an orange solid.

^{1}H NMR (400 MHz, DMSO-d_6) δ 7.51 – 7.41 (m, 2H), 7.37 (t, J = 1.7 Hz, 1H), 7.30 (dt, J = 7.4, 1.5 Hz, 1H), 7.13 (s, 2H), 2.13 (s, 3H)

^{13}C{^{1}H} NMR (101 MHz, DMSO-d_6) δ 162.89, 135.95, 133.08, 132.35, 130.33, 128.59, 127.79, 127.57, 116.94, 116.42, 84.02, 12.94

HRMS (ESI) calculated for formula $C_{12}H_{10}ClN_2S$ ([M+H]$^+$) 249.0248, found 249.0251.

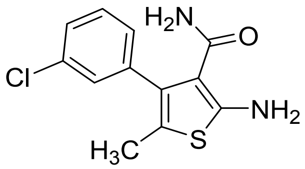

2-amino-4-(3-chlorophenyl)-5-methylthiophene-3-carboxamide (4):

Compound was prepared via **Procedure B** using 333 mg of **(3)** without further purification to afford **(4)** (328 mg, 92 % yield) as an orange solid.

^{1}H NMR (400 MHz, DMSO-d_6) δ 7.52 – 7.41 (m, 2H), 7.32 – 7.25 (m, 1H), 7.21 (dt, J = 7.0, 1.7 Hz, 1H), 1.97 (s, 3H)

^{13}C{^{1}H} NMR (101 MHz, DMSO-d_6) δ 166.91, 158.24, 138.46, 133.14, 132.77, 130.41, 129.51, 128.60, 127.62, 116.09, 107.93, 12.68

HRMS (ESI) calculated for formula $C_{12}H_{12}ClN_2OS$ ([M+H]$^+$) 267.0353, found 267.0357.

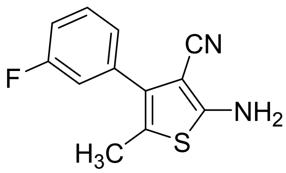

2-amino-4-(3-fluorophenyl)-5-methylthiophene-3-carbonitrile (5):

Compound was prepared via **Procedure A** using 200 mg of 3'-fluoropropiophenone and was purified via flash chromatography (Teledyne ISCO flash purification system; silica gel column; hexanes:EtOAc; 0-70% EtOAc gradient; solid loading) to afford **(5)** (135.9 mg, 44% yield) as a yellow solid.

^{1}H NMR (400 MHz, DMSO-d_6) δ 7.53 – 7.46 (m, 1H), 7.19 (m, 3H), 7.11 (s, 2H), 2.14 (s, 3H)

^{13}C{^{1}H} NMR (101 MHz, DMSO-d_6) δ 162.85, 161.96 (d, J_{CF} = 244.0 Hz), 136.15 (d, J_{CF} = 8.2 Hz), 132.56 (d, J_{CF} = 2.1 Hz), 130.41 (d, J_{CF} = 8.5 Hz), 125.23 (d, J_{CF} = 2.8 Hz), 116.78, 116.44, 115.71 (d, J_{CF} = 21.7 Hz), 114.45 (d, J_{CF} = 20.9 Hz), 84.10, 12.95

HRMS (ESI) calculated for formula $C_{12}H_{10}FN_2S$ ([M+H]$^+$) 233.0543, found 233.0548.

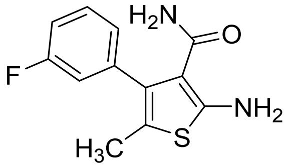

2-amino-4-(3-fluorophenyl)-5-methylthiophene-3-carboxamide (6):

Compound was prepared via **Procedure B** using 7.330 g of **(5)** to give **(6)** 7.978 g (> 99% yield) as a light brown solid without further purification.

^{1}H NMR (400 MHz, DMSO-d_6) δ 7.49 (td, J = 8.1, 6.3 Hz, 1H), 7.23 (tdd, J = 9.3, 2.6, 1.1 Hz, 1H), 7.14 (s, 2H), 7.12 – 7.05 (m, 2H), 1.95 (s, 3H)

^{13}C{^{1}H} NMR (101 MHz, DMSO-d_6) δ 167.45, 162.48 (d, J_{CF} = 244.8 Hz), 159.66, 139.26 (d, J_{CF} = 7.9 Hz), 133.33 (d, J_{CF} = 2.1 Hz), 131.06 (d, J_{CF} = 8.8 Hz), 126.53 (d, J_{CF} = 2.8 Hz), 117.17 (d, J_{CF} = 21.1 Hz), 115.84, 115.02 (d, J_{CF} = 20.7 Hz), 107.60, 13.09

HRMS (ESI) calculated for formula $C_{12}H_{12}FN_2OS$ ([M+H]$^+$) 251.0649, found 251.0652.

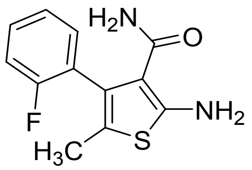

2-amino-4-(2-fluorophenyl)-5-methylthiophene-3-carboxamide (7):

Compound was prepared via **Procedure C** using 150 mg of 2'-fluoropropiophenone and was purified via flash chromatography (Teledyne ISCO flash purification system; silica gel column;

hexanes:EtOAc; 5-100% EtOAc gradient; solid loading) to afford **(7) (**68 mg, 28% yield) as an orange solid.

^{1}H NMR (400 MHz, DMSO-d_6) δ 7.52 – 7.42 (m, 1H), 7.35 – 7.26 (m, 3H), 7.15 (s, 2H), 1.93 (s, 3H).

^{13}C{^{1}H} NMR (101 MHz, DMSO-d_6) δ 167.45, 159.92 (d, J_{CF} = 245.2 Hz), 159.78, 132.65 (d, J_{CF} = 2.9 Hz), 130.89 (d, J_{CF} = 7.9 Hz), 127.61, 125.26 (d, J_{CF} = 3.5 Hz), 124.16 (d, J_{CF} = 16.8 Hz), 116.77, 116.48 (d, J_{CF} = 22.0 Hz), 107.47, 13.10.

HRMS (ESI) calculated for formula $C_{12}H_{12}FN_2OS$ ([M+H]$^+$) 251.0649, found 251.0650.

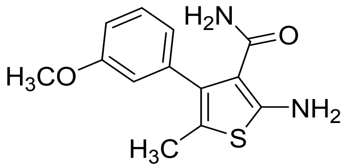

2-amino-4-(3-methoxyphenyl)-5-methylthiophene-3-carboxamide (8):

Compound was prepared via **Procedure C** using 300 mg of 3'-methoxypropiophenone and was purified via flash chromatography (Teledyne ISCO flash purification system; silica gel column; hexanes:EtOAc; 5-100% EtOAc gradient; solid loading) to afford **(8)** (127 mg, 26% yield) as an orange solid.

^{1}H NMR (400 MHz, DMSO-d_6) δ 7.39 (dd, J = 8.3, 7.4 Hz, 1H), 7.26 (s, 1H), 6.98 (ddd, J = 8.4, 2.6, 1.0 Hz, 1H), 6.83 – 6.77 (m, 2H), 3.77 (s, 3H), 1.93 (s, 3H).

^{13}C{^{1}H} NMR (101 MHz, DMSO-d_6) δ 167.59, 160.27, 159.80, 138.45, 134.37, 130.42, 122.41, 115.74, 115.03, 113.86, 107.05, 55.60, 13.11.

HRMS (ESI) calculated for formula $C_{13}H_{15}N_2O_2S$ ([M+H]$^+$) 263.0849, found 263.0849.

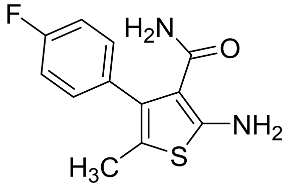

2-amino-4-(4-fluorophenyl)-5-methylthiophene-3-carboxamide (9):

Compound was prepared via **Procedure C** using 250 mg of 4-fluoropropiophenone and was purified via flash chromatography (Teledyne ISCO flash purification system; silica gel column;

hexanes:EtOAc; 5-100% EtOAc gradient; solid loading) to afford **(9)** (66 mg, 16% yield) as a yellow powder.

^{1}H NMR (400 MHz, DMSO-d_6) δ 7.29 (d, J = 7.3 Hz, 4H), 7.18 (s, 2H), 6.72 (bs, 1H), 4.76 (bs, 1H), 1.93 (s, 3H).

^{13}C{^{1}H} NMR (101 MHz, DMSO-d_6) δ 167.10, 161.57 (d, J_{CF} = 244.8 Hz), 159.38, 133.03, 132.67 (d, J_{CF} = 3.2 Hz), 131.90 (d, J_{CF} = 8.2 Hz), 115.61 (d, J_{CF} = 21.4 Hz), 115.07, 106.99, 12.61.

HRMS (ESI) calculated for formula $C_{12}H_{12}FN_2OS$ ($[M+H]^+$) 251.0649, found 251.0646.

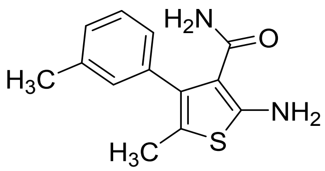

2-amino-5-methyl-4-(m-tolyl)thiophene-3-carboxamide (10):

Compound was prepared via **Procedure C** using 200 mg of 3'-methylpropiophenone and was purified via flash chromatography (Teledyne ISCO flash purification system; silica gel column; hexanes:EtOAc; 5-100% EtOAc gradient; solid loading) to afford **(10)** (75 mg, 23% yield) as an orange solid.

^{1}H NMR (400 MHz, DMSO-d_6) δ 7.36 (t, J = 7.6 Hz, 1H), 7.30 – 7.18 (m, 3H), 7.09 – 6.99 (m, 2H), 2.34 (s, 3H), 1.91 (s, 3H).

^{13}C{^{1}H} (101 MHz, DMSO-d_6) δ 167.17, 159.86, 138.07, 136.57, 134.15, 130.31, 128.71, 128.51, 126.83, 114.41, 106.55, 20.96, 12.64.

HRMS (ESI) calculated for formula $C_{13}H_{15}N_2OS$ ($[M+H]^+$) 247.0900, found 247.0901.

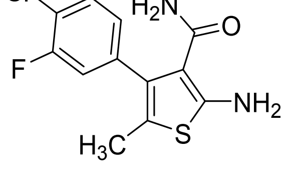

2-amino-4-(4-chloro-3-fluorophenyl)-5-methylthiophene-3-carboxamide (11):

Compound was prepared via **Procedure C** using 200 mg of 4'-chloro-3'-fluoropropiophenone and was purified via flash chromatography (Teledyne ISCO flash purification system; silica gel

column; hexanes:EtOAc; 5-100% EtOAc gradient; solid loading) to afford **(11)** (58 mg, 19% yield) as a dark orange solid.

^{1}H NMR (400 MHz, DMSO-d_6) δ 7.63 (t, J = 8.1 Hz, 1H), 7.29 (dd, J = 10.2, 2.0 Hz, 1H), 7.08 (ddd, J = 8.3, 2.0, 0.7 Hz, 1H), 7.01 (s, 2H), 1.99 (s, 3H).

^{13}C{^{1}H} NMR (101 MHz, DMSO-d_6) δ 166.87, 158.56, 156.91 (d, J_{CF} = 247.3 Hz), 137.35 (d, J_{CF} = 7.4 Hz), 132.06, 130.51, 127.28 (d, J_{CF} = 3.3 Hz), 118.61, 118.32 (d, J_{CF} = 21.0 Hz), 116.05, 107.64, 12.66.

HRMS (ESI) calculated for formula $C_{12}H_{11}ClFN_2OS$ ($[M+H]^+$) 285.0259, found 285.0261.

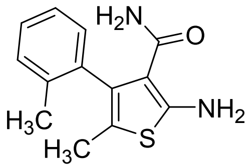

2-amino-5-methyl-4-(o-tolyl)thiophene-3-carboxamide (12):

Compound was prepared via **Procedure C** using 200 mg of 2'-methylpropiophenone and was purified via flash chromatography (Teledyne ISCO flash purification system; silica gel column; hexanes:EtOAc; 5-100% EtOAc gradient; solid loading) to afford **(12)** (71 mg, 21% yield) as an orange solid.

^{1}H NMR (400 MHz, DMSO-d_6) δ 7.44 – 7.26 (m, 5H), 7.18 – 7.12 (m, 1H), 2.05 (s, 3H), 1.82 (s, 3H).

^{13}C{^{1}H} NMR (101 MHz, DMSO-d_6) δ 167.68, 161.13, 137.65, 136.74, 133.53, 130.86, 130.46, 128.92, 127.00, 114.20, 106.01, 19.69, 12.86.

HRMS (ESI) calculated for formula $C_{13}H_{15}N_2OS$ ($[M+H]^+$) 247.0900, found 247.0901.

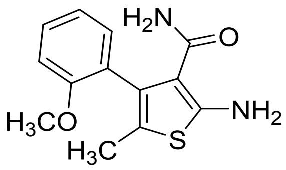

2-amino-4-(2-methoxyphenyl)-5-methylthiophene-3-carboxamide (13):

Compound was prepared via **Procedure C** using 200 mg of 1-(2-methoxyphenyl)propan-1-one and was purified via flash chromatography (Teledyne ISCO flash purification system; silica gel

column; hexanes:EtOAc; 5-100% EtOAc gradient; solid loading) to afford **(13)** (78 mg, 24% yield) as an orange solid.

^{1}H NMR (400 MHz, DMSO-d_6) δ 7.36 (ddd, J = 8.2, 7.4, 1.8 Hz, 1H), 7.11 (ddd, J = 12.1, 7.9, 1.4 Hz, 2H), 6.99 (td, J = 7.4, 1.1 Hz, 1H), 6.88 (d, J = 6.0 Hz, 2H), 3.75 (s, 3H), 1.98 (s, 3H).

^{13}C{^{1}H} NMR (101 MHz, DMSO-d_6) δ 161.70, 156.83, 131.14, 131.04, 129.49, 122.63, 120.23, 116.51, 116.47, 111.59, 85.86, 55.26, 13.01.

HRMS (ESI) calculated for formula $C_{13}H_{15}N_2O_2S$ ([M+H]$^+$) 263.0849, found 263.0846.

Conditions for PyClU amide couplings:

Method D:

In a Biotage microwave vial a carboxylic acid (1.0 equiv.), Chlorodipyrrolidinocarbenium hexafluorophosphate (PyClU) (1.5 equiv), and a thiophene (1.5 equiv.) was dissolved in N-methyl-pyrrolidone (0.15 M) followed by the addition of *N,N*-diisopropylethylamine (4 equiv.). The reaction vessel was then sealed and was heated to 150 °C via microwave irradiation using a Biotage microwave reactor and stirred for 45 minutes. Upon completion the mixture was filtered through a PTFE filter and purified by reverse phase HPLC.

Method E:

In a Biotage microwave vial a carboxylic acid (1.5 equiv.), Chlorodipyrrolidinocarbenium hexafluorophosphate (PyClU) (1.5 equiv), and a thiophene (1.0 equiv.) was dissolved in N-methyl-pyrrolidone (0.15 M) followed by the addition of *N,N*-diisopropylethylamine (3 equiv.). The reaction vessel was then sealed and was heated to 140 °C via microwave irradiation using a Biotage microwave reactor and stirred for 40 minutes. Upon completion the mixture was diluted to 1 mL with N-methyl-pyrrolidone, filtered through a PTFE filter and purified by reverse phase HPLC.

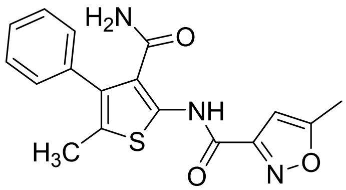

***N*-(3-carbamoyl-5-methyl-4-phenylthiophen-2-yl)-5-methylisoxazole-3-carboxamide (10a):**

Compound was prepared via **Method D** using 12 mg of 5-methylisoxazole-3-carboxylic acid and purified by reverse phase HPLC (Gilson 30x100 mm column, 20-65% acetonitrile/ 0.01 aqueous NH_4OH 10 minute run) then via flash chromatography (Teledyne ISCO flash purification system; silica gel column; hexanes:EtOAc; 0-50% EtOAc gradient; liquid loading) to give **(10a)** (10 mg, 31 % yield) as an opaque solid.

^1H NMR (400 MHz, DMSO-d_6) δ 7.65 (s, 1H), 7.56 – 7.44 (m, 3H), 7.37 – 7.30 (m, 2H), 6.75 (d, J = 1.1 Hz, 1H), 5.32 (s, 1H), 2.52 (s, 3H), 2.12 (s, 3H).

$^{13}C\{^1H\}$ NMR (101 MHz, DMSO-d_6) δ 172.71, 166.59, 157.62, 155.29, 141.72, 134.95, 133.53, 130.00, 129.07, 128.39, 126.40, 116.45, 101.54, 12.65, 11.97.

HRMS (ESI) calculated for formula $C_{17}H_{16}N_3O_3S$ ($[M+H]^+$) 342.0907, found 342.0913.

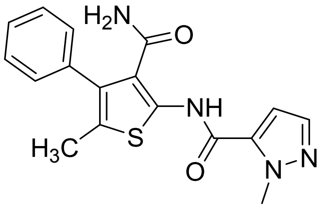

***N*-(3-carbamoyl-5-methyl-4-phenylthiophen-2-yl)-1-methyl-1*H*-pyrazole-5-carboxamide (6):**

Compound was prepared via **Method D** using 15 mg of 1-methylpyrazole-5-carboxylic acid and was purified using reverse phase HPLC (Gilson 30x100 mm column, 25-65% acetonitrile/ 0.01 aqueous NH_4OH 10 minute run) to give **(6) (**12.3 mg, 30 % yield) as a white solid.

^1H NMR (400 MHz, DMSO-d_6) δ 8.46 (s, 1H), 7.56 – 7.42 (m, 4H), 7.33 (dd, 2H), 5.27 (s, 1H), 2.67 (s, 3H), 2.11 (s, 3H).

$^{13}C\{^1H\}$ NMR (101 MHz, DMSO-d_6) δ 166.51, 158.04, 154.55, 150.33, 142.26, 135.22, 133.27, 130.02, 128.99, 128.25, 127.16, 125.68, 115.76, 12.64, 11.38.

HRMS (ESI) calculated for formula $C_{17}H_{17}N_4O_2S$ ($[M+H]^+$) 341.1067, found 341.1067.

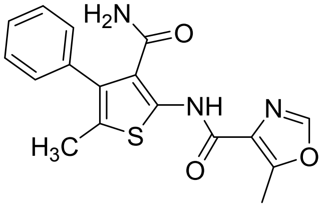

***N*-(3-carbamoyl-5-methyl-4-phenylthiophen-2-yl)-5-methyloxazole-4-carboxamide (10b):**

Compound was prepared via **Method D** using 15 mg of 5-methyloxazole-4-carboxylic acid and purified by reverse phase HPLC (Gilson 30x100 mm column, 20-65% acetonitrile/ 0.01 aqueous NH_4OH 10 minute run) then via flash chromatography (Teledyne ISCO flash purification system; silica gel column; hexanes:EtOAc; 0-50% EtOAc gradient; liquid loading) to give **(10b)** (27 mg, 67 % yield) as a white solid.

^{1}H NMR (400 MHz, DMSO-d_6) δ 8.46 (s, 1H), 7.57 – 7.42 (m, 4H), 7.35 – 7.31 (m, 2H), 5.27 (s, 1H), 2.67 (s, 3H), 2.10 (s, 3H).

^{13}C{^{1}H} NMR (101 MHz, DMSO-d_6) δ 166.51, 158.04, 154.54, 150.33, 142.26, 135.22, 133.27, 130.01, 128.99, 128.25, 127.16, 125.68, 115.75, 12.64, 11.38.

HRMS (ESI) calculated for formula $C_{17}H_{16}N_3O_3S$ ([M+H]$^+$) 342.0907, found 342.0910.

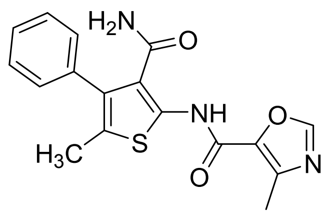

***N*-(3-carbamoyl-5-methyl-4-phenylthiophen-2-yl)-4-methyloxazole-5-carboxamide (10c):**

In a microwave vial was added 4-methyl-1,3-oxazole-5-carboxylic acid (328.3 mg, 2.58 mmol), PyClU (859.2 mg, 2.58 mmol), 2-amino-5-methyl-4-phenyl-thiophene-3-carboxamide (500 mg, 2.15 mmol), and 2-amino-5-methyl-4-phenyl-thiophene-3-carboxamide (276.3 mg, 1.19 mmol) in NMP (10 mL). Reaction was heated to 150 °C via microwave irradiation and stirred for 45 minutes. Mixture was filtered through a PTFE filter purified by reverse phase HPLC (Gilson 30x100 mm column, 35-80% acetonitrile/ 0.01 aqueous NH_4OH 10 minute run) to give **(10c)** (283 mg, 39% yield) as a white solid.

^{1}H NMR (400 MHz, DMSO-d_6) δ 8.58 (d, J = 0.6 Hz, 1H), 7.62 (s, 1H), 7.57 – 7.43 (m, 3H), 7.37 – 7.30 (m, 2H), 5.28 (s, 1H), 2.48 (s, 3H), 2.10 (s, 3H).

^{13}C{^{1}H} NMR (101 MHz, DMSO-d_6) δ 166.81, 153.96, 152.42, 143.00, 142.43, 137.89, 135.04, 133.32, 130.02, 129.09, 128.40, 126.11, 115.58, 12.70, 12.61.

HRMS (ESI) calculated for formula $C_{17}H_{16}N_3O_3S$ ([M+H]$^+$) 342.0907, found 342.0906.

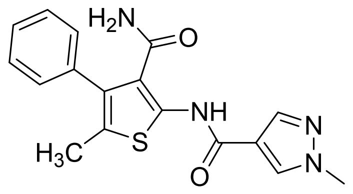

***N*-(3-carbamoyl-5-methyl-4-phenylthiophen-2-yl)-1-methyl-1*H*-pyrazole-4-carboxamide (10d):**

Compound was prepared via **Method E** using 25 mg of 2-amino-5-methyl-4-phenylthiophene-3-carboxamide and was purified using reverse phase HPLC (Gilson 30x100 mm column, 20-70% acetonitrile/ 0.01 aqueous NH_4OH 10 minute run) to give **(10d**) (8 mg, 22 % yield) as an off-white solid.

^{1}H NMR (400 MHz, DMSO-d_6) δ 8.34 (s, 1H), 7.84 (s, 1H), 7.56 – 7.46 (m, 3H), 7.35 – 7.29 (m, 2H), 5.33 (s, 1H), 3.92 (s, 3H), 2.09 (s, 3H).

^{13}C{^{1}H} NMR (101 MHz, DMSO-d_6) δ 167.05, 158.37, 143.52, 138.17, 135.30, 133.18, 132.55, 129.98, 129.04, 128.27, 125.33, 116.56, 114.81, 38.99, 12.60.

HRMS (ESI) calculated for formula $C_{17}H_{16}N_4O_2S$ ([M+H]$^+$) 341.1067, found 341.1067.

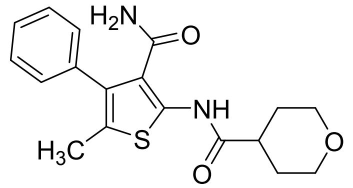

***N*-(3-carbamoyl-5-methyl-4-phenylthiophen-2-yl)tetrahydro-2*H*-pyran-4-carboxamide (10e):**

Compound was prepared via **Method D** using 15 mg of 4-oxanic acid and was purified using reverse phase HPLC (Gilson 30x100 mm column, 20-65% acetonitrile/ 0.01 aqueous NH_4OH 10 minute run) then via flash chromatography (Teledyne ISCO flash purification system; silica gel column; hexanes:EtOAc; 0-50% EtOAc gradient; liquid loading) to give **(10e)** (18 mg, 45 % yield) as a white solid.

^{1}H NMR (400 MHz, DMSO-d_6) δ 8.19 – 7.78 (m, 4H), 7.79 – 7.58 (m, 2H), 5.92 (bs, 1H), 4.40 – 4.24 (m, 2H), 3.80 (td, 2H), 3.36 – 3.10 (m, 1H), 2.50 (s, 3H), 2.38 – 2.16 (m, 2H), 2.13 – 1.96 (m, 2H).

^{13}C{^{1}H} NMR (101 MHz, DMSO-d_6) δ 171.31, 166.75, 142.18, 135.35, 133.12, 129.90, 128.90, 128.09, 125.21, 115.88, 66.21, 40.83, 28.70, 12.60.

HRMS (ESI) calculated for formula $C_{18}H_{21}N_2O_3S$ ([M+H]$^+$) 345.1267, found 345.1274.

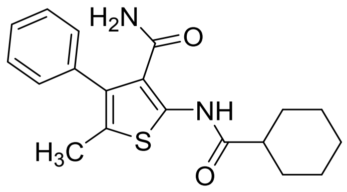

2-(cyclohexanecarboxamido)-5-methyl-4-phenylthiophene-3-carboxamide (10f):

Compound was prepared via **Method D** using 10.8 mg of cyclohexanecarboxylic acid and was purified using reverse phase HPLC (Gilson 30x100 mm column, 25-80% acetonitrile/ 0.01 aqueous NH_4OH 10 minute run) then via flash chromatography (Teledyne ISCO flash purification system; silica gel column; hexanes:EtOAc; 0-30% EtOAc gradient; liquid loading) to give **(10f)** (13 mg, 45 % yield) as a white solid.

^{1}H NMR (400 MHz, DMSO-d_6) δ 7.65 – 7.38 (m, 4H), 7.36 – 7.07 (m, 2H), 5.43 (bs, 1H), 2.44 (tt, J = 11.1, 3.5 Hz, 1H), 2.07 (s, 3H), 1.99 – 1.82 (m, 3H), 1.73 (dt, J = 12.7, 3.7 Hz, 2H), 1.68 – 1.56 (m, 1H), 1.52 – 1.11 (m, 6H).

^{13}C{^{1}H} NMR (101 MHz, DMSO-d_6) δ 172.41, 166.80, 142.60, 135.40, 133.05, 129.91, 128.91, 128.09, 125.04, 115.43, 43.89, 28.94, 25.32, 24.99, 12.57.

HRMS (ESI) calculated for formula $C_{19}H_{23}N_2O_2S$ ($[M+H]^+$) 343.1475, found 343.1476.

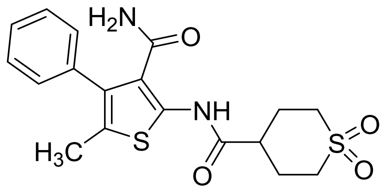

***N*-(3-carbamoyl-5-methyl-4-phenylthiophen-2-yl)tetrahydro-2*H*-thiopyran-4-carboxamide 1,1-dioxide (10g):**

Compound was prepared via **Method D** using 15 mg of tetrahydro-2h-thiopyransulfone-4-carboxylic acid and was purified using reverse phase HPLC (Gilson 30x100 mm column, 15-60% acetonitrile/ 0.01 aqueous NH_4OH 10 minute run) then via flash chromatography (Teledyne ISCO flash purification system; silica gel column; hexanes:EtOAc; 0-50% EtOAc gradient; liquid loading) to give **(10g)** (13 mg, 39% yield) as a white solid.

^{1}H NMR (400 MHz, DMSO-d_6) δ 7.58 – 7.38 (m, 4H), 7.36 – 7.23 (m, 2H), 5.67 (s, 1H), 3.30 – 3.20 (m, 2H), 3.20 – 3.04 (m, 2H), 2.99 – 2.81 (m, 1H), 2.38 – 2.19 (m, 2H), 2.16 – 2.00 (m, 5H).

$^{13}C\{^1H\}$ NMR (101 MHz, DMSO-d_6) δ 170.44, 166.61, 141.17, 135.29, 133.22, 129.85, 128.84, 128.02, 125.56, 116.87, 49.37, 40.43, 27.10, 12.62.
HRMS (ESI) calculated for formula $C_{18}H_{21}N_2O_4S_2$ ($[M+H]^+$) 393.0937, found 393.0940.

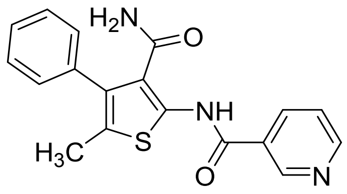

***N*-(3-carbamoyl-5-methyl-4-phenylthiophen-2-yl)nicotinamide (10h):**
Compound was prepared via **Method D** using 10.4 mg of nicotinic acid and was purified using reverse phase HPLC (Gilson 30x100 mm column, 25-65% acetonitrile/ 0.01 aqueous NH_4OH 10 minute run) then via flash chromatography (Teledyne ISCO flash purification system; silica gel column; hexanes:EtOAc; 0-100% EtOAc gradient; liquid loading) to give **(10h)** (9 mg, 32% yield) as a yellow solid.
^1H NMR (400 MHz, DMSO-d_6) δ 9.08 (d, J = 1.8 Hz, 1H), 8.83 (dd, J = 4.8, 1.6 Hz, 1H), 8.29 – 8.22 (m, 1H), 7.65-7.67 (m, 2H), 7.57 – 7.44 (m, 3H), 7.41 – 7.29 (m, 2H), 5.49 (s, 1H), 2.13 (s, 3H).
$^{13}C\{^1H\}$ NMR (101 MHz, DMSO-d_6) δ 167.02, 161.48, 153.03, 148.15, 142.75, 135.11, 134.99, 133.49, 129.96, 129.05, 128.32, 128.16, 126.20, 124.13, 116.32, 12.67.
HRMS (ESI) calculated for formula $C_{18}H_{16}N_3O_2S$ ($[M+H]^+$) 338.0958, found 338.0960.

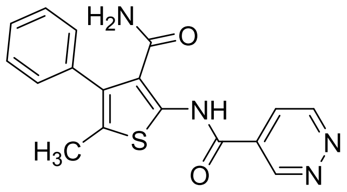

***N*-(3-carbamoyl-5-methyl-4-phenylthiophen-2-yl)pyridazine-4-carboxamide (10i):**
Compound was prepared via **Method D** using 10.4 mg of pyridazine-4-carboxylic acid and was purified using reverse phase HPLC (Gilson 30x100 mm column, 15-60% acetonitrile/ 0.01 aqueous NH_4OH 10 minute run) then via flash chromatography (Teledyne ISCO flash purification system; silica gel column; MeOH:DCM; 0-10% MeOH gradient) to give **(10i)** (6.8 mg, 16 % yield) as a yellow solid

^{1}H NMR (400 MHz, DMSO-d_6) δ 9.58 (dd, J = 2.4, 1.3 Hz, 1H), 9.55 (dd, J = 5.3, 1.3 Hz, 1H), 8.04 (dd, J = 5.3, 2.4 Hz, 1H), 7.70 (s, 1H), 7.56 – 7.42 (m, 3H), 7.37 – 7.29 (m, 2H), 5.76 (s, 1H), 2.16 (s, 3H).

$^{13}C\{^1H\}$ NMR (101 MHz, DMSO-d_6) δ 166.67, 160.22, 152.46, 148.31, 141.04, 134.96, 133.77, 130.14, 129.88, 128.95, 128.22, 126.96, 124.13, 118.09, 12.76.

HRMS (ESI) calculated for formula $C_{17}H_{15}N_4O_2S$ ($[M+H]^+$) 339.0910, found 339.0910.

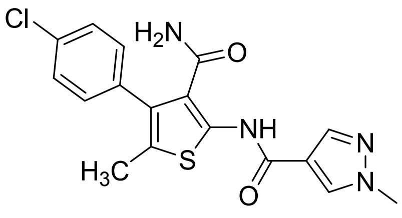

***N*-(3-carbamoyl-4-(4-chlorophenyl)-5-methylthiophen-2-yl)-1-methyl-1*H*-pyrazole-4-carboxamide (11a):**

Compound was prepared via **Method E** using 25 mg of **(2)** and was purified using reverse phase HPLC (Gilson 30x100 mm column, 20-70% acetonitrile/ 0.01 aqueous NH_4OH 10 minute run) to give **(11a)** (8.4 mg, 24 % yield) as an off-white solid.

^{1}H NMR (400 MHz, DMSO-d_6) δ 8.34 (s, 1H), 7.85 (s, 1H), 7.58 – 7.52 (m, 2H), 7.36 – 7.30 (m, 2H), 3.92 (s, 3H), 2.12 (s, 3H).

$^{13}C\{^1H\}$ NMR (101 MHz, DMSO-d_6) δ 166.86, 158.48, 142.64, 138.25, 133.99, 132.80, 132.60, 131.99, 131.89, 128.82, 125.96, 116.51, 115.80, 38.99, 12.65.

HRMS (ESI) calculated for formula $C_{17}H_{16}ClN_4O_2S$ ($[M+H]^+$) 375.0677, found 375.0676.

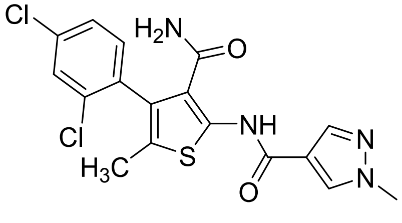

***N*-(3-carbamoyl-4-(2,4-dichlorophenyl)-5-methylthiophen-2-yl)-1-methyl-1*H*-pyrazole-4-carboxamide (11b):**

Compound was prepared via **Method E** using 28 mg of 2-amino-4-(2,4-dichlorophenyl)-5-methylthiophene-3-carboxamide and was purified using reverse phase HPLC (Gilson 30x100 mm

column, 20-70% acetonitrile/ 0.01 aqueous NH_4OH 10 minute run) to give **(11b)** (7.7 mg , 20 % yield) as a beige solid.

^{1}H NMR (400 MHz, DMSO-*d₆*) δ 8.34 (s, 1H), 7.85 (s, 1H), 7.80 (d, J = 2.2 Hz, 1H), 7.57 (dd, J = 8.2, 2.2 Hz, 1H), 7.46 (d, J = 8.2 Hz, 1H), 3.92 (s, 3H), 2.04 (s, 3H).

^{13}C{^{1}H} NMR (101 MHz, DMSO-d_6) δ 167.17, 158.94, 143.57, 138.68, 135.16, 134.55, 134.08, 133.37, 133.11, 129.94, 129.55, 128.51, 127.34, 116.94, 115.73, 39.46, 12.95.

HRMS (ESI) calculated for formula $C_{17}H_{15}Cl_2N_4O_2S$ ($[M+H]^+$) 409.0287, found 409.0284.

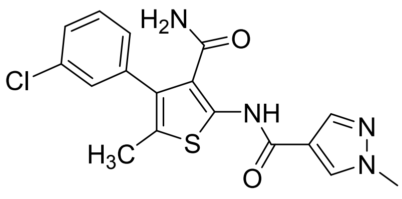

***N*-(3-carbamoyl-4-(3-chlorophenyl)-5-methylthiophen-2-yl)-1-methyl-1*H*-pyrazole-4-carboxamide (11c):**

Compound was prepared via **Method E** using 25 mg of **(4)** and was purified using reverse phase HPLC (Gilson 30x100 mm column, 20-70% acetonitrile/ 0.01 aqueous NH_4OH 10 minute run) to give **(11c)** (8 mg, 23 % yield) as an off-white powder.

^{1}H NMR (400 MHz, DMSO-d_6) δ 8.35 (s, 1H), 7.86 (s, 1H), 7.56 – 7.46 (m, 2H), 7.39 (s, 1H), 7.33 – 7.25 (m, 1H), 3.92 (s, 3H), 2.13 (s, 3H).

^{13}C{^{1}H} NMR (101 MHz, DMSO-d_6) δ 166.77, 158.52, 142.37, 138.28, 137.30, 133.28, 132.60, 131.79, 130.54, 129.70, 128.79, 127.98, 126.25, 116.49, 116.08, 38.98, 12.66.

HRMS (ESI) calculated for formula $C_{17}H_{16}ClN_4O_2S$ ($[M+H]^+$) 375.0677, found 375.0675.

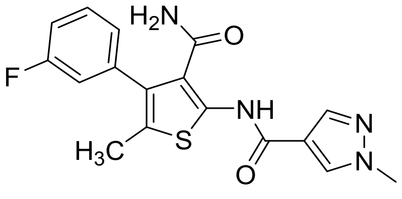

***N*-(3-carbamoyl-4-(3-fluorophenyl)-5-methylthiophen-2-yl)-1-methyl-1*H*-pyrazole-4-carboxamide (11d):**

Compound was prepared via **Method E** using 25 mg of **(6)** and was purified using reverse phase HPLC (Gilson 30x100 mm column, 20-70% acetonitrile/ 0.01 aqueous NH_4OH 10 minute run) to give **(11d)** (7.2 mg, 20 % yield) as a beige solid.

^{1}H NMR (400 MHz, DMSO-d_6) δ 8.34 (s, 1H), 7.86 (s, 1H), 7.54 (td, J = 8.1, 6.3 Hz, 1H), 7.28 (td, J = 8.7, 2.7 Hz, 1H), 7.23 – 7.10 (m, 2H), 5.75 (s, 1H), 3.92 (s, 3H), 2.13 (s, 3H).
$^{13}C\{^{1}H\}$ NMR (101 MHz, DMSO-d_6) δ 166.83, 162.09 (d, J_{CF} = 245.1 Hz), 158.49, 142.68, 138.26, 137.53 (d, J_{CF} = 8.0 Hz), 132.60, 131.95 (d, J_{CF} = 2.0 Hz), 130.80 (d, J_{CF} = 8.7 Hz), 126.25 (d, v = 2.8 Hz), 126.04, 116.96 (d, J_{CF} = 21.3 Hz), 116.51, 115.75, 114.98 (d, J_{CF} = 20.8 Hz), 30.71, 12.63.
HRMS (ESI) calculated for formula $C_{17}H_{16}FN_4O_2S$ ($[M+H]^+$) 359.0973, found 359.0968.

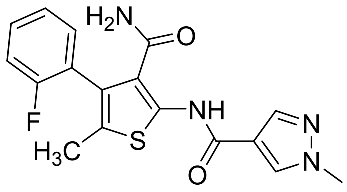

***N*-(3-carbamoyl-4-(2-fluorophenyl)-5-methylthiophen-2-yl)-1-methyl-1*H*-pyrazole-4-carboxamide (11e):**

Compound was prepared via **Method E** using 25 mg of **(7)** and was purified using reverse phase HPLC (Gilson 30x100 mm column, 20-70% acetonitrile/ 0.01 aqueous NH_4OH 10 minute run) to give **(11e)** (7.8 mg, 22 % yield) as a beige solid.
^{1}H NMR (400 MHz, DMSO-d_6) δ 8.34 (s, 1H), 7.86 (s, 1H), 7.58 – 7.48 (m, 1H), 7.43 – 7.30 (m, 3H), 3.92 (s, 3H), 2.11 (s, 3H).
$^{13}C\{^{1}H\}$ NMR (101 MHz, DMSO-d_6) δ 166.85, 159.39 (d, J_{CF} = 245.3 Hz), 158.45, 142.90, 138.24, 132.61, 132.37 (d, J_{CF} = 2.8 Hz), 130.83 (d, J_{CF} = 8.1 Hz), 127.00, 126.33, 124.93 (d, J_{CF} = 3.5 Hz), 122.42 (d, J_{CF} = 16.2 Hz), 116.49, 116.12 (d, J_{CF} = 21.9 Hz), 115.67, 30.71, 12.63.
HRMS (ESI) calculated for formula $C_{17}H_{16}FN_4O_2S$ ($[M+H]^+$) 359.0973, found 359.0971.

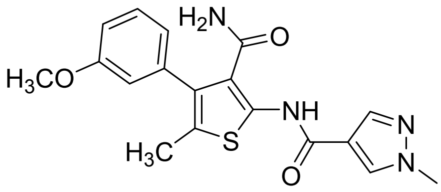

***N*-(3-carbamoyl-4-(3-methoxyphenyl)-5-methylthiophen-2-yl)-1-methyl-1*H*-pyrazole-4-carboxamide (11i):**

Compound was prepared via **Method E** using 25 mg of **(8)** and was purified using reverse phase HPLC (Gilson 30x100 mm column, 20-70% acetonitrile/ 0.01 aqueous NH_4OH 10 minute run) to give **(11i)** (8 mg, 23% yield) as a beige solid.

^{1}H NMR (400 MHz, DMSO-d_6) δ 8.34 (s, 1H), 7.84 (s, 1H), 7.55 (s, 1H), 7.47 – 7.40 (m, 1H), 7.03 (ddd, J = 8.4, 2.5, 1.1 Hz, 1H), 6.92 – 6.83 (m, 2H), 3.92 (s, 3H), 3.79 (s, 3H), 2.10 (s, 3H).

$^{13}C\{^{1}H\}$ NMR (101 MHz, DMSO-d_6) δ 167.49, 159.96, 158.82, 144.07, 138.62, 137.16, 133.46, 133.01, 130.68, 125.76, 122.50, 117.03, 115.92, 115.12, 114.33, 55.67, 39.46, 13.07.

HRMS (ESI) calculated for formula $C_{18}H_{19}N_4O_3S$ ([M+H]$^+$) 371.1172, found 371.1174.

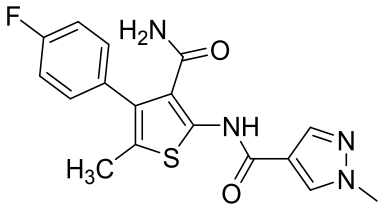

***N*-(3-carbamoyl-4-(4-fluorophenyl)-5-methylthiophen-2-yl)-1-methyl-1*H*-pyrazole-4-carboxamide (11f):**

Compound was prepared via **Method E** using 25 mg of **(9)** and was purified using reverse phase HPLC (Gilson 30x100 mm column, 20-70% acetonitrile/ 0.01 aqueous NH_4OH 10 minute run) to give **(11f)** (11 mg, 31 % yield) as a light tan solid.

^{1}H NMR (400 MHz, DMSO-d_6) δ 8.34 (s, 1H), 7.85 (s, 1H), 7.54 (s, 1H), 7.42 – 7.28 (m, 4H), 3.92 (s, 3H), 2.10 (s, 3H).

$^{13}C\{^{1}H\}$ NMR (101 MHz, DMSO-d_6) δ 166.96, 161.84 (d, J_{CF} = 245.2 Hz), 158.42, 142.95, 138.20, 132.57, 132.15, 132.14 (d, J_{CF} = 8.3 Hz), 131.40 (d, J_{CF} = 3.3 Hz), 125.76, 116.53, 115.82 (d, J_{CF} = 21.4 Hz), 115.42, 12.61.

HRMS (ESI) calculated for formula $C_{17}H_{16}FN_4O_2S$ ([M+H]$^+$) 359.0973, found 359.0972.

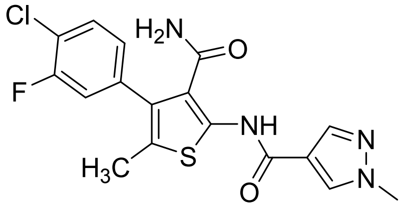

***N*-(3-carbamoyl-4-(4-chloro-3-fluorophenyl)-5-methylthiophen-2-yl)-1-methyl-1*H*-pyrazole-4-carboxamide (11g):**

Compound was prepared via **Method E** using 25 mg of **(11)** and was purified using reverse phase HPLC (Gilson 30x100 mm column, 20-70% acetonitrile/ 0.01 aqueous NH_4OH 10 minute run) to give **(11g)** (7 mg, 20 % yield) as a beige solid.

^{1}H NMR (400 MHz, DMSO-d_6) δ 8.34 (s, 1H), 7.86 (d, J = 0.8 Hz, 1H), 7.67 (t, J = 8.1 Hz, 1H), 7.39 (dd, J = 10.2, 2.0 Hz, 1H), 7.18 – 7.12 (m, 1H), 3.92 (s, 3H), 2.16 (s, 3H).

^{13}C{^{1}H} NMR (101 MHz, DMSO-d_6) δ 166.67, 158.57, 157.02 (d, J_{CF} = 247.3 Hz), 142.04, 138.31, 136.13 (d, J_{CF} = 7.5 Hz), 132.64, 131.10, 130.70, 127.48 (d, J_{CF} = 3.5 Hz), 126.61, 119.02 (d, J_{CF} = 17.3 Hz), 118.60 (d, J_{CF} = 20.9 Hz), 116.48, 116.44, 12.68.

HRMS (ESI) calculated for formula $C_{17}H_{15}ClFN_4O_2S$ ($[M+H]^+$) 393.0583, found 393.0580.

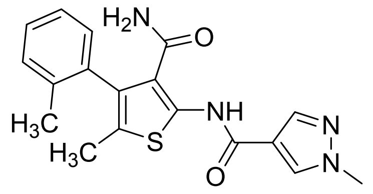

***N*-(3-carbamoyl-5-methyl-4-(o-tolyl)thiophen-2-yl)-1-methyl-1*H*-pyrazole-4-carboxamide (11h):**

Compound was prepared via **Method E** using 25 mg of **(12)** and was purified using reverse phase HPLC (Gilson 30x100 mm column, 20-70% acetonitrile/ 0.01 aqueous NH_4OH 10 minute run) to give **(11h) (**7 mg, 19 % yield) as a beige solid.

^{1}H NMR (400 MHz, DMSO-d_6) δ 8.34 (s, 1H), 7.84 (s, 1H), 7.51 (s, 1H), 7.44 – 7.33 (m, 3H), 7.25 – 7.19 (m, 1H), 3.93 (s, 3H), 2.04 (s, 3H), 1.99 (s, 3H).

^{13}C{^{1}H} NMR (101 MHz, DMSO-d_6) δ 167.62, 158.74, 145.25, 138.56, 137.68, 135.34, 133.04, 132.58, 131.15, 130.59, 129.45, 127.33, 125.14, 117.04, 113.78, 39.47, 19.72, 12.8

HRMS (ESI) calculated for formula $C_{18}H_{19}N_4O_2S$ ($[M+H]^+$) 355.1223, found 355.1223.

Conditions for Buchwald couplings:

A vial was charged with aryl bromide (1 equiv.) , thiophene (1.5 equiv) , potassium carbonate (3 equiv.), tris(dibenzylideneacetone)dipalladium (5 mol %) , Brettphos (10 mol %) , and 1,4-dioxane (0.1 M) . The reaction mixture was sparged with nitrogen for one minute before the reaction vial was sealed, heated to 100 °C for 4-16 hours. Upon cooling to room temperature, the

crude reaction was diluted with ethyl acetate, filtered through a PTFE filter, concentrated and either purified by flash chromatography (and) or reverse phase HPLC.

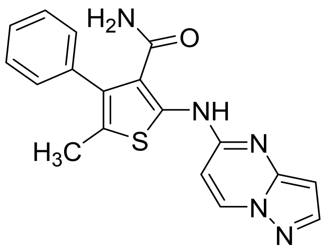

5-methyl-4-phenyl-2-(pyrazolo[1,5-a]pyrimidin-5-ylamino)thiophene-3-carboxamide (12b): Compound was prepared via the general procedure for **Buchwald couplings** using 14 mg of 5-chloropyrazolo[1,5-a]pyrimidine and was purified using reverse phase HPLC (Gilson 30x100 mm column, 20-70% acetonitrile/ 0.01 aqueous NH_4OH 10 minute run) then via flash chromatography (Teledyne ISCO flash purification system; silica gel column; hexanes:EtOAc; 0-30% EtOAc gradient; liquid loading) to give **(12b)** (8 mg, 25 % yield) as an off-white solid

^{1}H NMR (400 MHz, DMSO-d_6) δ 8.77 (dd, J = 7.5, 0.9 Hz, 1H), 7.99 (d, J = 2.1 Hz, 1H), 7.55 – 7.39 (m, 4H), 7.36 – 7.29 (m, 2H), 6.82 (d, J = 7.5 Hz, 1H), 6.32 (dd, J = 2.2, 0.8 Hz, 1H), 5.59 (s, 1H), 2.14 (s, 3H).

^{13}C{^{1}H} NMR (101 MHz, DMSO-d_6) δ 167.05, 149.53, 146.44, 144.51, 143.48, 136.40, 135.61, 133.27, 129.89, 128.82, 127.94, 124.47, 115.11, 100.64, 93.05, 12.60

HRMS (ESI) calculated for formula $C_{18}H_{16}N_5OS$ ($[M+H]^+$) 350.1070, found 350.1074.

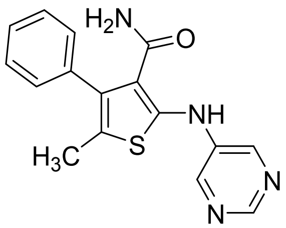

5-methyl-4-phenyl-2-(pyrimidin-5-ylamino)thiophene-3-carboxamide (12d):

Compound was prepared via the general procedure for **Buchwald couplings** using 15 mg of 5-bromopyrimidine and was purified using reverse phase HPLC (Gilson 30x100 mm column, 10-60% acetonitrile/ 0.01 aqueous NH_4OH 10 minute run) then via flash chromatography (Teledyne ISCO flash purification system; silica gel column; hexanes:EtOAc; 0-4% MeOH/DCM gradient; liquid loading) to give **(12d)** (11 mg, 38 % yield) as an opaque oil.

^{1}H NMR (400 MHz, DMSO-d_6) δ 8.92 (s, 1H), 8.63 (s, 1H), 8.45 (s, 2H), 7.43 (tt, J = 6.8, 1.1 Hz, 2H), 7.39 – 7.31 (m, 3H), 7.25 (s, 1H), 6.85 (s, 1H), 2.22 (s, 3H).
$^{13}C\{^{1}H\}$ NMR (101 MHz, DMSO-d_6) δ 166.17, 149.65, 142.98, 140.08, 139.84, 135.55, 135.14, 129.42, 128.88, 128.27, 127.35, 126.65, 13.44.
HRMS (ESI) calculated for formula $C_{16}H_{15}N_4OS$ ($[M+H]^+$) 311.0963, found 311.0961.

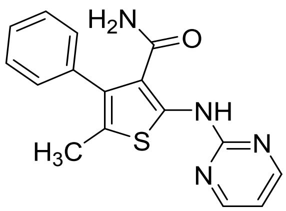

5-methyl-4-phenyl-2-(pyrimidin-2-ylamino)thiophene-3-carboxamide (12f):
Compound was prepared via the general procedure for **Buchwald couplings** using 10.8 mg of 2-chloropyrimidine and was purified using reverse phase HPLC (Gilson 30x100 mm column, 10-60% acetonitrile/ 0.01 aqueous NH_4OH 10 minute run) to give **(12f)** (18 mg, 61% yield) as an off-white solid.
^{1}H NMR (400 MHz, DMSO-d_6) δ 8.62 (d, J = 4.8 Hz, 2H), 7.55 – 7.49 (m, 2H), 7.49 – 7.42 (m, 2H), 7.35 – 7.30 (m, 2H), 7.01 (t, J = 4.8 Hz, 1H), 5.13 (s, 1H), 2.08 (s, 3H).
$^{13}C\{^{1}H\}$ NMR (101 MHz, DMSO-d_6) δ 167.31, 158.30, 156.80, 146.28, 135.68, 133.36, 129.99, 129.01, 128.18, 123.41, 113.70, 112.05, 12.48.
HRMS (ESI) calculated for formula $C_{16}H_{15}N_4OS$ ($[M+H]^+$) 311.0961, found 311.0966.

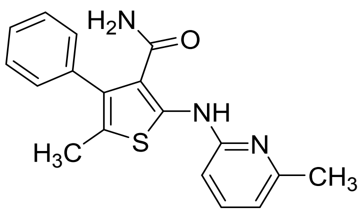

5-methyl-2-((6-methylpyridin-2-yl)amino)-4-phenylthiophene-3-carboxamide (12h):
Compound was prepared via the general procedure for **Buchwald couplings** using 16 mg of 2-bromo-6-methylpyridine and was purified using reverse phase HPLC (Gilson 30x100 mm column, 20-95% acetonitrile/ 0.01 aqueous NH_4OH 10 minute run) to give **(12h)** (14 mg, 47 % yield) as an off-white solid.

^{1}H NMR (400 MHz, DMSO-d_6) δ 7.61 – 7.40 (m, 4H), 7.35 – 7.19 (m, 3H), 6.78 (dd, J = 18.7, 0.7 Hz, 2H), 5.11 (s, 1H), 2.47 (s, 3H), 2.06 (s, 3H).

$^{13}C\{^{1}H\}$ NMR (101 MHz, DMSO-d_6) δ 168.04, 155.80, 151.41, 148.03, 138.78, 136.61, 133.24, 130.45, 129.39, 128.46, 122.58, 115.13, 111.23, 108.40, 24.00, 12.94.

HRMS (ESI) calculated for formula $C_{18}H_{18}N_3OS$ ($[M+H]^+$) 324.1165, found 324.1166.

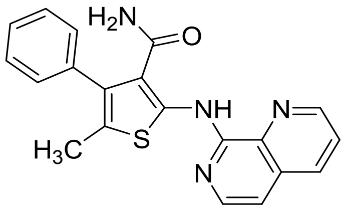

2-((1,7-naphthyridin-8-yl)amino)-5-methyl-4-phenylthiophene-3-carboxamide (12j):

Compound was prepared via the general procedure for **Buchwald couplings** using 14 mg of 8-chloro-1,7-naphthyridine and was purified using reverse phase HPLC (Gilson 30x100 mm column, 30-80% acetonitrile/ 0.01 aqueous NH_4OH 10 minute run) to give **(12j)** (11.4 mg, 37% yield) as a yellow solid.

^{1}H NMR (400 MHz, DMSO-d_6) δ 8.98 (dd, J = 4.3, 1.6 Hz, 1H), 8.36 (dd, J = 8.3, 1.6 Hz, 1H), 8.23 (d, J = 5.8 Hz, 1H), 7.82 (dd, J = 8.3, 4.3 Hz, 1H), 7.56 – 7.50 (m, 2H), 7.49 – 7.43 (m, 1H), 7.39 – 7.20 (m, 4H), 5.18 (s, 1H), 2.13 (s, 3H).

$^{13}C\{^{1}H\}$ NMR (101 MHz, DMSO-d_6) δ 167.60, 150.58, 150.11, 146.04, 141.29, 136.40, 135.62, 133.73, 133.60, 131.85, 130.51, 129.41, 128.52, 126.70, 123.86, 113.50, 112.36, 13.02.

HRMS (ESI) calculated for formula $C_{20}H_{17}N_4OS$ ($[M+H]^+$) 361.1118, found 361.1115.

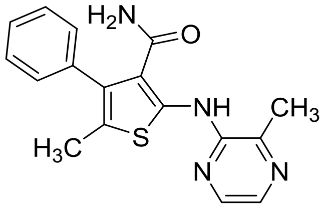

5-methyl-2-((3-methylpyrazin-2-yl)amino)-4-phenylthiophene-3-carboxamide (12l):

Compound was prepared via the general procedure for **Buchwald couplings** using 12 mg of 2-chloro-3-methylpyrazine and was purified using reverse phase HPLC (Gilson 30x100 mm column,

20-75% acetonitrile/ 0.01 aqueous NH_4OH 10 minute run) to give **(12l)** (18.3 mg, 60 % yield) as a yellow solid.

^{1}H NMR (400 MHz, DMSO-d_6) δ 8.17 (d, J = 2.0 Hz, 1H), 7.99 (d, J = 2.9 Hz, 1H), 7.57 – 7.43 (m, 4H), 7.37 – 7.30 (m, 2H), 5.07 (s, 1H), 2.52 (s, 3H), 2.08 (s, 3H).

^{13}C{^{1}H} NMR (101 MHz, DMSO-d_6) δ 167.80, 146.85, 146.52, 141.91, 138.25, 135.72, 133.97, 133.18, 130.00, 129.10, 128.29, 123.04, 111.73, 19.66, 12.44.

HRMS (ESI) calculated for formula $C_{17}H_{17}N_4OS$ ($[M+H]^+$) 325.1118, found 325.1119.

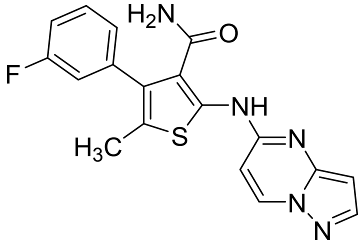

4-(3-fluorophenyl)-5-methyl-2-(pyrazolo[1,5-a]pyrimidin-5-ylamino)thiophene-3-carboxamide (12a):

Compound was prepared via the general procedure for **Buchwald couplings** using 14.4 mg of 5-chloropyrazolo[1,5-a]pyrimidine and was purified using reverse phase HPLC (Gilson 30x100 mm column, 15-70% acetonitrile/ 0.01 aqueous NH_4OH 10 minute run) then via flash chromatography (Teledyne ISCO flash purification system; silica gel column; hexanes:EtOAc; 20-100% EtOAc gradient; liquid loading) to give **(12a)** (20.5 mg, 61 % yield) as a white solid.

^{1}H NMR (400 MHz, DMSO-d_6) δ 11.38 (s, 1H), 8.76 (dd, J = 7.5, 0.9 Hz, 1H), 7.98 (d, J = 2.2 Hz, 1H), 7.74 – 7.47 (m, 1H), 7.42 (bs, 1H), 7.37 – 7.20 (m, 1H), 7.20 – 6.99 (m, 1H), 6.83 (d, J = 7.5 Hz, 1H), 6.31 (dd, J = 2.2, 0.8 Hz, 1H), 5.99 (bs, 1H), 2.18 (s, 3H)

^{13}C{^{1}H} NMR (101 MHz, DMSO-d_6) δ 166.81, 162.01 (d, J_{CF} = 244.5 Hz), 149.75, 146.45, 144.48, 142.15, 137.88 (d, J_{CF} = 8.0 Hz), 136.35, 132.03 (d, J_{CF} = 2.1 Hz), 130.52 (d, J_{CF} = 8.6 Hz), 126.11 (d, J_{CF} = 2.7 Hz), 125.21, 116.74 (d, J_{CF} = 21.3 Hz), 116.50, 114.57 (d, J_{CF} = 20.8 Hz), 100.56, 93.00, 12.64.

HRMS (ESI) calculated for formula $C_{18}H_{15}FN_5OS$ ($[M+H]^+$) 368.0976, found 368.0985.

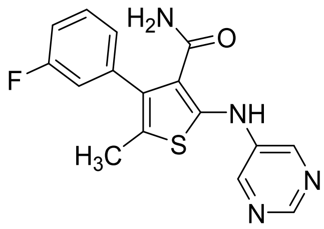

4-(3-fluorophenyl)-5-methyl-2-(pyrimidin-5-ylamino)thiophene-3-carboxamide (12c):
Compound was prepared via the general procedure for **Buchwald couplings** using 14.6 mg of 5-bromopyrimidine and was purified using reverse phase HPLC (Gilson 30x100 mm column, 15-70% acetonitrile/ 0.01 aqueous NH_4OH 10 minute run) then flash chromatography (Teledyne ISCO flash purification system; silica gel column; DCM:MeOH; 0-5% MeOH gradient; liquid loading) to give **(12c)** (18 mg, 59 % yield) as a white solid.
^{1}H NMR (400 MHz, DMSO-d_6) δ 8.72 (s, 1H), 8.62 (s, 1H), 8.42 (s, 2H), 7.50 – 7.42 (m, 1H), 7.30 (bs, 1H), 7.23 – 7.05 (m, 4H), 2.26 (s, 3H)
^{13}C{^{1}H} NMR (101 MHz, DMSO-d_6) δ 165.93, 161.85 (d, J_{CF} = 243.6 Hz), 149.53, 142.74, 140.38, 138.91, 137.44 (d, J_{CF} = 8.2 Hz), 134.27 (d, J_{CF} = 2.1 Hz), 130.38, 130.09 (d, J_{CF} = 8.7 Hz), 128.22, 125.65 (d, J_{CF} = 2.8 Hz), 116.15 (d, J_{CF} = 21.5 Hz), 114.13 (d, J_{CF} = 20.8 Hz), 13.48
HRMS (ESI) calculated for formula $C_{16}H_{14}FN_4OS$ ($[M+H]^+$) 329.0867, found 329.0873.

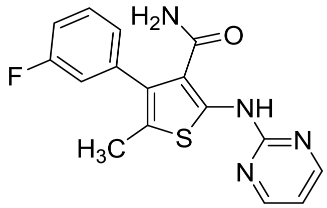

4-(3-fluorophenyl)-5-methyl-2-(pyrimidin-2-ylamino)thiophene-3-carboxamide (12e):
Compound was prepared via the general procedure for **Buchwald couplings** using 10.6 mg of 2-chloropyrimidine and was purified using reverse phase HPLC (Gilson 30x100 mm column, 15-70% acetonitrile/ 0.01 aqueous NH_4OH 10 minute run) to give **(12e)** (13.2 mg, 44 % yield) as a white solid.
^{1}H NMR (400 MHz, DMSO-d_6) δ 8.62 (d, J = 4.8 Hz, 2H), 7.54 (td, J = 8.0, 6.2 Hz, 1H), 7.45 (bs, 1H), 7.25-7.30 (m, 1H), 7.23 – 7.12 (m, 2H), 7.01 (t, J = 4.8 Hz, 1H), 5.33 (bs, 1H), 2.11 (s, 3H).

$^{13}C\{^1H\}$ NMR (101 MHz, DMSO-d_6) δ 167.15, 162.10 (d, J_{CF} = 245.1 Hz), 158.32, 156.83, 145.78, 137.88 (d, J_{CF} = 7.9 Hz), 132.09 (d, J_{CF} = 2.0 Hz), 130.80 (d, J_{CF} = 8.7 Hz), 126.28 (d, J_{CF} = 2.8 Hz), 124.08, 116.99 (d, J_{CF} = 21.2 Hz), 114.93 (d, J_{CF} = 20.8 Hz), 113.73, 112.63, 12.50.
HRMS (ESI) calculated for formula $C_{16}H_{14}FN_4OS$ ($[M+H]^+$) 329.0867, found 329.0868.

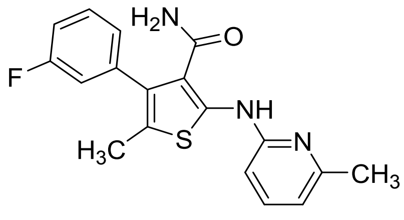

4-(3-fluorophenyl)-5-methyl-2-((6-methylpyridin-2-yl)amino)thiophene-3-carboxamide (12g):

Compound was prepared via the general procedure for **Buchwald couplings** using 15.8 mg of 2-bromo-6-methylpyridine and was purified using reverse phase HPLC (Gilson 30x100 mm column, 15-70% acetonitrile/ 0.01 aqueous NH_4OH 10 minute run) to give **(12g)** (17.7 mg, 56% yield) as a beige glass.
^1H NMR (400 MHz, DMSO-d_6) δ 7.54 (m, 2H), 7.42 – 7.05 (m, 4H), 6.81 (d, J = 8.2 Hz, 1H), 6.75 (d, J = 7.3 Hz, 1H), 5.35 (bs, 1H), 2.46 (s, 3H), 2.09 (s, 3H)
$^{13}C\{^1H\}$ NMR (101 MHz, DMSO-d_6) δ 167.39, 162.07 (d, J_{CF} = 245.0 Hz), 155.30, 151.02, 146.81, 138.38 (d, J_{CF} = 7.9 Hz), 138.29, 131.51 (d, J_{CF} = 2.1 Hz), 130.67 (d, J_{CF} = 8.7 Hz), 126.24 (d, J_{CF} = 2.8 Hz), 122.79, 116.90 (d, J_{CF} = 21.2 Hz), 114.69 (d, J_{CF} = 20.9 Hz), 114.64, 111.50, 107.89, 23.51, 12.48
HRMS (ESI) calculated for formula $C_{18}H_{17}FN_3OS$ ($[M+H]^+$) 342.1071, found 342.1074.

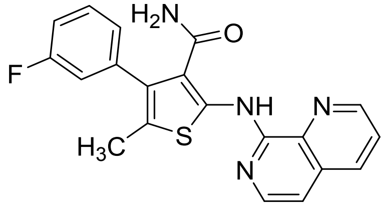

2-((1,7-naphthyridin-8-yl)amino)-4-(3-fluorophenyl)-5-methylthiophene-3-carboxamide (12i):

Compound was prepared via the general procedure for **Buchwald couplings** using 15.2 mg of 8-chloro-1,7-naphthyridine and was purified using reverse phase HPLC (Gilson 30x100 mm column,

15-70% acetonitrile/ 0.01 aqueous NH_4OH 10 minute run) then via flash chromatography (Teledyne ISCO flash purification system; silica gel column; hexanes:EtOAc; 20-100% EtOAc gradient; liquid loading) to give **(12i)** (9.5 mg, 27 % yield) as a yellow solid.

^{1}H NMR (400 MHz, DMSO-d_6) δ 8.98 (dd, J = 4.3, 1.6 Hz, 1H), 8.36 (dd, J = 8.3, 1.6 Hz, 1H), 8.23 (d, J = 5.8 Hz, 1H), 7.82 (dd, J = 8.3, 4.3 Hz, 1H), 7.55 (m, 1H), 7.40 – 7.12 (m, 4H), 2.16 (s, 3H).

^{13}C{^{1}H} NMR (101 MHz, DMSO-d_6) δ 167.02, 162.08 (d, J_{CF} = 245.0 Hz), 150.13, 149.60, 145.06, 140.81, 138.14 (d, J_{CF} = 8.0 Hz), 135.17, 133.19, 131.84 (d, J_{CF} = 2.0 Hz), 131.38, 130.71 (d, J_{CF} = 8.7 Hz), 126.33 (d, v = 2.7 Hz), 126.26, 123.98, 117.01 (d, v = 21.2 Hz), 114.79 (d, J_{CF} = 20.8 Hz), 113.48, 111.93, 12.55.

HRMS (ESI) calculated for formula $C_{20}H_{16}FN_4OS$ ([M+H]$^+$) 379.1023, found 379.1028.

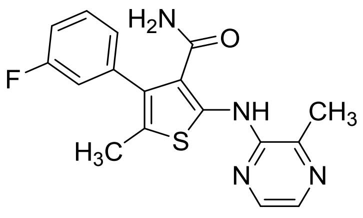

4-(3-fluorophenyl)-5-methyl-2-((3-methylpyrazin-2-yl)amino)thiophene-3-carboxamide (12k):

Compound was prepared via the general procedure for **Buchwald couplings** using 11.8 mg of 2-chloro-3-methylpyrazine and was purified using reverse phase HPLC (Gilson 30x100 mm column, 15-70% acetonitrile/ 0.01 aqueous NH_4OH 10 minute run) to give **(12k)** (12.4 mg, 39 % yield) as a yellow solid.

^{1}H NMR (400 MHz, DMSO-d_6) δ 8.17 (d, J = 2.9 Hz, 1H), 7.98 (d, J = 2.9 Hz, 1H), 7.56 (m, 1H), 7.46 (bs, 1H), 7.36 – 7.26 (m, 1H), 7.25 – 7.06 (m, 2H), 5.22 (bs, 1H), 2.51 (s, 3H), 2.11 (s, 3H).

^{13}C{^{1}H} NMR (101 MHz, DMSO-d_6) δ 167.66, 162.15 (d, J_{CF} = 245.4 Hz), 146.53 (d, J_{CF} = 6.8 Hz), 141.88, 138.23, 137.93 (d, J_{CF} = 8.0 Hz), 134.02, 131.87 (d, J_{CF} = 2.1 Hz), 130.93 (d, J_{CF} = 8.6 Hz), 126.31 (d, J_{CF} = 2.8 Hz), 123.64, 117.04 (d, J_{CF} = 21.2 Hz), 115.09 (d, J_{CF} = 20.8 Hz), 111.99, 19.63, 12.45.

HRMS (ESI) calculated for formula $C_{17}H_{16}FN_4OS$ ([M+H]$^+$) 343.1023, found 343.1028.

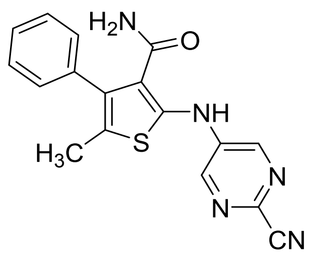

2-((2-cyanopyrimidin-5-yl)amino)-5-methyl-4-phenylthiophene-3-carboxamide (13a):

Compound was prepared via the general procedure for **Buchwald couplings** using 15.4 mg of 5-bromopyrimidine-2-carbonitrile and was purified using flash chromatography (Teledyne ISCO flash purification system; silica gel column; hexanes:EtOAc; 0-60% EtOAc gradient; liquid loading) then reverse phase HPLC (Gilson 30x50 mm column, 10-70% acetonitrile/ 0.01 aqueous NH_4OH 6 minute run) to give **(13a)** (18 mg, 64 % yield) as a yellow solid.

^{1}H NMR (400 MHz, DMSO-d_6) δ 9.37 (s, 1H), 8.42 (s, 2H), 7.46 – 7.39 (m, 2H), 7.38 – 7.27 (m, 5H), 2.29 (s, 3H).

^{13}C{^{1}H} NMR (101 MHz, DMSO-d_6) δ 165.76, 142.86, 142.41, 135.86, 134.75, 134.25, 134.11, 132.46, 130.37, 129.32, 128.17, 127.30, 116.83, 13.66.

HRMS (ESI) calculated for formula $C_{17}H_{14}N_5OS$ ($[M+H]^+$) 336.0914, found 336.0915.

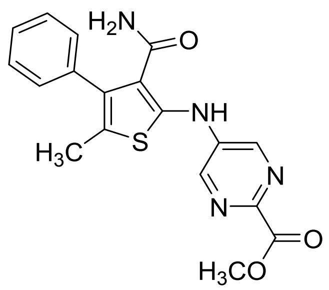

methyl 5-((3-carbamoyl-5-methyl-4-phenylthiophen-2-yl)amino)pyrimidine-2-carboxylate (13c):

Compound was prepared via the general procedure for **Buchwald couplings** using 18 mg of methyl 5-bromopyrimidine-2-carboxylate and was purified using reverse phase HPLC (Gilson 30x100 mm column, 10-65% acetonitrile/ 0.01 aqueous NH_4OH 10 minute run) to give **(13c)** (6 mg, 20 % yield) as an off-white solid.

^{1}H NMR (400 MHz, DMSO-d_6) δ 8.46 (s, 2H), 7.47 – 7.38 (m, 2H), 7.38 – 7.32 (m, 3H), 7.25 (d, J = 27.2 Hz, 2H), 3.84 (s, 3H), 2.28 (s, 3H).

$^{13}C\{^1H\}$ NMR (101 MHz, DMSO-d_6) δ 166.35, 163.78, 146.66, 142.23, 136.53, 136.25, 135.36, 132.92, 129.82, 129.60, 128.66, 127.76, 52.74, 14.07.

HRMS (ESI) calculated for formula $C_{18}H_{17}N_4O_3S$ ($[M+H]^+$) 369.1016, found 369.1016.

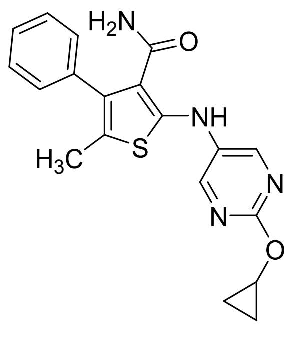

2-((2-cyclopropoxypyrimidin-5-yl)amino)-5-methyl-4-phenylthiophene-3-carboxamide (13e):

Compound was prepared via the general procedure for **Buchwald couplings** using 18 mg of 5-bromo-2-cyclopropoxypyrimidine and was purified using flash chromatography (Teledyne ISCO flash purification system; silica gel column; hexanes:EtOAc; 0-60% EtOAc gradient; liquid loading) then reverse phase HPLC (Gilson 30x50 mm column, 20-75% acetonitrile/ 0.01 aqueous NH_4OH 6 minute run) to give **(13e)** (18 mg, 59 % yield) as an off-white solid.

^1H NMR (400 MHz, DMSO-d_6) δ 9.28 (s, 1H), 8.49 (s, 2H), 7.51 – 7.35 (m, 3H), 7.35 – 7.27 (m, 2H), 7.19 (s, 1H), 5.96 (s, 1H), 4.28 – 4.19 (m, 1H), 2.09 (s, 3H), 0.81 – 0.66 (m, 4H).

$^{13}C\{^1H\}$ NMR (101 MHz, DMSO-d_6) δ 166.55, 160.93, 149.73, 148.52, 135.46, 135.27, 134.04, 129.58, 128.57, 127.69, 121.15, 119.17, 51.05, 13.03, 5.56.

HRMS (ESI) calculated for formula $C_{19}H_{19}N_4O_2S$ ($[M+H]^+$) 367.1223, found 367.1223.

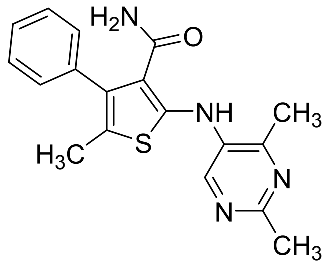

2-((2,4-dimethylpyrimidin-5-yl)amino)-5-methyl-4-phenylthiophene-3-carboxamide (13g):

Compound was prepared via the general procedure for **Buchwald couplings** using 15.6 mg of 5-bromo-2,4-dimethylpyrimidine and was purified using reverse phase HPLC (Gilson 30x100 mm column, 20-65% acetonitrile/ 0.01 aqueous NH_4OH 10 minute run) to give **(13g)** (13.8 mg, 49 % yield) as a yellow solid.

H NMR (400 MHz, DMSO-d_6) δ 9.79 (s, 1H), 8.49 (s, 1H), 7.52 – 7.45 (m, 2H), 7.45 – 7.39 (m, 1H), 7.36 – 7.28 (m, 2H), 7.24 (s, 1H), 5.65 (s, 1H), 2.53 (s, 3H), 2.43 (s, 3H), 2.09 (s, 3H).

$^{13}C\{^1H\}$ NMR (101 MHz, DMSO-d_6) δ 166.81, 159.97, 156.82, 149.50, 144.67, 135.44, 135.11, 133.75, 129.69, 128.73, 127.91, 120.85, 116.75, 24.77, 20.15, 12.90.

HRMS (ESI) calculated for formula $C_{18}H_{19}N_4OS$ ($[M+H]^+$) 339.1274, found 339.1275.

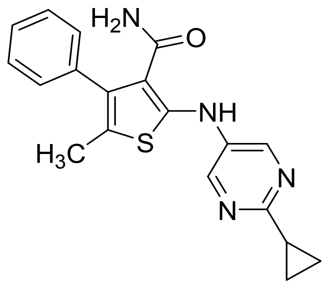

2-((2-cyclopropylpyrimidin-5-yl)amino)-5-methyl-4-phenylthiophene-3-carboxamide (13i):
Compound was prepared via the general procedure for **Buchwald couplings** using 17 mg of 5-bromo-2-cyclopropylpyrimidine and was purified using reverse phase HPLC (Gilson 30x100 mm column, 25-70% acetonitrile/ 0.01 aqueous NH_4OH 10 minute run) to give **(13i)** (16.5 mg, 55 % yield) as an off-white solid.

^1H NMR (400 MHz, DMSO-d_6) δ 9.17 (s, 1H), 8.43 (s, 2H), 7.51 – 7.41 (m, 2H), 7.41 – 7.35 (m, 1H), 7.34 – 7.28 (m, 2H), 7.22 (s, 1H), 6.34 (s, 1H), 2.15 (s, 3H), 2.14 – 2.08 (m, 1H), 1.00 – 0.86 (m, 4H).

$^{13}C\{^1H\}$ NMR (101 MHz, DMSO-d_6) δ 166.39, 162.81, 144.96, 144.35, 136.57, 135.35, 135.33, 129.51, 128.43, 127.53, 123.51, 123.38, 17.16, 13.20, 9.52.

HRMS (ESI) calculated for formula $C_{19}H_{18}N_4OS$ ($[M+H]^+$) 351.1274, found 351.1274.

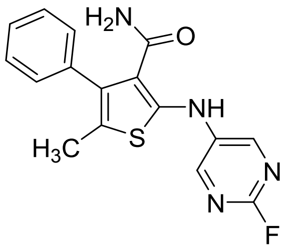

2-((2-fluoropyrimidin-5-yl)amino)-5-methyl-4-phenylthiophene-3-carboxamide (13m):

Compound was prepared via the general procedure for **Buchwald couplings** using 15 mg of 5-bromo-2-fluoropyrimidine and was purified using reverse phase HPLC (Gilson 30x100 mm column, 15-70% acetonitrile/ 0.01 aqueous NH_4OH 10 minute run) then via flash chromatography (Teledyne ISCO flash purification system; silica gel column; hexanes:EtOAc; 20-100% EtOAc gradient; liquid loading) to give **(13m)** (2.7 mg, 10 % yield) as a beige solid.

^{1}H NMR (400 MHz, DMSO-d_6) δ 9.24 (s, 1H), 8.69 (s, 2H), 7.93 – 7.68 (m, 2H), 7.70 – 7.59 (m, 3H), 7.55 (bs, 1H), 7.09 (bs, 1H), 2.50 (s, 3H).

^{13}C{^{1}H} NMR (101 MHz, DMSO-d_6) δ 166.24, 156.33 (d, J_{CF} = 207.8 Hz), 147.10 (d, J_{CF} = 12.0 Hz), 140.84, 139.11 (d, J_{CF} = 5.1 Hz), 135.55, 135.13, 129.43, 128.32, 128.21, 127.42, 126.28, 13.42.

HRMS (ESI) calculated for formula $C_{16}H_{14}FN_4OS$ ($[M+H]^+$) 329.0867, found 329.0869.

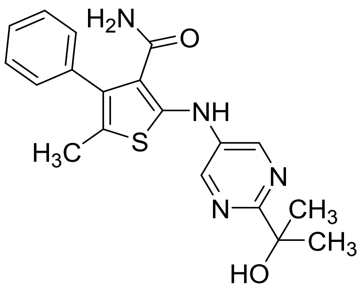

2-((2-(2-hydroxypropan-2-yl)pyrimidin-5-yl)amino)-5-methyl-4-phenylthiophene-3-carboxamide (13k):

Compound was prepared via the general procedure **for Buchwald couplings** using 18 mg of 2-(5-bromopyrimidin-2-yl)propan-2-ol and was purified using reverse phase HPLC (Gilson 30x100 mm column, 15-65% acetonitrile/ 0.01 aqueous NH_4OH 10 minute run) to give **(13k)** (6.8 mg, 22 % yield) as an off-white solid.

^{1}H NMR (400 MHz, DMSO-d_6) δ 9.09 (s, 1H), 8.51 (s, 2H), 7.49 – 7.29 (m, 5H), 7.25 (s, 1H), 6.62 (s, 1H), 4.90 (s, 1H), 2.19 (s, 3H), 1.46 (s, 6H).

$^{13}C\{^{1}H\}$ NMR (101 MHz, DMSO-d_6) δ 166.28, 165.54, 143.49, 141.98, 137.56, 135.47, 135.23, 129.47, 128.35, 127.45, 126.31, 125.13, 72.27, 29.80, 13.33.

HRMS (ESI) calculated for formula $C_{19}H_{21}N_4O_2S$ ($[M+H]^+$) 369.1380, found 369.1380.

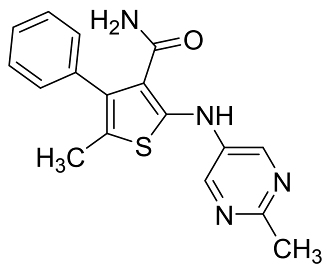

5-methyl-2-((2-methylpyrimidin-5-yl)amino)-4-phenylthiophene-3-carboxamide (13o):

Compound was prepared via the general procedure for **Buchwald couplings** using 15 mg of 5-bromo-2-methylpyrimidine and was purified using reverse phase HPLC (Gilson 30x100 mm column, 20-70% acetonitrile/ 0.01 aqueous NH_4OH 10 minute run) then by flash chromatography (Teledyne ISCO flash purification system; silica gel column; hexanes:EtOAc; 0-100% EtOAc gradient; liquid loading) to give **(13o)** (9 mg, 32 % yield) as a white solid.

^{1}H NMR (400 MHz, DMSO-d_6) δ 9.08 (s, 1H), 8.44 (s, 2H), 7.48 – 7.41 (m, 2H), 7.40 – 7.29 (m, 3H), 7.24 (s, 1H), 6.50 (s, 1H), 2.52 (s, 3H), 2.17 (s, 3H).

$^{13}C\{^{1}H\}$ NMR (101 MHz, DMSO-d_6) δ 166.34, 158.64, 144.29, 143.03, 136.90, 135.41, 135.27, 129.49, 128.38, 127.48, 125.00, 124.43, 24.60, 13.28.

HRMS (ESI) calculated for formula $C_{17}H_{17}N_4OS$ ($[M+H]^+$) 325.1118, found 325.1118.

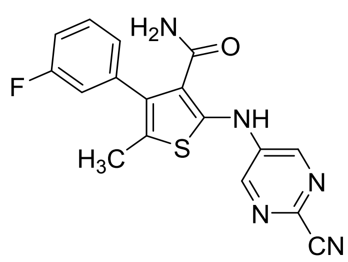

2-((2-cyanopyrimidin-5-yl)amino)-4-(3-fluorophenyl)-5-methylthiophene-3-carboxamide (13b):

Compound was prepared via the general procedure for **Buchwald couplings** using 16.9 mg of 5-bromopyrimidine-2-carbonitrile and was purified using reverse phase HPLC (Gilson 30x100 mm column, 15-70% acetonitrile/ 0.01 aqueous NH_4OH 10 minute run) to give **(13b)** (16.8 mg, 52 % yield) as a yellow solid.

^{1}H NMR (400 MHz, DMSO-d_6) δ 9.33 (s, 1H), 8.40 (s, 2H), 7.53 – 7.41 (m, 2H), 7.35 (s, 1H), 7.23 – 7.13 (m, 3H), 2.31 (s, 3H)

^{13}C{^{1}H} NMR (101 MHz, DMSO-d_6) δ 165.57, 161.82 (d, J_{CF} = 243.4 Hz), 142.92, 142.40, 137.07 (d, J_{CF} = 8.2 Hz), 134.49 (d, J_{CF} = 2.1 Hz), 134.30, 134.25, 132.51, 131.33, 130.09 (d, J_{CF} = 8.6 Hz), 125.59 (d, J_{CF} = 2.7 Hz), 116.83, 116.07 (d, J_{CF} = 21.6 Hz), 114.18 (d, J_{CF} = 20.8 Hz), 13.64

HRMS (ESI) calculated for formula $C_{17}H_{13}FN_5OS$ ($[M+H]^+$) 354.0819, found 354.0821.

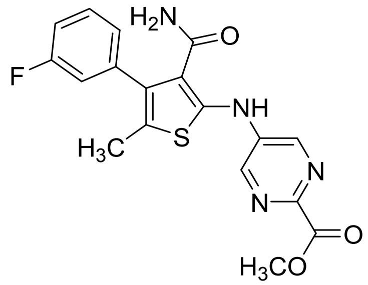

methyl 5-((3-carbamoyl-4-(3-fluorophenyl)-5-methylthiophen-2-yl)amino)pyrimidine-2-carboxylate (13d):

Compound was prepared via the general procedure for **Buchwald couplings** using 20 mg of methyl 5-bromopyrimidine-2-carboxylate and was purified via flash chromatography (Teledyne ISCO flash purification system; silica gel column; hexanes:EtOAc; 20-100% EtOAc gradient; liquid loading) then using reverse phase HPLC (Gilson 30x100 mm column, 15-70% acetonitrile/ 0.01 aqueous NH_4OH 10 minute run) to give **(13d)** (9 mg, 25 % yield) as a yellow solid.

^{1}H NMR (400 MHz, DMSO-d_6) δ 9.14 (s, 1H), 8.44 (s, 2H), 7.66 – 7.42 (m, 1H), 7.37 (bs, 1H), 7.34 (bs, 1H), 7.28 – 7.11 (m, 3H), 3.84 (s, 3H), 2.30 (s, 3H).

^{13}C{^{1}H} NMR (101 MHz, DMSO-d_6) δ 165.68, 163.32, 161.83 (d, J_{CF} = 243.4 Hz), 146.21, 141.87, 141.70, 137.19 (d, J_{CF} = 8.2 Hz), 135.75, 134.43 (d, J_{CF} = 2.1 Hz), 133.04, 130.27, 130.08 (d, J_{CF} = 8.6 Hz), 125.61 (d, J_{CF} = 2.8 Hz), 116.09 (d, J_{CF} = 21.6 Hz), 114.14 (d, J_{CF} = 20.8 Hz), 52.28, 13.60.

HRMS (ESI) calculated for formula $C_{18}H_{16}FN_4O_3S$ ($[M+H]^+$) 387.0922, found 387.0922.

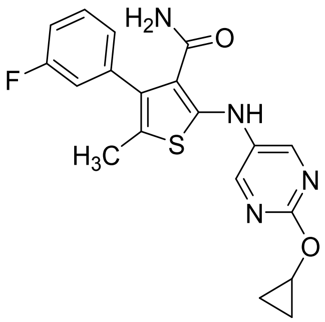

2-((2-cyclopropoxypyrimidin-5-yl)amino)-4-(3-fluorophenyl)-5-methylthiophene-3-carboxamide (13f):

Compound was prepared via the general procedure for **Buchwald couplings** using 19.8 mg of 5-bromo-2-cyclopropoxypyrimidine and was purified using reverse phase HPLC (Gilson 30x100 mm column, 15-70% acetonitrile/ 0.01 aqueous NH_4OH 10 minute run) to give **(13f)** (4 mg, 11 % yield) as a beige glass.

^{1}H NMR (400 MHz, DMSO-d_6) δ 8.91 (s, 1H), 8.43 (s, 2H), 7.45-7.51 (m, 1H), 7.38 – 7.07 (m, 4H), 6.43 (bs, 1H), 4.63 – 3.86 (m, 1H), 2.15 (s, 3H), 1.28 – 0.29 (m, 4H).

^{13}C{^{1}H} NMR (101 MHz, DMSO-d_6) δ 166.25, 161.91 (d, J_{CF} = 244.8 Hz), 150.48, 148.94, 146.41, 137.70 (d, J_{CF} = 8.0 Hz), 134.59, 134.07 (d, J_{CF} = 2.0 Hz), 130.30 (d, J_{CF} = 8.7 Hz), 125.78 (d, J_{CF} = 2.7 Hz), 123.22, 121.99, 116.35 (d, J_{CF} = 21.4 Hz), 114.35 (d, J_{CF} = 20.8 Hz), 50.99, 13.13, 5.56.

HRMS (ESI) calculated for formula $C_{19}H_{18}FN_4O_2S$ ([M+H]$^+$) 385.1129, found 385.1131.

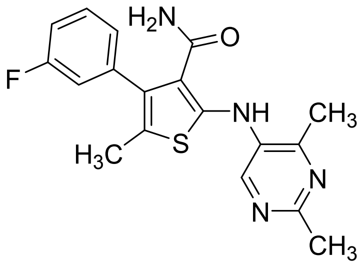

2-((2,4-dimethylpyrimidin-5-yl)amino)-4-(3-fluorophenyl)-5-methylthiophene-3-carboxamide (13h):

Compound was prepared via the general procedure for **Buchwald couplings** using 17.2 mg of 5-bromo-2,4-dimethylpyrimidine and was purified using reverse phase HPLC (Gilson 30x100 mm column, 15-70% acetonitrile/ 0.01 aqueous NH_4OH 10 minute run) then via flash

chromatography (Teledyne ISCO flash purification system; silica gel column; hexanes:EtOAc; 20-100% EtOAc gradient; liquid loading) to give **(13h)** (17.3 mg, 53 % yield) as a white solid.

^{1}H NMR (400 MHz, DMSO-d_6) δ 9.26 (s, 1H), 8.38 (s, 1H), 7.50 (td, J = 8.1, 6.2 Hz, 1H), 7.20-7.26 (m, 1H), 7.19 – 7.07 (m, 2H), 6.06 (bs, 1H), 2.51 (s, 3H), 2.42 (s, 3H), 2.13 (s, 3H).

^{13}C{^{1}H} NMR (101 MHz, DMSO-d_6) δ 166.44, 161.97 (d, J_{CF} = 244.4 Hz), 159.70, 156.38, 147.66, 144.43, 137.68 (d, J_{CF} = 8.1 Hz), 134.25, 133.96 (d, J_{CF} = 2.0 Hz), 130.46 (d, J_{CF} = 8.6 Hz), 125.90 (d, J_{CF} = 2.8 Hz), 122.89, 119.56, 116.53 (d, J_{CF} = 21.5 Hz), 114.57 (d, J_{CF} = 20.8 Hz), 24.73, 20.16, 13.01.

HRMS (ESI) calculated for formula $C_{18}H_{18}FN_4OS$ ([M+H]$^+$) 357.1180, found 357.1183.

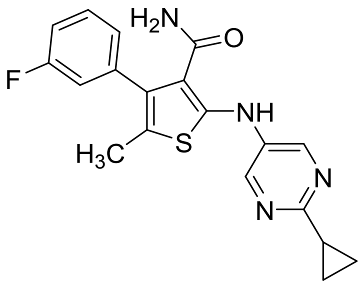

2-((2-cyclopropylpyrimidin-5-yl)amino)-4-(3-fluorophenyl)-5-methylthiophene-3-carboxamide (13j):

Compound was prepared via the general procedure for **Buchwald couplings** using 18.3 mg of 5-bromo-2-cyclopropylpyrimidine and was purified using reverse phase HPLC (Gilson 30x100 mm column, 15-70% acetonitrile/ 0.01 aqueous NH_4OH 10 minute run) to give **(13j)** (10 mg, 29 % yield) as beige glass.

^{1}H NMR (400 MHz, DMSO-d_6) δ 8.82 (s, 1H), 8.37 (s, 2H), 7.47 (m, 1H), 7.26 (s, 1H), 7.23 – 7.12 (m, 3H), 6.75 (bs, 1H), 2.20 (bs, 3H), 2.11 (m, 1H), 0.94 (m, 2H), 0.89 (m, 2H)

^{13}C{^{1}H} NMR (101 MHz, DMSO-d_6) δ 166.09, 162.48, 161.87 (d, J_{CF} = 243.7 Hz), 144.45, 142.60, 137.59 (d, J_{CF} = 8.1 Hz), 137.06, 134.13 (d, J_{CF} = 2.0 Hz), 130.18 (d, J_{CF} = 8.6 Hz), 125.92, 125.72 (d J_{CF} = 2.6 Hz), 125.49, 116.25 (d, J_{CF} = 21.4 Hz), 114.22 (d, J_{CF} = 20.8 Hz), 17.12, 13.29, 9.45

HRMS (ESI) calculated for formula $C_{19}H_{18}FN_4O_2S$ ([M+H]$^+$) 369.1180, found 369.1183.

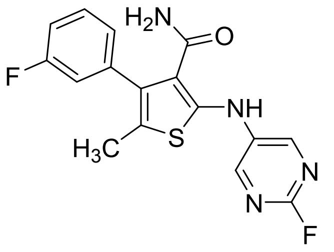

4-(3-fluorophenyl)-2-((2-fluoropyrimidin-5-yl)amino)-5-methylthiophene-3-carboxamide (13n):

Compound was prepared via the general procedure for **Buchwald couplings** using 16.3 mg of 5-bromo-2-fluoropyrimidine and was purified using reverse phase HPLC (Gilson 30x100 mm column, 15-70% acetonitrile/ 0.01 aqueous NH_4OH 10 minute run) then via flash chromatography (Teledyne ISCO flash purification system; silica gel column; hexanes:EtOAc; 20-100% EtOAc gradient; liquid loading) to give **(13n)** (8.8 mg, 28 % yield) as a white solid.

^{1}H NMR (400 MHz, DMSO-d_6) δ 8.75 (s, 1H), 8.35 (d, J = 0.8 Hz, 2H), 7.44-7.50 (m, 1H), 7.30 (s, 1H), 7.23 – 7.12 (m, 3H), 7.09 (s, 1H), 2.25 (s, 3H)

^{13}C{^{1}H} NMR (101 MHz, DMSO-d_6) δ 165.97, 161.85 (d, J_{CF} = 243.5 Hz), 156.23 (d, J_{CF} = 207.5 Hz), 146.67 (d, J_{CF} = 11.9 Hz), 139.69, 139.41 (d, J_{CF} = 5.1 Hz), 137.39 (d, J_{CF} = 8.2 Hz), 134.24 (d, J_{CF} = 2.1 Hz), 130.12 (d, J_{CF} = 8.6 Hz), 129.85, 127.91, 125.65 (d, J_{CF} = 2.8 Hz), 116.14 (d, J_{CF} = 21.4 Hz), 114.17 (d, J = 20.8 Hz), 13.46

HRMS (ESI) calculated for formula $C_{16}H_{13}F_2N_4OS$ ($[M+H]^+$) 347.0773, found 347.0777.

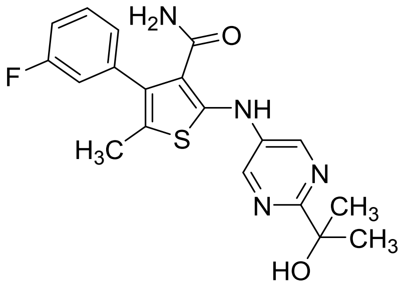

4-(3-fluorophenyl)-2-((2-(2-hydroxypropan-2-yl)pyrimidin-5-yl)amino)-5-methylthiophene-3-carboxamide (13l; VU6053371):

Compound was prepared via the general procedure for **Buchwald couplings** using 20 mg of 2-(5-bromopyrimidin-2-yl)propan-2-ol and was purified using reverse phase HPLC (Gilson 30x100 mm column, 15-70% acetonitrile/ 0.01 aqueous NH_4OH 10 minute run) to give **(13l; VU6053371)** (3 mg, 8 % yield) as an off-white solid.

^{1}H NMR (400 MHz, DMSO-d_6) δ 8.81 (s, 1H), 8.45 (s, 2H), 7.47 (td, J = 8.1, 6.1 Hz, 1H), 7.30 (bs, 1H), 7.23 – 7.13 (m, 3H), 6.97 (bs, 1H), 4.89 (s, 1H), 2.23 (s, 3H), 1.46 (s, 6H)

$^{13}C\{^{1}H\}$ NMR (101 MHz, DMSO-d_6) δ 166.00, 165.31, 161.86 (d, J_{CF} = 243.6 Hz), 143.10, 140.59, 137.96, 137.51 (d, J_{CF} = 8.1 Hz), 134.21 (d, J_{CF} = 2.1 Hz), 130.13 (d, J_{CF} = 8.6 Hz), 128.39, 126.94, 125.68 (d, J_{CF} = 2.8 Hz), 116.20 (d, J_{CF} = 21.3 Hz), 114.18 (d, J_{CF} = 20.8 Hz), 72.24, 29.81, 13.40

HRMS (ESI) calculated for formula $C_{19}H_{20}FN_4O_2S$ ([M+H]$^+$) 387.1286, found 387.1289.

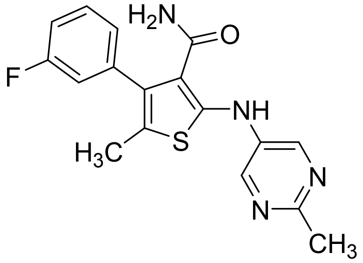

4-(3-fluorophenyl)-5-methyl-2-((2-methylpyrimidin-5-yl)amino)thiophene-3-carboxamide (13p):

Compound was prepared via the general procedure for **Buchwald couplings** using 15.9 mg of 5-bromo-2-methylpyrimidine and was purified using reverse phase HPLC (Gilson 30x100 mm column, 15-70% acetonitrile/ 0.01 aqueous NH_4OH 10 minute run) to give **(13p)** (10.3 mg, 33 % yield) as a white solid.

^{1}H NMR (400 MHz, DMSO-d_6) δ 8.76 (s, 1H), 8.38 (s, 2H), 7.51 – 7.41 (m, 1H), 7.35 – 7.23 (m, 1H), 7.23 – 7.13 (m, 3H), 6.87 (s, 1H), 2.51 (s, 3H), 2.22 (s, 3H).

$^{13}C\{^{1}H\}$ NMR (101 MHz, DMSO-d_6) δ 166.05, 161.86 (d, J_{CF} = 243.6 Hz), 158.36, 143.87, 141.47, 137.54 (d, J_{CF} = 8.2 Hz), 137.34, 134.17 (d, J_{CF} = 2.1 Hz), 130.15 (d, J_{CF} = 8.5 Hz), 127.31, 126.32, 125.69 (d, J_{CF} = 2.7 Hz), 116.21 (d, J_{CF} = 21.5 Hz), 114.19 (d, J_{CF} = 20.8 Hz), 24.57, 13.36.

HRMS (ESI) calculated for formula $C_{17}H_{16}FN_4OS$ ([M+H]$^+$) 343.1023, found 343.1025.

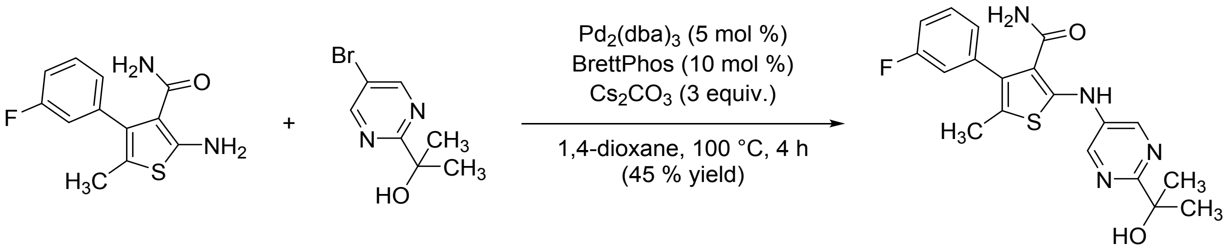

Large scale conditions for 13l (VU6053371):

A round bottom flask was charged with 2-(5-bromopyrimidin-2-yl)propan-2-ol (1.500 g, 6.91 mmol, 1.0 equiv.) , 2-amino-4-(3-fluorophenyl)-5-methylthiophene-3-carboxamide (3.000 g, 11.99 mmol, 1.7 equiv.), potassium carbonate (2.907 g, 20.73 mmol, 3.0 equiv.), tris(dibenzylideneacetone)dipalladium (0.316 g, 0.35 mmol, 5 mol %) , Brettphos (0.371 g, 0.69 mmol, 10 mol %), and 1,4-Dioxane (70mL). The reaction mixture was sparged with nitrogen for 15 minutes before the reaction vial was sealed and heated to 100 °C for 4 hours. Upon cooling to room temperature, the crude reaction was diluted with ethyl acetate, filtered through a pad of celite, concentrated and purified via flash chromatography (Purification #1: Teledyne ISCO flash purification system; silica gel column; DCM : MeOH; 0 - 2.5% MeOH) then again via an isocratic elution (Teledyne ISCO flash purification system; silica gel column; hexane : EtOAc; 55% EtOAc isocratic) to afford **13l (VU6053371)** (1.210 g, 45% yield) as a beige solid.

In vitro molecular pharmacology methods (cAMP)

Description of $mGlu_3$ Potentiator Screen (cAMP)

The activation of the Gi-coupled $mGlu_3$ causes an inhibition of intracellular cAMP after stimulation with Forskolin, which can be quantified by use of a suitable assay kit and an adequate plate reader. This technique is used to characterize pharmacological effects of the $mGlu_3$ potentiators by the use of an appropriate cell line.

Materials:

Barcode labelled CELLCOAT® 384 Poly-D-Lysine coated white microplates with lid (Corning)

Nutrient Mixture F12 Ham's w/o Glutamine (Sigma)

1 M HEPES (Sigma)

Forskolin (Sigma)

IBMX (Sigma)

L-Glutamic acid (Sigma)

FBS (GIBCO-Invitrogen)

HBSS 10X with Ca/Mg (GIBCO-Invitrogen)

BSA Fraction V (Protease free) (Serva)

cAMP Gi Kit (Cisbio Bioassays and lysis buffer (Cisbio Bioassays).

Cell line:

The "CHO K1 hGRM cell line" which is the CHO K1 cell line overexpressing the human metabotropic Glutamate 3 receptor (Entrez reference gene ID: 2931; NP_000831.2; NM_000840.2) was purchased from DiscovereX (cAMP Hunter CHO-K1 GRM3 Cell Line; CatNo. 95-0137C2). The CHO K1 hGRM cell line was expanded to larger cell numbers and

the cells were obtained frozen in cryo vials with 1-20 million cells/vial and stored until use at -150°C. The viability of the cells after thawing was >90%.

Method:

The CHO K1 hGRM3 cells are thawed at 37°C and immediately diluted with cell culture medium. After centrifugation (5 minutes at 900 rpm; Heraeus Multifuge X1R), the cell pellet is re-suspended in medium and then distributed from a stirred spinner flask into the wells of columns 1 to 23 of the assay plates (5.000 cells/well; 25 µL/well). The wells of column 24 are left empty to add cAMP for a standard curve later on in the course of the assay. The plates are incubated for one hour at room temperature before they are incubated for 48 hours at 37°C/5% CO_2. After washing the cells in the plate three times with 80 µL HBSS/HEPES buffer (10 µL buffer remaining in the wells after washing), 5 µL per well of compounds diluted in HBSS/HEPES buffer containing 0.2% BSA (final concentration: 0.1%) and 1 mM IBMX (final concentration: 0.5 mM) are added to the wells in columns 1 to 22 of the assay plate. The plates are incubated for 15 minutes at room temperature. Thereafter 5 µL per well of L-Glutamic acid (final concentration: 50-100 nM), forskolin (final concentration: 0.03 µM) and 1 mM IBMX (final concentration: 0.5 mM) dissolved in HBSS/HEPES buffer containing 0.2% BSA (final concentration: 0.1%) are added to the assay plate (final DMSO concentration: 1%). The wells of columns 23 and 24 are used either for the positive and the negative controls or for the cAMP standard curve (for details see below). The assay plate is incubated for 30 minutes at room temperature. Then 5 µL per well of Anti-cAMP-Antibody-d2 solution and 5 µL per well of cAMP-Europium Cryptate dilution are added to all wells of the plate and the plate is incubated another 60 minutes light protected at room temperature. The emissions at 615 nm and 665 nm (Excitation wavelength: 320 nm) are measured on the EnVision™ reader (PerkinElmer). The

ratio between the emission at 665 nm and 615 is calculated by the reader.

Cell culture medium:

Nutrient Mixture F12 Ham's w/o Glutamine; 10% FBS

HBSS/Hepes buffer:

HBSS1x, 20 mM HEPES pH 7.4

Anti-cAMP-Antibody-d2 (cAMP Gi kit):

Prepared by 1:50 dilution of stock with lysis buffer (cAMP Gi kit)

cAMP-Europium Cryptate (cAMP Gi kit):

Prepared by 1:50 dilution of stock with lysis buffer (cAMP Gi kit)

cAMP standard:

The cAMP standard is prepared by diluting the cAMP stock solution with HBSS/Hepes buffer: 5 µL/well of the cAMP dilutions (in HBSS/Hepes buffer containing 1 mM IBMX and 0.2% BSA - final concentration: 0.5 mM IBMX and 0.1% BSA) are added to 10 µL/well HBSS/Hepes buffer plus 5 µL/well 4% DMSO in HBSS/Hepes containing 0.2% BSA (final DMSO concentration: 1%) in the wells of column 24 of the assay plate. The final cAMP concentrations in the assay plate are: 0, 0.17, 0.69, 2.78, 11.1, 44.5, 178, and 712 nM (two wells/cAMP concentration).

Negative and positive control:

Each assay microtiter plate contains as negative controls 8 wells in column 23 with vehicle controls instead of compound (100 % CTL; 50-100 nM L-Glutamic acid + 0.03 µM forskolin + 0.5 mM IBMX + 1% DMSO); and 8 wells in column 23 as positive control (200% CTL; 100 µM L-Glutamic acid + 0.03 µM forskolin + 0.5 mM IBMX + 1% DMSO).

Calculation:

The analysis of the data is performed by the calculation of the ratio between the emission at 665 nm and the emission at 615 nm (Em665/Em615 ratio). Thereafter the signals of the compounds were normalized using the positive and negative controls by the following formula:

PoC = 100 + 100 x ((Negative Control - Signal Sample) / (Negative Control - Positive Control))

A potentiator of $mGlu_3$ receptor will give values between 100 %CTL (no activation) and 200%CTL (complete activation) after taking the variability of the assay into account. Values far outside this dynamic range of the assay are either due to compound/sample specific physicochemical properties (e.g. solubility, light absorbance, quenching, reactivity) which caninterfere with the assay technology or due to the fact that these compounds may act as a activators of the ion channel.

In vitro molecular pharmacology methods (GIRK)

$mGlu_3$ Thallium Flux Assay

Human embryonic kidney 293 (HEK293) cell lines stably co-expressing human or rat $mGlu_3$ and G protein-coupled inwardly rectifying potassium (GIRK) channels were maintained in growth medium containing DMEM/F12 containing 10% FBS, 20 mM HEPES, 1 mM sodium pyruvate, 2 mM L-glutamine, non-essential amino acids mixture, 100 units/ml penicillin/streptomycin, 700 µg/mL G418, and 0.6 µg/mL puromycin.

The day before the assay, $mGlu_3$ /GIRK cells were plated into 384 well, black-walled, clear-bottom poly-D-lysine coated plates at a density of 15,000 cells/20 µL/well in DMEM containing 10% dialyzed FBS, 20 mM HEPES, 1 mM sodium pyruvate, and 100 units/mL penicillin/streptomycin. The cells were incubated overnight at 37 °C in the presence of 5% CO_2.

The next day, GIRK assay buffer (Hank's balanced salt solution (HBSS), 20 mM HEPES, and 4.16 mM sodium bicarbonate, pH 7.4) was prepared and used to dilute compounds and Thallos-acetomethoxyester (Thallos-AM, Ion Biosciences), a fluorescent thallium indicator. Compounds were serially diluted 1:3 into 10-point concentration response curves in DMSO using a Bravo Liquid Handler (Agilent, Santa Clara, CA), transferred to a 384 well daughter plates using an Echo acoustic liquid handler (Beckman Coulter, Indianapolis, Indiana), and diluted in assay buffer to a 2X final concentration. A 2X dye solution (1.36 µM) was prepared by mixing a 2.97 mM Thallos-AM stock in DMSO with 10% (w/v) pluronic acid F-127 in a 1:1 ratio in assay buffer. The agonist plates were prepared using glutamate concentrations for the EC_{20} and EC_{Max} responses to a 5X final concentration in thallium stimulation buffer (125 mM $NaHCO_3$, 1.8 mM $CaSO_4$, 1 mM $MgSO_4$, 5 mM glucose, 12.5 mM Tl_2SO_4, 10 mM HEPES, pH 7.4). Using a microplate washer (BioTek, Winooski, VT), cells were washed with GIRK assay buffer 3 times to remove media. After the final wash, 20 µL of assay buffer remained in the cell plates. Immediately, 20 µL of the 2X dye solution (final 0.68 µM) was added to each well of the cell plate using a Multidrop Combi dispenser (Thermo Fisher, Waltham, MA). After cells were incubated with the dye solutions for 60 min at room temperature, the dye solutions were removed and replaced with GIRK assay buffer using a microplate washer, leaving 20 µL of assay buffer in the cell plate. The compound, agonist, and cell plates were placed inside the Functional Drug Screening System (FDSS 7000 or µCell kinetic imaging plate reader, Hamamatsu, Japan) to measure the thallium flux using a double add

protocol. After establishment of a fluorescence baseline (excitation, 480 nm; emission, 530 nm), 20 μL (2x) of test compound was added to the cells at 2 seconds and the response was measured. 140 seconds later, 10 μL (5x) of an EC_{20} concentration of glutamate or vehicle in thallium stimulus buffer was added to the cells, and the response of the cells was measured for an additional 158 seconds. Multiple reference wells containing no compound (DMSO vehicle) received either no glutamate (for a baseline reference), EC_{20} glutamate, or EC_{Max} glutamate (for normalization to maximum response). Fluorescence was measured throughout the experiment at a frequency of 1 measurement per 2 seconds before the glutamate/thallium addition and a frequency of 1 measurement per 1 second after the glutamate/thallium addition. Each compound concentration series occurred one time in each plate, and replicates from two or three plates were used in each experimental run. Thallium solutions and plastic solid waste were handled and disposed of according to guidelines from the Vanderbilt University Chemical Safety department. Data were normalized using a static ratio function (F/F_0) by dividing every fluorescent measurement by the initial fluorescent value for the corresponding well. The increase in signal resulting from the glutamate/thallium addition of the second add was measured by determining the slope from the time window of 145 seconds to 155 seconds. The average of all baseline slopes (no compound, no glutamate) was determined and this value was subtracted from all other slope values. The average of all EC_{Max} slopes was determined, and this value was used to normalize the baseline-corrected slopes to a percent max value (%E_{Max}). Compound-evoked increases in thallium flux response in the presence of glutamate EC_{20} agonist were determined as potentiator activity, and potency (EC_{50}) and maximum responses (% Glu_{Max}) of compounds were determined using a four-parameter logistical equation using GraphPad Prism (La Jolla, CA) or the Dotmatics software platform (Woburn, MA):

$$y = bottom + \frac{top - bottom}{1 + 10^{(LogEC50 - A)Hillslope}}$$

where A is the molar concentration of the compound; bottom and top denote the lower and upper plateaus of the concentration-response curve; HillSlope is the Hill coefficient that describes the steepness of the curve; and EC_{50} is the molar concentration of compound required to generate a response halfway between the top and bottom.

In vitro and *in vivo* DMPK Methods

Plasma protein binding: The protein binding of each compound was determined in plasma via equilibrium dialysis employing RED Plates (ThermoFisher Scientific, Rochester, NY). Plasma was added to the 96 well plate containing test compound and mixed thoroughly for a final concentration of 5 μM. Subsequently, an aliquot of the plasma-compound mixture was transferred to the *cis* chamber (red) of the RED plate, with an phosphate buffer (25 mM, pH 7.4) in the *trans* chamber. The RED plate was sealed and incubated for 4 hours at 37°C with shaking. At completion, aliquots from each chamber were diluted 1:1 with either plasma (*cis*) or buffer (*trans*) and transferred to a new 96 well plate, at which time ice-cold acetonitrile containing internal standard (50 ng/mL carbamazepine) (2 volumes) was added to extract the matrices. The plate was centrifuged (3000 rcf, 10 min) and supernatants transferred and diluted 1:1 (supernatant: water) into a new 96 well plate, which was then sealed in preparation for LC/MS/MS analysis. Each compound was assayed in triplicate within the same 96-well plate. Fraction unbound was determined using the following equation

$$F_u = \frac{Conc_{buffer}}{Conc_{plasma}}$$

Intrinsic clearance: Human or rat hepatic microsomes (0.5 mg/mL) and 1 μM test compound were incubated in 100 mM potassium phosphate pH 7.4 buffer with 3 mM $MgCl_2$ at 37°C with constant shaking. After a 5 min preincubation, the reaction was initiated by addition of NADPH (1 mM). At selected time intervals (0, 3, 7, 15, 25, and 45 min), aliquots were taken and subsequently placed into a 96-well plate containing cold acetonitrile with internal standard (50 ng/mL carbamazepine). Plates were then centrifuged at 3000 rcf (4° C) for 10 min, and the supernatant was transferred to a separate 96-well plate and diluted 1:1 with water for LC/MS/MS analysis. The *in vitro* half-life ($T_{1/2}$, min, Eq. 1), intrinsic clearance (CL_{int}, mL/min/kg, Eq. 2) and subsequent predicted hepatic clearance (CL_{hep}, mL/min/kg, Eq. 3) was determined employing the following equations:

(1) $$T_{1/2} = \frac{Ln(2)}{k}$$

where k represents the slope from linear regression analysis of the natural log percent remaining

of test compound as a function of incubation time

(2) $$CL_{\text{int}} = \frac{0.693}{in\,vitro\,T_{1/2}}\,x\,\frac{mL\ incubation}{mg\ microsomes}\,x\,\frac{45\,mg\,microsomes}{gram\ liver}\,x\,\frac{20^{a}\ gram\ liver}{kg\ body\ wt}$$

[a]scale-up factors: of 20 (human) or 45 (rat)

(3) $$CL_{hep} = \frac{Q_h \cdot CL\text{int}}{Q_h + CL\text{int}}$$

where Q_h (hepatic blood flow, mL/min/kg) is 21 (human) or 70 (rat).

LC/MS/MS Bioanalysis of Samples from Plasma Protein Binding and Intrinsic Clearance Assays. Samples were analyzed on a Thermo Electron TSQ Quantum Ultra triple quad mass spectrometer (San Jose, CA) via electrospray ionization (ESI) with two Themo Electron Accella pumps (San Jose, CA), and a Leap Technologies CTC PAL autosampler (Carrboro, NC). Analytes were separated by gradient elution on a dual column system with two Thermo Hypersil Gold (2.1 x 30 mm, 1.9 µm) columns (San Jose, CA) thermostated at 40°C. HPLC mobile phase A was 0.1% formic acid in water and mobile phase B was 0.1% formic acid in acetonitrile. The gradient started at 10% B after a 0.2 min hold and was linearly increased to 95% B over 0.8 min; hold at 95% B for 0.2 min; returned to 10% B in 0.1 min. The total run time was 1.3 min and the HPLC flow rate was 0.8 mL/min. While pump 1 ran the gradient method, pump 2 equilibrated the alternate column isocratically at 10% B. Compound optimization, data collection and processing was performed using Thermo Electron's QuickQuan software (v2.3) and Xcalibur (v2.0.7 SP1).

Inhibition of Cytochrome P450 Enzymes: A cocktail of substrates for cytochrome P450 enzymes (1A2: Phenacetin, 10 µM; 2C9: Diclofenac, 5 µM; 2D6: Dextromethorphan, 5 µM; 3A4: Midazolam, 2 µM) were mixed for cocktail analysis. For P450 2C19, the substrate stock (Mephenytoin, 40 µM)and substrate mix were prepared separately for discrete analysis. The positive control for pan-P450 inhibition (miconazole) was included alongside each test compound in analysis.

A reaction mixture of 100 mM Kpi, pH 7.4, 0.1 mg/mL human liver microsomes (HLM) and Substrate Mix is prepared and aliquoted into a 96-deepwell block. Test compound and positive control (in duplicate) were then added such that the final concentration of test compound ranged

from 0.1 – 30 μM. The plate was vortexed briefly and then pre-incubated at 37°C while shaking for 15 minutes. The reaction was initiated with the addition of NADPH (1 mM final concentration). The incubation continued for 8 min and the reaction quenched by 2x volume of cold acetonitrile containing internal standard (50 nM carbamazepine). The plate was centrifuged for 10 minutes (4000 rcf, 4°C) and the resulting supernatant diluted 1:1 with water for LC/MS/MS analysis. A 12 point standard curve of substrate metabolites over the range of 0.98 nM to 2000 nM.

Samples were analyzed via electrospray ionization (ESI) on an AB Sciex API-4000 (Foster City, CA) triple-quadrupole instrument that was coupled with Shimadzu LC-10AD pumps (Columbia, MD) and a Leap Technologies CTC PAL auto-sampler (Carrboro, NC). Analytes were separated by gradient elution using a Fortis C18 3.0 x 50 mm, 3 μm column (Fortis Technologies Ltd, Cheshire, UK) thermostated at 40°C. HPLC mobile phase A was 0.1% formic acid in water (pH unadjusted), mobile phase B was 0.1% formic acid in acetonitrile (pH unadjusted). The gradient started at 10% B after a 0.2 min hold and was linearly increased to 90% B over 1.2 min; held at 90% B for 0.1 min and returned to 10% B in 0.1 min followed by a re-equilibration (0.9 min). The total run time was 2.5 min and the HPLC flow rate was 0.5 mL/min. The source temperature was set at 500°C and mass spectral analyses were performed using multiple reaction monitoring (MRM), with transitions specific for each compound utilizing a Turbo-Ionspray® source in positive ionization mode (5.0 kV spray voltage).

The IC_{50} values for each compound were obtained for the individual CYP enzymes by quantitating the inhibition of metabolite formation for each probe substrate. A 0 μM compound condition (or control) was set to 100% enzymatic activity and the effect of increasing test compound concentrations on enzymatic activity could then be calculated from the % of control activity. Curves were fitted using XLfit 5.2.2 (four-parameter logistic model, equation 201) to determine the concentration that produces half-maximal inhibition (IC_{50}).

In vivo DMPK experimental

Compounds were formulated as 10% tween 80 micro suspensions in sterile water at the concentration of 1 mg/ml and administered intraperitoneally to male Sprague- Dawley rats weighing 225 to 250 g (Harlan, Inc., Indianapolis, IN) at the dose of 10 mg/kg. The rat blood and brain were collected at 0.25 hr. Animals were euthanized and decapitated, and the brains were

removed, thoroughly washed in cold phosphate buffered saline and immediately frozen on dry ice. Trunk blood was collected in EDTA Vacutainer tubes, and plasma was separated by centrifugation and stored at -80°C until analysis. Plasma was separated by centrifugation (4000 rcf, 4°C) and stored at 80°C until analysis. On the day of analysis, frozen whole-rat brains were weighed and diluted with 1:3 (w/w) parts of 70:30 isopropanol:water. The mixture was then subjected to mechanical homogenation employing a Mini-Beadbeater™ and 1.0 mm Zirconia/Silica Beads (BioSpec Products) followed by centrifugation. The sample extraction of plasma (20 μL) or brain homogenate (20 μL) was performed by a method based on protein precipitation using three volumes of ice-cold acetonitrile containing an internal standard (50 ng/mL carbamazepine). The samples were centrifuged (3000 rcf, 5 min) and supernatants transferred and diluted 1:1 (supernatant: water) into a new 96 well plate, which was then sealed in preparation for LC/MS/MS analysis.

In vivo samples were analyzed via electrospray ionization (ESI) on an AB Sciex API-5500 QTrap (Foster City, CA) instrument that was coupled with Shimadzu LC-20AD pumps (Columbia, MD) and a Leap Technologies CTC PAL auto-sampler (Carrboro, NC). Analytes were separated by gradient elution using a Fortis C18 3.0 x 50 mm, 3 μm column (Fortis Technologies Ltd, Cheshire, UK) thermostated at 40°C. HPLC mobile phase A was 0.1% formic acid in water (pH unadjusted), mobile phase B was 0.1% formic acid in acetonitrile (pH unadjusted). The gradient started at 30% B after a 0.2 min hold and was linearly increased to 90% B over 0.8 min; held at 90% B for 0.5 min and returned to 30% B in 0.1 min followed by a re-equilibration (0.9 min). The total run time was 2.5 min and the HPLC flow rate was 0.5 mL/min. The source temperature was set at 500°C and mass spectral analyses were performed using multiple reaction monitoring (MRM), with transitions specific for each compound utilizing a Turbo-Ionspray® source in positive ionization mode (5.0 kV spray voltage). The calibration curves were constructed in blank plasma. All data were analyzed using AB Sciex Analyst software v1.5.1.

Behavioral Pharmacology Methods

Animals: Adult male Sprague-Dawley rats (Envigo, Inc., Indianapolis, IN) were used. They were housed in the animal care facility certified by the American Association for the Accreditation of Laboratory Animal Care (AALAC) under a 12-hour light/dark cycle (lights on: 7 a.m.; lights off: 7 p.m.) and had free access to food and water. The experimental protocols performed during the light cycle were approved by the Institutional Animals Care and Use Committee of Vanderbilt University and conformed to the guidelines established by the National Research Council Guide for the Care and Use of Laboratory Animals.

Novel Object Recognition: The rats were habituated for 10 minutes to the novel object recognition (NOR) arenas for two consecutive days prior to testing. The NOR arena consisted of a dark-colored plexiglass box (40 X 64 X 33 cm) and was cleaned with 70% ethanol between animals. On day 3 the rats were habituated to the dosing room for 2 hours and then administered vehicle (0.5% natrasol/0.015% Tween 80) or VU6053371 (1-10 mg/kg p.o., 10mL/kg, n=13-16) and were placed back into their homecage for 60 min. The rats were then placed into the NOR arena with 2 identical objects for ten minutes and then placed back into the home cage. The rats were then placed back into the NOR arena with one of the familiar objects replaced with a novel object at 24 hrs after the familiar object exposure. The activity of the animals was recorded for 5 min and the time spent exploring each of the objects is scored by a blinded observer. Recognition index was calculated as [(time spent exploring novel object) – (time spent exploring familiar object)]/total time exploring objects.

Toxicity Study Methods

Animals

An exploratory toxicity study was performed in male Crl:CD(SD) rats, adhering to the animal welfare regulations of Boehringer Ingelheim and local authorities (Regierungspräsidium Tübingen, Baden-Württemberg, Germany). The a*nimals wer*e sourced from Charles River Laboratories Italy, Research Models and Services, located in Calco (Lecco), Italy. On Day 1, animals were approximately eight weeks old, with body weights ranging from 258.3. to 332.9 grams. Rats were housed in groups of up to five per cage (type 2000 P), with cage changes at least once weekly. Softwood bedding (FS14; J. Rettenmaier & Soehne GmbH & Co. KG, Rosenberg, Germany) was used, and enrichment included wooden gnawing sticks (J. Rettenmaier & Soehne GmbH & Co. KG, Rosenberg, Germany), raised seating platforms, and one additional standard food pellet per animal per week. Standard pellet diet (Altromin 1328 Forti, Altromin Spezialfutter GmbH & Co. KG, Lage, Germany) and municipal tap water were provided ad libitum. Animals acclimatized to the environmental conditions for approximately two weeks, which included a one-week pre-treatment period.

Study Design: BI03738809XX Exploratory 1-week oral (gavage) toxicity study (non-GLP) in male rats

BI 3738809 (batch 74661088 / 7; formulated 0.5% (w/w) Natrosol® 250 HX) and 0.015% (w/w) polysorbate 80 (Tween® 80) in highly purified water) was twice daily administered (8/16 h apart) at dose levels of 0, 30, 100 and 300 mg/kg (0, 60, 200 and 600 mg/kg/day) by oral gavage to groups of 5 male Crl:CD(SD) rats for 9 consecutive days (including the day of necropsy). In addition, three toxicokinetic satellite animals per group were administered with the identical dosing regimen. A full toxicokinetic plasma exposure profile was performed on Days 1 and 7 in satellite animals. On the day of necropsy, animals were dosed in the morning and sacrificed at ~tmax and plasma, muscle and brain tissue were sampled for exposure analysis from main study animals. In main study animals, mortality, clinical observations, body weight and food/water consumption data were recorded regularly. In addition, a modified Irwin test was included on Days 1 and 3 (0.5, 3 and 24 h post-dose). Clinical Pathology was performed prior to necropsy.

Main study animals were subjected to complete necropsy, organ weight recording and histopathological investigations were performed.
